# Extended BODIPYs as Red–NIR Laser Radiation Sources with Emission from 610 nm to 750 nm

**DOI:** 10.3390/molecules28124750

**Published:** 2023-06-13

**Authors:** Ainhoa Oliden-Sánchez, Enrique Alvarado-Martínez, Diana E. Ramírez-Ornelas, Miguel A. Vázquez, Edurne Avellanal-Zaballa, Jorge Bañuelos, Eduardo Peña-Cabrera

**Affiliations:** 1 Departamento de Química Física, Universidad del País Vasco (UPV/EHU), Barrio Sarriena s/n, Aptado 644, 48940 Leioa, Bizkaia, Spain; ainhoa.oliden@ehu.es (A.O.-S.); edurne.avellanal@ehu.es (E.A.-Z.); 2 Departamento de Química, Universidad de Guanajuato, Noria Alta s/n, Guanajuato 36050, Guanajuato, Mexico; e.alvaradomartinez@ugto.mx (E.A.-M.); ramirez.de@ugto.mx (D.E.R.-O.); mvazquez@ugto.mx (M.A.V.)

**Keywords:** BODIPY, charge transfer, dyes/pigments, fluorescence, laser spectroscopy

## Abstract

Herein, we report the synthetic access to a set of π-extended BODIPYs featuring a penta-arylated (phenyl and/or thiophene) dipyrrin framework. We take advantage of the full chemoselective control of 8-methylthio-2,3,5,6-tetrabromoBODIPY when we conduct the Liebeskind–Srogl cross-coupling (LSCC) to functionalize exclusively the *meso*-position, followed by the tetra-Suzuki reaction to arylate the halogenated sites. All these laser dyes display absorption and emission bands in the red edge of the visible spectrum reaching the near-infrared with thiophene functionalization. The emission efficiency, both fluorescence and laser, of the polyphenylBODIPYs can be enhanced upon decoration of the peripheral phenyls with electron donor/acceptor groups at *para* positions. Alternatively, the polythiopheneBODIPYs show an astonishing laser performance despite the charge transfer character of the emitting state. Therefore, these BODIPYs are suitable as a palette of stable and bright laser sources covering the spectral region from 610 nm to 750 nm.

## 1. Introduction

The quest for novel far-red and near-infrared (NIR) fluorophores is an emerging field in dye chemistry [1,2,3]. Nowadays, fluorescent imaging has become a powerful tool to visualize biological systems and track biomolecules under advanced fluorescent microscopes with super resolution (single molecule) [4,5,6]. This technique demands molecular probes working in the so-called biological or therapeutic window (beyond 650 nm), owing to the deep-tissue penetration of this long-wavelength radiation (2–5 cm) [7,8,9,10]. These fluorophores should be chemically robust and photostable in order to display long lasting fluorescent images, and readily available for post-functionalization to allow targetable labelling [11]. Another aspect of these red–NIR luminophores is the development of low-cost excitation sources in optical fibers applied in telecommunications (telecom range beyond 800 nm) owing to the lower interference and scattering of this long-wavelength radiation with its surroundings, allowing the light to travel longer distances [12,13].

Among the various classes of organic dyes tested as light emission sources in this sought after spectral window (mainly based on cyanine, porphyrin and phtalocyanine skeletons) [14,15], BODIPY derivatives are in the spotlight [16,17]. This modern fluorophore, a structural analogue to porphyrin, enables application of several synthetic strategies to push the absorption and emission towards longer wavelengths while retaining their excellent photophysical signatures [18,19,20]. According to the literature, several approaches have been successfully tested to span the delocalized π-system of the dipyrrin core and reach the red–NIR spectral window. The main strategies involve the fusion of aromatic rings (aryls and heterocycles, and even other BODIPYs) [21,22,23], peripheral linkage of aromatic frameworks (for instance, styryls and the aforementioned cyclic moieties) [24,25,26], replacement of the *meso*-carbon by nitrogen (aza-BODIPYs) [27,28], grafting electron releasing and withdrawing functionalizations (push-pull BODIPYs) [29,30], and the combination of some of these molecular strategies into a single structure [31]. From such surveys, we find that the tethering of aromatic frameworks, properly functionalized with electron rich groups, at the pyrrolic β and α positions, is one of the successful strategies to induce pronounced spectral shifts, while retaining the high absorption and emission efficiency characteristics of this family of dyes. Moreover, it would be highly advantageous to have full control of the functionalization of the different BODIPY positions to be able to tailor the physical (e.g., solubility) and photophysical properties of the final products.

**Scheme 1:**
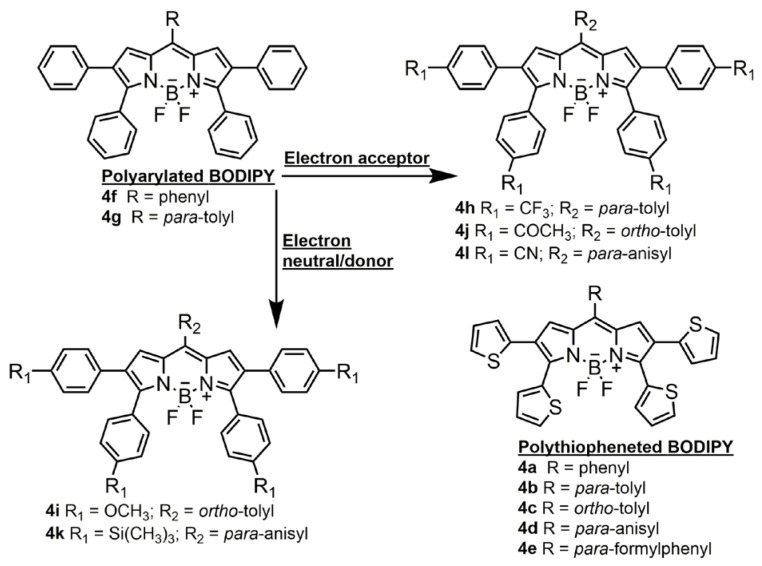
Molecular structure of the red–NIR emitting BODIPYs.

In respect of this background, we synthesized two sets of 8-arylBODIPY-based fluorophores (Scheme 1). On the one hand, we synthesized polyarylated BODIPYs bearing 2,3,5,6-phenyls *para*-functionalized with electron withdrawing (formyl, trifluoromethyl and cyano) and electron neutral or donor groups (trimethylsilyl and methoxy). On the other hand, we synthesized polythiopheneBODIPYs bearing such sulfur-containing heterocycles at the same above-mentioned positions. To ascertain the role of the 8-aryl group, its stereoelectronic properties were systematically varied, adding steric constraints via *ortho*-methylation or grafting electron donor (methoxy) or acceptor (formyl) groups at its *para* position. The main aim of these long-wavelength emitting π-extended BODIPYs is to decipher the structural factors that trigger the spectral shift and the emission efficiency (both fluorescence and laser) and provide a chart of fluorophores that are efficient and stable far-red–NIR laser sources. Accordingly, we have evaluated their photonic signatures under soft (photophysical properties) and hard (laser performance) irradiation regimes and rationalized them assisted by computational simulations.

## 2. Results and Discussion

### 2.1. Synthesis

**Scheme 2:**
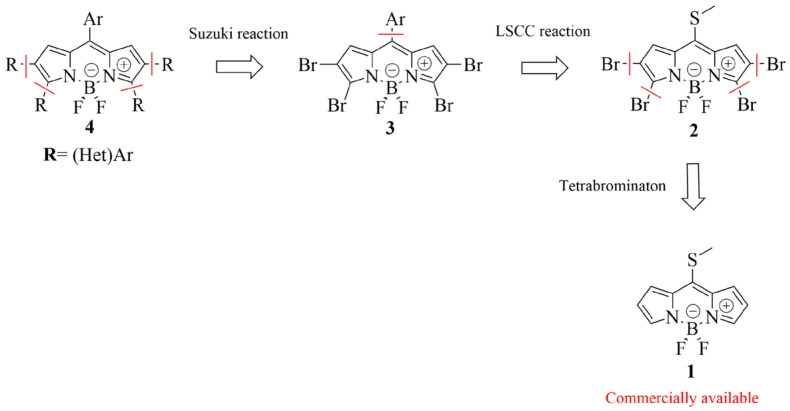
Retrosynthetic analysis for the synthesis of poly-(het)arylBODIPYs.

Recently, we reported the synthesis of novel BODIPY building block **2** through a tetrabromination reaction of commercially available 8-methylthioBODIPY **1** and demonstrated that it displayed orthogonal reactivity [32]. The C–S bond was activated under Liebeskind–Srogl cross-coupling reaction (LSCC) conditions, leaving the C–Br bonds intact [33]. This result allowed the selective functionalization of the *meso*-position leaving the other brominated positions available to be manipulated at will. In this context, we decided to evaluate a quadruple Suzuki–Miyaura cross-coupling reaction to obtain a new family of poly (het)arylBODIPY dyes (Scheme 2).

**Scheme 3:**
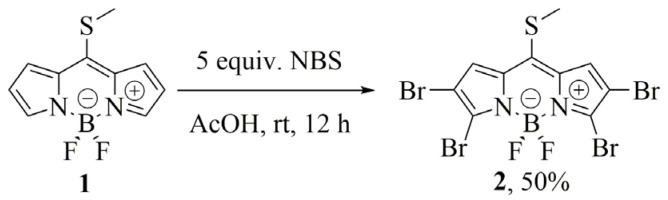
Synthesis of the tetrabrominated 8-methylthioBODIPY precursor **2**.

We initially synthesized BODIPY **2** according to Scheme 3.

**Scheme 4:**
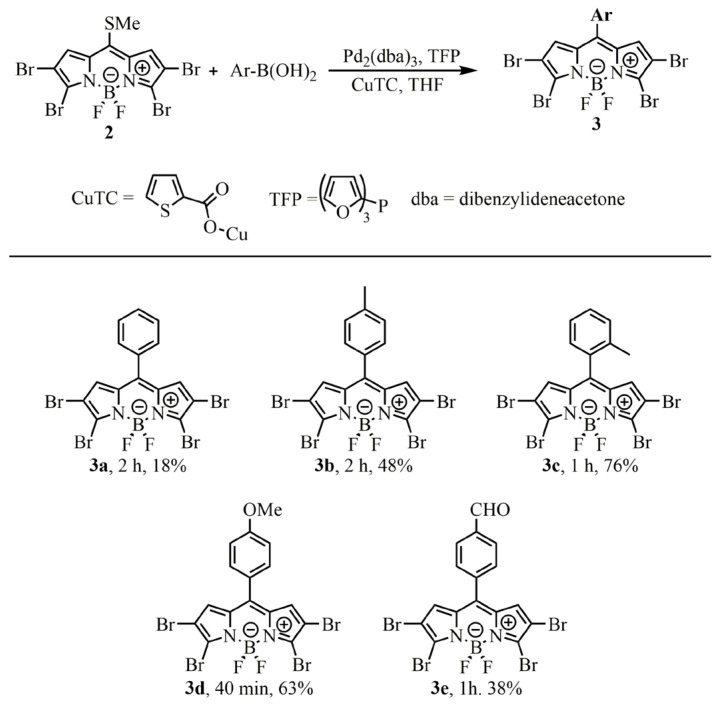
Synthesis of tetrabrominated *meso*-arylBODIPYs via LSCC reaction, structure of the red–NIR emitting BODIPYs. Conditions: BODIPY **2** (1 equiv.), boronic acid (3 equiv.), Pd_2_(dba)_3_ (2.5 mol%), TFP (7.5 mol%), CuTC (3 equiv.) in THF (0.03 M) at 55 °C.

With **2** in hand, a chemoselective LSCC reaction was carried out to access tetrabrominated *meso*-arylBODIPYs **3** according to Scheme 4. Several arylboronic acids were evaluated, accessing five derivatives in modest to good yields (18–76%) in short reaction times.

Next, the key step of our synthetic plan was conducted, that is, the multiple functionalization of the brominated positions via the Suzuki–Miyaura cross-coupling reaction in **3a** (Table S1). The reaction conditions used in entry 1 and 2 were reported in the literature for the multiple functionalization of BODIPYs [34,35]. For entry 1, the reaction was incomplete, and multiple products were observed, while for entry 2, the desired product **4f** was isolated in 42% yield. When we used Pd(OAc)_2_ and SPhos, along with K_3_PO_4_ as a base, the yield of **4f** increased to 52% (entry 3 in Table S1).

Once the best conditions were determined, we set out to react **3a**–**e** with several (het)arylboronic acids of different electronic natures to study the scope of the Suzuki–Miyaura cross-coupling reaction. The results obtained for the multiple functionalization of **3a**–**e** are shown in Table 1. Multiple Suzuki–Miyaura cross-coupling reactions took place smoothly and gave the poly (het)arylated products in modest to good yields (41 to 83%) in relatively short reaction times. In general, electron-rich boronic acids gave the highest yields (entries 1–4, 9 and 11 in Table 1) and shortest reaction times. Specifically, the best results, both in yield (65 to 83%) and reaction times, were obtained using 2-thienylboronic acid, since the reactions took only 20 min. While the electron-poor boronic acids required longer reaction times (entries 10 and 12 in Table 1). No clear trend was observed when the *meso*-aryl substituents were modified.

### 2.2. Photophysical Properties

**Figure 1:**
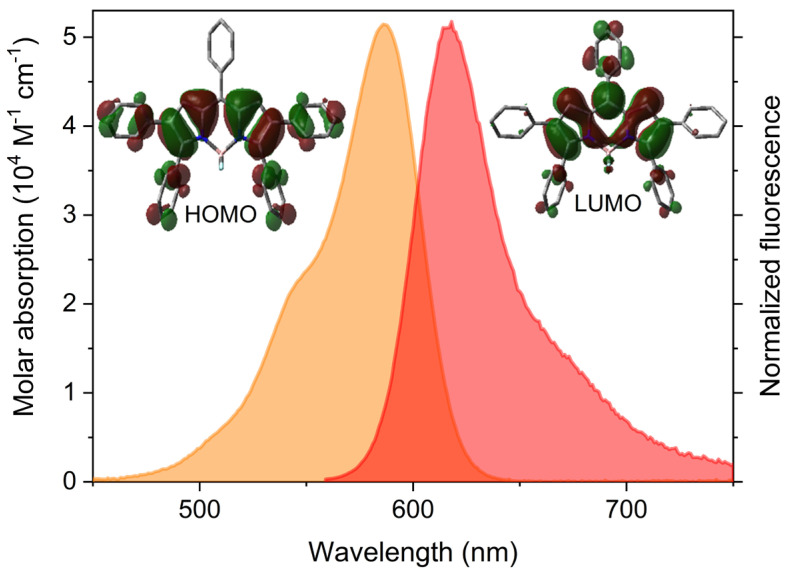
Representative absorption (orange) and fluorescence (red) spectra of the polyphenylBODIPY **4f** (see Figure S1 for the spectra of the rest of dyes of this family) in diluted solutions of cyclohexane. The corresponding contour maps of the frontier orbitals are also enclosed.

The arylation of the pyrroles extends the chromophoric delocalized π-system pushing the spectral bands towards the red edge of the visible (absorption and emission of **4f** and **4g** placed at around 590 and 620 nm, respectively, Figure 1). The *meso*-phenyl does not take part in the delocalized framework of the dipyrrin owing to its twisted geometrical arrangement (theoretically predicted torsion angle around 70° from ground state optimized geometries) imposed by steric reasons. The attained fluorescence efficiencies are lower (around 30%, Table 2) than those usually reported for green–yellow emitting BODIPYs (usually higher than 80%). This trend is related to the free motion of the peripheral phenyls, which enhances the non-radiative relaxation channels related with conformational freedom (note the unusually high Stokes shift, around 1000 cm^−1^, suggesting geometric relaxation upon excitation). With this line of reasoning, previous publications dealing with heptaphenylated BODIPYs reported similar values of fluorescence efficiencies and pinpointed internal conversion as the main reason [36].

**Figure 2:**
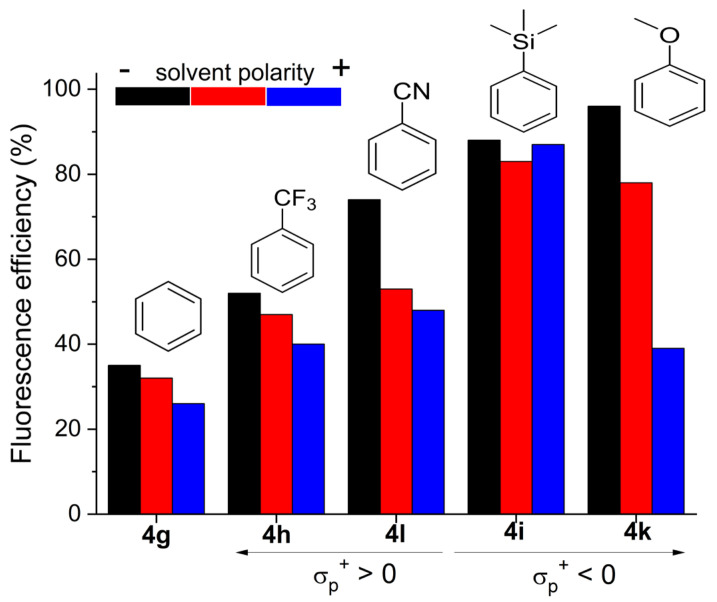
Evolution of the fluorescent efficiency in media of different polarity for representative polyphenylBODIPYs bearing electron acceptor or electron donor moieties at the *para* position of the peripheral pyrrolic phenyls.

In this regard, the decoration of the aryls grafted on the chromophoric pyrroles with electron withdrawing or electron releasing moieties at the *para*-position is a suitable approach to ameliorate the fluorescent response (Table 2 and Figure 2). The attachment of electron acceptor groups does not alter the spectral band positions, but progressively enhances the fluorescence efficiency, being twice that of the former ones for the dyes bearing carbonyl (**4j**, Hammet parameter σ_p_^+^ = 0.55) and trifluoromethyl (**4h**, σ_p_^+^ = 0.61), and even reaching an efficiency of up to 75% for the dye bearing cyano (**4l**, σ_p_^+^ = 0.66), the strongest electron withdrawing unit among the ones tested so far (Figure 2). It is worth mentioning that the coexistence of this last group with *para*-methoxy in the 8-phenyl increases the charge separation, explaining the sensitivity of the fluorescent response of **4l** with the solvent polarity (down to 48% in acetonitrile, Figure 2).

It is likely that this functionalization of the aryls with electron rich groups enhances the delocalization between the dipyrrin and the phenyls, leading to less single bond character in the linkage and thereby decreasing the free motion of the peripheral rings. In fact, theoretically optimized geometries at the ground state reveal that both the twisting angle and the bond length connecting the phenyls and the pyrroles, decrease as the aryls are *para*-functionalized (average values from 45° to 35° and from 1.45 Å to 1.40 Å, respectively). Further evidence of the success of this strategy to reduce internal conversion is provided by **4i** and **4k** (Figure 2). Even the sole presence of the electronically neutral trimethylsilane (**4i**, σ_p_^+^ = 0.02) yields a remarkable increase in the fluorescence that approaches 90%. Moreover, the electron donor methoxy (**4k**, σ_p_^+^ = −0.78) induces a further bathochromic shift and renders notable fluorescence efficiencies reaching almost 100% (Figure 2). However, in this case, the four electron donor units decorating the electron deficient boron–dipyrrin core were able to induce charge separation, as suggested by the solvent polarity triggered decrease in the fluorescence output (Figure 2), and the unexpected positive solvatochromism of the fluorescence band, opposite to the negative solvatochromism registered for the absorption band (Table S2). Therefore, *para*-functionalization of the phenyls decorating the chromophoric pyrroles is a good strategy to enhance the fluorescence signal of poliphenylBODIPYs.

**Figure 3:**
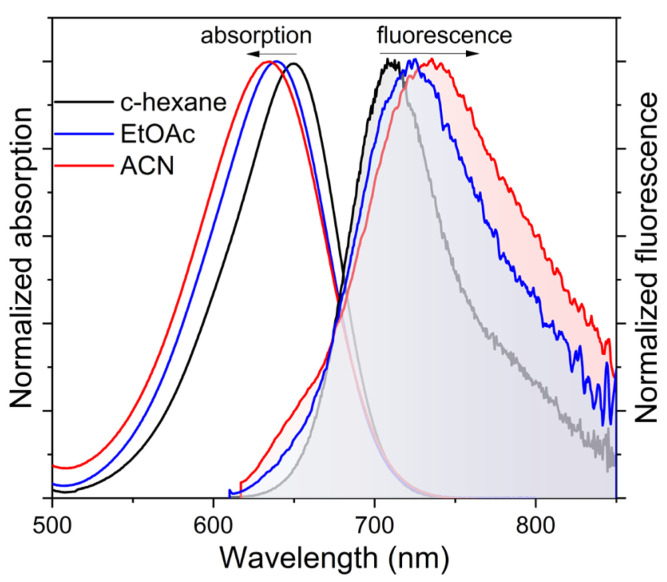
Normalized absorption and fluorescence (shadow filled) spectra of **4e** as a representative dye of the polythiopheneBODIPYs (see Figure S2 for the spectra of the rest of dyes of this family) in diluted solutions.

To further push the spectral bands and reach the far-red–NIR region the pyrrolic phenyls were replaced by thiophene, featuring higher electron releasing ability (σ_p_^+^ = −0.43). In fact, the absorption band of the polythiopheneBODIPYs is placed at around 630–650 nm, depending on the *meso*-phenyl substitution (Table 3). Thus, the highest bathochromic shift is achieved upon formylation of the 8-phenyl (**4e**), since electron withdrawing groups at the *meso* position further stabilize the LUMO owing to its high electronic density located at the chromophoric position (Figure 1). Accordingly, the fluorescence band is placed around 680–710 nm, being again the formylated dye **4e**, the one displaying the reddest emission (Figure 3). Nevertheless, an increase in the solvent polarity markedly affected the fluorescence signatures of this set of polythiopheneBODIPYs. Overall, the fluorescence efficiency was lower (around 15 to 20%, with the exception of **4d**, which reached almost 40%), than for the polyarylated analogues in apolar media. An increase in the solvent polarity implies a pronounced quenching of the emission, as reflected in the low efficiencies (down to 2%) and fast lifetimes (hundreds of picoseconds) recorded (Table 3 vs. Table 2). Moreover, the fluorescent band displays a marked bathochromic shift (around 30 nm) with the solvent polarity (Table 3 and Figure 3). Such an environment stabilizes the charge separation and a dark charge separated state (CS) can be populated. Thus, **4e** showing push–pull features, emits at 735 nm in polar media (Figure 3), the reddest emission herein recorded, but with low fluorescence (just 2%, Table 3). Therefore, the electron releasing ability of the thiophenes is able to induce charge transfer (CT) character to the emitting state, becoming a state of high polarity. 

**Figure 4:**
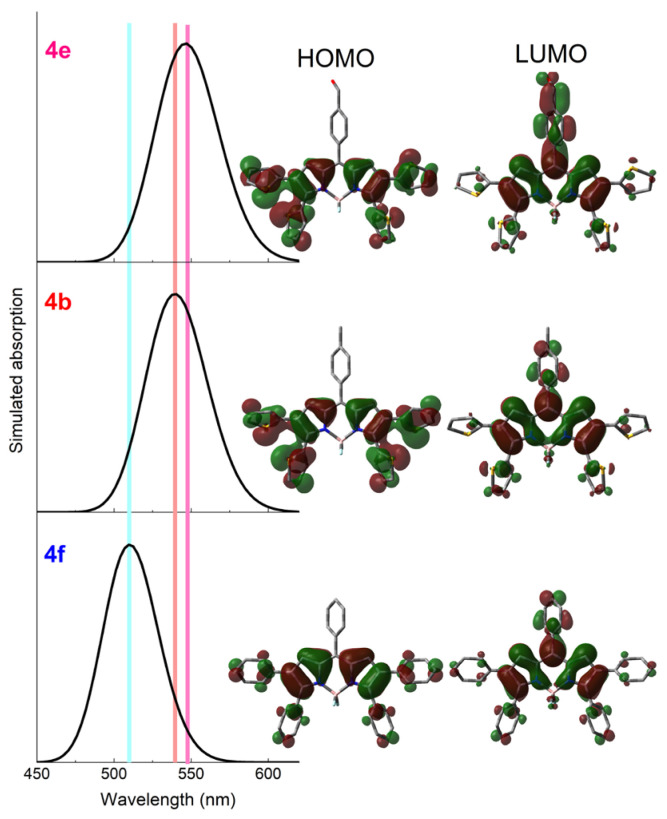
Contour maps of the frontier molecular orbitals calculated from optimized ground state geometries (wb97xd/6-311 + g*) for representative polyphenylBODIPY **4f**, and polythiopheneBODIPYs **4b** and **4e**. The corresponding theoretically predicted absorption spectra (td wb97xd) are also depicted.

Such a feature can be envisaged from Figure 4. The grafting of electron rich thiophene leads to a more extended π-system, sustained by the simulated HOMO contour map, where the π-electronic density is clearly spanned over the whole molecule, comprising both BODIPY and thiophenes. However, the excitation implies a marked electronic transfer from the thiophenes to the BODIPY core, as supported by the theoretically predicted LUMO, which is mainly located at the latter moiety. Furthermore, upon formylation of the 8-phenyl, the shift of the electronic density towards the BODIPY is more pronounced, reaching the 8-formylated ring, owing to the electron withdrawing effect of this functionalization. Thus, this electronic rearrangement upon excitation anticipates the CT character of the emitting state, strengthened in **4e**. Therefore, polythiopheneBODIPYs enable us to push the emission deeper into the far-red–NIR region (beyond 700 nm), but in turn the efficiency decreases, owing to the lower energy gap and the solvent exerted stabilization of charge separation.

Motivated by the ongoing CT upon attachment of thiophene, we tested the viability of these polythiopheneBODIPYs as halogen-free singlet oxygen photosensitizers. One of the most currently active in photodynamic therapy is the promotion of CT as an intermediate to reach the target triplet state, which enables singlet oxygen generation [37]. In fact, thiophene-fused BODIPYs were reported to photosensitize singlet oxygen through ICT-mediated intersystem crossing [38,39]. However, in the herein reported BODIPYs bearing directly linked tiophenes, no singlet oxygen emission was detected, neither under drastic conditions (dye concentration, excitation sources) nor in different solvents where the singlet oxygen lifetime is longer (toluene, chloroform). It is noteworthy that the triplet state population from a CT implies a subtle balance between CS and charge recombination (CR). In other words, enough CS should be promoted to stabilize the CT state, but at the same time, the CR probability should be high enough to afterwards populate the triplet state [40]. In the BODIPYs described here, such restrained balance is displaced to CS owing to the simultaneous presence of four electron donor peripheral thiophenes. Thus, the triplet state is not reached from the CT and the excitation energy is dissipated through non-radiative funnels, leading to fluorescence quenching and no singlet oxygen generation.

### 2.3. Laser Properties

**Figure 5:**
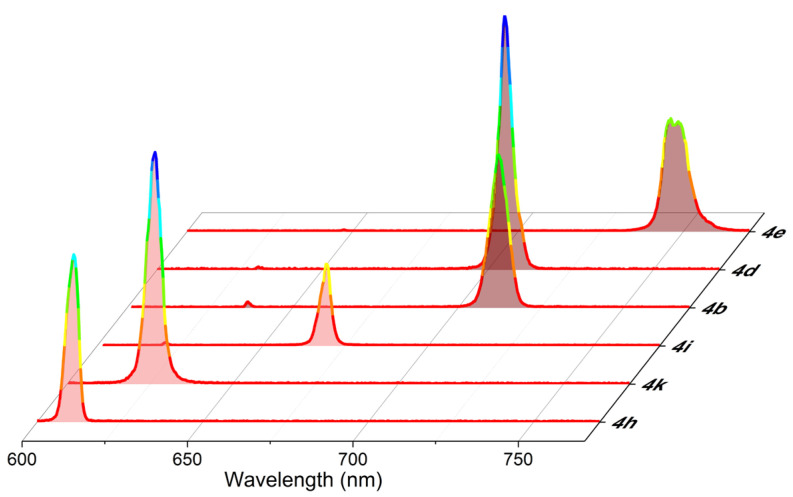
Laser spectra scaled by the maximum efficiency of representative polyphenylBODIPYs and polythiopheneBODIPYs in ethyl acetate to highlight the covered spectral window.

The absence of population of the triplet manifold prompted us to test these extended BODIPYs as photoactive media of tunable organic lasers. All of them show the typical broad dye laser emission (full width at half maximum, FWHM, overall around 5 nm) in the red–NIR edge of the visible, ranging from 600 nm to 760 nm (Figure 5), after being transversally pumped by a wavelength-tunable Optical Parametric Oscillator (OPO), which allows irradiation of each dye at its maximum absorption wavelength (see Section 3 for details). The concentration that optimizes the laser efficiency, understood as the ratio of the output and input (pump) energies, depends on each dye (around 0.5–0.75 mM, Tables S3 and S5). In this sense, the laser output efficiency increases with the concentration up to a maximum plateau, from which it decreases owing to reabsorption/reemission phenomena. The shift of the emission peak to longer wavelengths with the concentration increase backs up this interpretation (Tables S3 and S5). In general terms, the evolution of the laser wavelength correlates well with the fluorescent one (Table 2 and Table 3). Thus, within the polyarylated dyes, **4i**, functionalized with methoxy, shows the reddest laser emission (from around 610–635 nm to 670 nm). Alternatively, the laser emission of the BODIPYs functionalized with thiophene is pushed deeper into the red (around 705–715 nm), mainly with the presence of formyl in the 8-phenyl (**4e**, up to 745 nm), being the reddest one among all the tested laser dyes.

The polyphenylated dye **4f**, shows a laser efficiency approaching 10%, which is ameliorated upon *para*-substitution of the pyrrolic phenyls. Thus, the functionalization with cyano (**4l**), trifluoromethyl (**4h**) and trimethylsilane (**4k**) increases the laser action (up to almost 18% for the latter, being almost twice that of the reference dye **4f**), in good correlation with the observed increase in the fluorescent response of these dyes (Table 2). The exception to the rule is **4i**, where in spite of its high fluorescent efficiency, the laser efficiency decreases. Likely, the charge transfer induced by the methoxy groups could account for this apparent mismatch. On the other hand, the polythiopheneBODIPYs show an unexpectedly high laser efficiency in view of the recorded low fluorescence efficiencies (Table 3). Thus, they show a more structured and broader (FWHM up to 10 nm) band with an efficiency around 10%, reaching 20% for **4d**, in line with the higher fluorescence response recorded for this dye. We should bear in mind that this set of dyes are characterized by larger Stokes shifts (around 1500 cm^−1^, Table S4), which decrease the reabsorption/reemission phenomena, and hence the losses in the resonator cavity. Their emitting state is endowed with substantial CT character, leading to lower fluorescence efficiencies but shorter lifetimes (down to 1 ns). Such fast decay seems to ameliorate the population inversion and enhance the stimulated emission probability, counterbalancing their low probability of spontaneous emission. Indeed, stryryl dyes (i.e., the commercial LDS722), which are also characterized by similar low fluorescent efficiency and even shorter lifetimes, show unusually high laser efficiencies (up to 40%)which are also in the red-edge of the visible with a finely structured and broad band [41].

Regarding the photostability, described as the required energy to decrease the laser induced fluorescence (LIF) by 10% (see Section 3 for details), extremely high values are not expected owing to the conformational freedom of the molecular structure, which could enhance the dissipation of the pumping energy as heat. The trifluoromethyl (**4h**) is the most suited functionalization to enhance the stability against irradiation of polyphenylBODIPYs (Table 2). Indeed, this dye holds an optimal balance between laser efficiency (13.5%) and photostability (14.1 GJ/mol). In this sense, it is noteworthy that the low photostability of other dyes is characterized by high emission efficiencies (**4l** and mainly **4i**, both sharing 8-anisole). It has been reported that the 8-position is involved in the photobleaching mechanism, and its substitution (for instance with phenyl) is recommended to enhance photostability [42,43]. However, it seems that the *para*-functionalization of this ring with the electron donor methoxy is detrimental in terms of photostability. Alternatively, the polythiopheneBODIPYs show lower photostability, being in most of them around 6–7 GJ/mol and reaching 10 GJ/mol for **4d** (Table 3). This dye is the one with the lowest non-radiative deactivation rate constant of the series (Table S4), which is also reflected in its higher fluorescence and laser efficiency (Table 3). Interestingly, **4d** dye bears 8-anisole and the photostabilty increases in contrast to the trend observed above with polyphenylBODIPYs (**4i**). This finding reveals that it is challenging to establish general rules to understand the interplay between molecular structure and photostability. Other commercial dyes lasing in this spectral region, like the oxazine Nile Blue, maintain 90% of the laser output after enduring 10 GJ/mol, similar to the herein reported polyphenyl and polythiopheneBODIPYs, which hence can be catalogued as competitive bright and long-lasting active media for lasers.

## 3. Materials and Methods

### 3.1. Synthesis Details

^1^H and ^13^C NMR spectra were recorded on a Bruker Advance III spectrometer (500 or 400 MHz) in deuteriochloroform (CDCl_3_) with either tetramethylsilane (TMS) (0.00 ppm 1H, 0.00 ppm 13C) or chloroform (7.26 ppm 1H, 77.00 ppm ^13^C) or as internal reference unless otherwise stated. Data are reported in the following order: chemical shift in parts per million (ppm), multiplicities (br (broadened), s (singlet), d (doublet), t (triplet), q (quartet), sex (sextet), hex (hextet), m (multiplet), exch (exchangeable), and app (apparent)), coupling constants, *J* (Hz), and integration. Infrared spectra were recorded on a Perkin-Elmer-Spectrum 100 FTIR spectrophotometer. Peaks are reported (cm^−1^) with the following relative intensities: s (strong, 67–100%), m (medium 40–65%), and w (weak 20–39%). Melting points were determined on a Stanford Research Systems EZ-Melt apparatus and are not corrected. HRMS samples were ionized by ESI^+^ and recorded via the TOF method. All reactions were performed under a dry N_2_ atmosphere in oven- and or flame-dried glassware. TLC was conducted in silica gel on TLC Al foils. Detection was achieved with UV light (254 or 365 nm). Starting 8-methylthioBODIPY, CuTC, tri(2-furyl)phosphine, and boronic acids are commercially available. Solvents were dried and distilled before use.

#### 3.1.1. Typical Procedure (TP1) for the L–S Cross-Coupling Reaction

An oven-dry Schlenk tube, equipped with a stir bar, was charged with the 2,3,5,6 tetrabromo-8-methylthioBODIPY **2** (1.0 equiv.), the corresponding arylboronic acid (3.0 equiv.), and dry THF (0.03 M) under N_2_. The stirred solution was sparged with N_2_ for 10 min., whereupon CuTC (3.0 equiv.), Pd_2_(dba)_3_ (2.5 mol%), and TFP (7.5 mol%) were added under N_2_. The reaction mixture was immersed in a pre-heated oil bath at 55 °C. After TLC showed that the reaction went to completion, the reaction mixture was allowed to reach rt and was adsorbed on SiO_2_-gel and the solvent was evaporated on a rotary evaporator under vacuum. After flash-chromatography (SiO_2_-gel EtOAc/hexanes gradient) purification, 8-aryl-2,3,5,6-tetrabromoBODIPYs were obtained as highly colored solids.

#### 3.1.2. Typical Procedure (TP2) for the Suzuki Cross–Coupling Reaction

An oven-dry Schlenk tube, equipped with a stir bar was charged with the corresponding BODIPY **3a**–**3e** (1.0 equiv.), the appropriate (het)arylboronic acid (8.0 equiv.), K_3_PO_4_ (16.0 equiv.), and toluene (6.0 mL) under N_2_. The stirred solution was sparged with N_2_ for 5 min. A catalytic amount of Pd(OAc)_2_ (10.0 mol%) and SPhos (22.0 mol%) was added and the reaction mixture was heated to 90 °C. After completion of the reaction as judged by TLC analysis, the reaction mixture was diluted with water (5.0 mL) and extracted with ethyl acetate. The combined organic layers were washed with water and brine and dried over anhydrous MgSO_4_. The solvent was removed under reduced pressure and the reaction mixture was adsorbed on SiO_2_-gel and the solvent was evaporated on a rotary evaporator under vacuum. After flash-chromatography (SiO_2_-gel, EtOAc/hexanes gradient) purification, 8-aryl-2,3,5,6-tetra-(het)arylBODIPYs were obtained as highly colored solids.

The synthetic details to access each dye as well as the corresponding NMR spectra are collated in the Supplementary Materials.

### 3.2. Spectroscopic Measurements

The photophysical properties were registered in diluted solutions (around 2 × 10^−6^ M), prepared by adding the corresponding solvent to the residue from an adequate amount of a concentrated stock solution in acetone, after vacuum evaporation of this solvent. UV–Vis absorption and fluorescence spectra were recorded on a Varian model CARY 4E spectrophotometer and an Edinburgh Instruments spectrofluorometer (model FLSP 920), respectively. Fluorescence quantum yields (ϕ) were obtained using cresyl violet (ϕ = 0.54 in methanol) in the spectral window 600–650 nm, and a zinc phthalocyanine (ϕ = 0.30 in toluene with 1% of pyridine) in the 660–750 nm window, as references, from corrected spectra (detector sensitivity to the wavelength). The values were corrected by the refractive index of the solvent. Radiative decay curves were registered with the time correlated single-photon counting technique as implemented in the aforementioned spectrofluorometer. Fluorescent emission was monitored at the maximum emission wavelength after excitation by means of a Fianium pulsed laser (time resolution of picoseconds) with tunable wavelength. The fluorescence lifetime (τ) was obtained after the deconvolution of the instrumental response signal from the recorded decay curves by means of an iterative method. The goodness of the exponential fit was controlled by statistical parameters (Chi-square, Durbin–Watson and the analysis of the residuals). The radiative (k_fl_) and non-radiative (k_nr_) rate constants were calculated from the fluorescence quantum yield and lifetime; k_fl_ = ϕ/τ and k_nr_ = (1 − ϕ)/τ.

The photoinduced production of singlet oxygen (^1^O_2_) was determined by direct measurement of the luminescence at 1276 nm with a NIR detector integrated in the aforementioned spectrofluorometer (InGaAs detector, Hamamatsu G8605-23, Hamamatsu Photonics, Shizuoka, Japan). The ^1^O_2_ signal was registered in the front configuration (front face), 40° and 50° to the excitation and emission beams, respectively, and leaned 30° to the plane formed by the direction of incidence and registration in cells of 1 cm. The signal was filtered by a low cut-off of 850 nm.

### 3.3. Computational Calculations

Ground state geometries were optimized with the range-separated WB97XD hybrid functional, within the Density Functional Theory (DFT), using the triple valence basis set with a polarization and diffuse function (6-311 + g*), as implemented in the Gaussian 16. The geometries were considered as the energy minimum when the corresponding frequency analysis did not give any negative value. The absorption spectra were simulated with the Time Dependent method (TD) as vertical Franck–Condon transitions using the above functional and basis sets. All calculations were run in the “Arina” informatics cluster of the UPV/EHU.

### 3.4. Laser Experiments

Liquid solutions of dyes in ethyl acetate were contained in 1 cm optical-path rectangular quartz cells, carefully sealed to avoid solvent evaporation during experiments. The liquid solutions were transversely pumped at their absorption maxima (polyphenylBODIPYs at around 570–610 nm and polythiopheneBODIPYs at around 625–640 nm) with a wavelength tunable OPO coupled to the third harmonic (355 nm) of a *Q*-switched Nd:YAG laser (Lotis TII 2134) at a repetition rate of 1 Hz and energy of 40 mJ (see experimental set up in Scheme S1 in Supplementary Materials). The energy conversion efficiency of the OPO depends on the fixed wavelength, decreasing as the wavelength increases. Thus, the pumping energy from the OPO was around 9–11 mJ at 570–610 nm and 7–8 mJ at 625–640 nm. The excitation pulses were line-focused onto the cell using a combination of positive and negative cylindrical lenses (f = 15 cm and f = −15 cm, respectively) perpendicularly arranged to irradiate the full length of the cell and maximize the conversion efficiency from the pump laser to the emitted laser. The plane parallel oscillation cavity (2 cm length) consisted of a 90% reflective aluminum mirror acting as back reflector, and the lateral face of the cell acting as output coupler (4% reflectivity). The position of the rear mirror was finely aligned to get a homogenous and well-defined spot in the laser emission from the dye solution. The input pump and output energies were detected by an Ophir power meter. To discard the influence of amplified spontaneous emission (ASE) in the recorded output energy the aforementioned pumping energies were well above the laser threshold where the ASE (FWHM 40–50 nm) narrows into a sharp laser emission (FWHM around 5 nm) and a well-defined coherent laser spot was visualized at long distances (meters) from the cell.

The photostability of the dyes in ethyl acetate solution was evaluated using pumping energy and geometry exactly equal to that of the laser experiments. We used spectroscopic quartz cuvettes with 1 cm optical paths and depths *L* = 0.1 cm to allow for the minimum solution volume (*V*_S_ = 40 μL) to be excited. The lateral faces were grounded, whereupon no laser oscillation was obtained. Nevertheless, information about photostabilities can be obtained by monitoring the decrease in laser-induced fluorescence (LIF) intensity. In order to facilitate comparisons independently of the experimental conditions and sample, the photostability figure of merit was defined as the accumulated pump energy absorbed by the system (E_dose_), per mole of dye, before the output energy falls to a 90% of its initial value. In terms of experimental parameters, this energy dose, in units of GJ mol^−1^, can be expressed as:

$$E_{dose}^{90\%}(GJ\cdot mol^{-1})=\frac{E_{\mathrm{pump}}(GJ)\cdot \left(1-10^{-\varepsilon CL}\right)\cdot \sum_{\#\mathrm{pulses}}f}{CV_{S}}$$

where *E*_pump_ is the energy per pulse in GJules, *C* is the molar concentration, *ε* is the molar absorption coefficient in units of M^−1^ cm^−1^, *L* is the depth of the cuvette expressed in cm, *V*_S_ is the solution volume, in liters, within the cuvette, and *f* is the ratio between the LIF intensity after #pulses and the LIF intensity in the first pulse. It can be shown that Σ*f* accounts for the reduction in pump absorption due to species photo-degradation. To speed up the experiment the pump repetition rate was increased up to 10 Hz. The fluorescent emission was monitored perpendicular to the excitation beam, collected by an optical fiber, and imaged onto a spectrometer (USB2000+ from Ocean Optics, Orlando, FL, USA).

## 4. Conclusions

Chemoselective cross-coupling reactions on BODIPY enabled the initial exclusive functionalization of the *meso*-position via the Liebeskind cross-coupling reaction. A further quadruple Suzuki–Miyaura on the brominated positions was conducted to introduce (het)aryl fragments to yield a chart of dye lasers featuring bright and long-lasting fluorescence and laser emission in the red–NIR spectral region. The BODIPYs bearing peripheral phenyls displayed strong spectral bands within the red edge of the visible, whose efficiency can be modulated by the grafting of electron acceptor and mainly electron donor groups in the *para* position of the phenyl groups linked to the chromophoric pyrroles. Thus, laser efficiencies as high as 18% can be reached even after prolonged exposure to hard irradiation regimes (up to 14 GJ/mol can be endured to decrease the laser output just by 10%). In addition, thiophene is an appealing option to push the spectral bands beyond and reach the NIR region, while yielding unforeseen high laser efficiencies (up to 20% with an energy threshold to induce the aforementioned decrease up to 10 GJ/mol), in terms of the low fluorescence efficiencies recorded. Theoretical calculations sustain the ability of this functionalization to induce charge transfer character to the excited emitting state, enhancing the population inversion and the stimulated emission, in a similar way as occurs for styryl- or cyanine-based red dyes.

Therefore, the herein reported dyes embody a new generation of red–NIR radiation sources, which can efficiently contend with the currently available fluorophores in terms of efficiency and stability, but enable accessible and targetable functionalization, thanks to the rich chemistry of BODIPYs.

## Figures and Tables

**Table 1:** Multiple Suzuki–Miyaura cross-coupling reaction on **3a**–**e** ^1^.

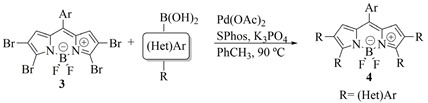					
Entry	Ar	R-B(OH)_2_	Reaction Time	Yield ^2^ (%)	Compound
1	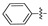	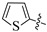	20 min	80	**4a**
2	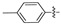	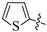	20 min	83	**4b**
3	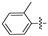	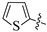	20 min	65	**4c**
4	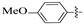	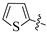	25 min	76	**4d**
5	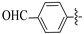	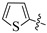	2 h	55	**4e**
6	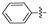	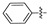	4 h	52	**4f**
7	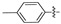	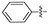	4 h	57	**4g**
8	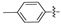	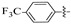	5 h	41	**4h**
9	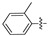	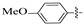	2 h	65	**4i**
10	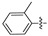	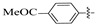	14 h	65	**4j**
11	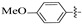	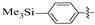	4 h	63	**4k**
12	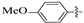	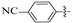	16 h	57	**4l**

^1^ Conditions: **3a** (1 equiv.), boronic acid (8 equiv.) Pd(OAc)_2_ (10 mol%), SPhos (22 mol%), K_3_PO_4_ (16 equiv.) in PhCH_3_ at 90 °C footnote. ^2^ Isolated yield.

**Table 2:** Photophysical (2 μM) and laser (0.5–0.75 mM) properties of the polyphenylBODIPYs in ethyl acetate. Full photophysical and laser data in more media are listed in Tables S2 and S3, respectively.

Dye	λ_ab_ ^1^ (nm)	ε_max_∙10^−4 2^ (M^−1^∙cm^−1^)	λ_fl_ ^3^ (nm)	ϕ ^4^	τ ^5^ (ns)	λ_la_ ^6^ (nm)	%Eff ^7^	E_dose_ ^8^ (GJ/mol)
**4f**	577.0	5.0	623.0	0.26	2.28	630.0	8.5	9.0
**4g**	579.0	5.1	618.0	0.32	2.92	628.0	9.8	9.4
**4h**	566.5	7.0	597.5	0.47	3.50	611.0	13.5	14.1
**4i**	615.0	3.9	653.0	0.78	4.12	670.0	6.4	5.9
**4j**	585.0	6.1	622.0	0.52	3.72	636.0	8.3	13.5
**4k**	584.5	4.0	617.0	0.83	5.37	625.0	17.8	2.3
**4l**	569.0	6.8	604.0	0.53	4.22	612.0	11.2	5.2

^1^ Absorption wavelength. ^2^ Molar absorption. ^3^ Fluorescence wavelength. ^4^ Fluorescence quantum yield. ^5^ Fluorescence lifetime. ^6^ Lasing wavelength. ^7^ Laser efficiency. ^8^ Photostability, defined as the amount of pumping energy absorbed by the dye to retain 90% of the laser induced emission.

**Table 3:** Photophysical (2 μM) properties, in solvents of different polarity; cyclohexane (c-hex), ethyl acetate (EtOAc) and acetonitrile (ACN), and laser (0.5–0.75 mM) properties in ethyl acetate of the polythiopheneBODIPYs. Full photophysical and laser data in more media are listed in Tables S4 and S5, respectively.

Dye	Solvent	λ_ab_ ^1^ (nm)	ε_max_∙10^−4 2^ (M^−1^∙cm^−1^)	λ_fl_ ^3^ (nm)	ϕ ^4^	τ ^5^ (ns)	λ_la_ ^6^ (nm)	%Eff ^7^	E_dose_ ^8^ (GJ/mol)
	c-hex	638.5	6.0	688.0	0.16	3.47			
**4a**	EtOAc	630.0	5.0	700.5	0.08	1.07	717.0	11.8	1.3
	ACN	624.5	4.1	711.5	0.03	0.42			
	c-hex	636.0	4.0	682.0	0.20	3.83			
**4b**	EtOAc	628.0	3.4	695.0	0.09	1.23	712.0	11.4	6.0
	ACN	621.5	3.0	707.0	0.03	0.50			
	c-hex	639.0	5.3	685.5	0.21	3.85			
**4c**	EtOAc	632.5	4.6	696.0	0.11	1.32	710.0	9.3	7.6
	ACN	627.0	4.1	705.5	0.04	0.55			
	c-hex	629.0	6.5	678.5	0.39	4.10			
**4d**	EtOAc	625.0	5.1	690.0	0.15	1.41	705.0	20.5	10.0
	ACN	619.0	4.2	703.5	0.05	0.62			
	c-hex	650.0	4.8	708.0	0.17	1.65			
**4e**	EtOAc	639.0	4.1	721.0	0.04	0.41	744.0	8.7	7.6
	ACN	634.5	3.3	736.0	0.02	0.41			

^1^ Absorption wavelength. ^2^ Molar absorption. ^3^ Fluorescent wavelength. ^4^ Fluorescence quantum yield. ^5^ Fluorescence lifetime. ^6^ Lasing wavelength. ^7^ Laser efficiency. ^8^ Photostability, defined as the amount of pumping energy absorbed by the dye to retain 90% of the laser induced emission.

## Data Availability

The data presented in this study are available in the article or Supplementary Materials.

## Supplementary Materials

The following supporting information can be downloaded at: https://www.mdpi.com/article/10.3390/molecules28124750/s1, Synthesis and characterization details (including NMR spectra and Table S1) of all compounds, Table S1: Optimization of the multiple Suzuki–Miyaura cross-coupling reaction on **3a**; Tables S2 and S4: Photophysical data, Tables S3 and S5: Laser data; Figures S1 and S2: Absorption and fluorescence spectra, Scheme S1: Experimental set up for the laser measurements. References [44,45] are cited in the supplementary materials.

## Sample Availability

Samples of all compounds are available from the authors.
