# Supplementary material for: Extended BODIPYs as Red–NIR Laser Radiation Sources with Emission from 610 nm to 750 nm
Source: Molecules. 2023 Jun 13;28(12):4750. doi: 10.3390/molecules28124750 (PMC10305162; doi:10.3390/molecules28124750)
Supplement: Supplementary file 1 [file molecules-28-04750-s001.zip › molecules-2431713-supplementary.pdf]

Extended BODIPYs as Red–NIR Laser Radiation Sources with Emission from 610 nm to 750 nm

Ainhoa Oliden-Sánchez [1], Enrique Alvarado-Martínez [2], Diana E. Ramírez-Ornelas [2], Miguel A. Vázquez [2], Edurne Avellanal-Zaballa [1], Jorge Bañuelos [1,*] and Eduardo Peña-Cabrera [2,*]

[1] Departamento de Química Física, Universidad del País Vasco (UPV/EHU), Barrio Sarriena s/n, Aptado 644, 48940 Leioa, Bizkaia, Spain; ainhoa.oliden@ehu.es (A.O.-S.); edurne.avellanal@ehu.es (E.A.-Z.)

[2] Departamento de Química, Universidad de Guanajuato, Noria Alta s/n, Guanajuato 36050, Guanajuato, Mexico; e.alvaradomartinez@ugto.mx (E.A.-M.); ramirez.de@ugto.mx (D.E.R.-O.); mvazquez@ugto.mx (M.A.V.)

* Correspondence: jorge.banuelos@ehu.es (J.B.); eduardop@ugto.mx (E.P.-C.)

Table of contents

**1. Synthesis and characterization (Table S1)**	S2
**2. References**	S12
**3. Photophysical data (Tables S2-S5)**	S13
**4. Absorption and fluorescence spectra (Figures S1-S2)**	S17
**5. Laser set up (Scheme S1)**	S18
**6. ^{1}H NMR and ^{13}C NMR spectra**	S19

1. Synthesis and characterization

Synthesis of 2,3,5,6 tetrabromo-8-methylthioBODIPY 2 [S1].

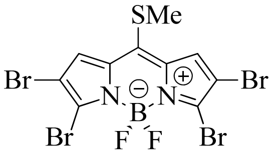

2

A round-bottom flask equipped with a stir bar, 8-methylthioBODIPY **1** (50.0 mg, 0.2100 mmol, 1.0 equiv.) was dissolved in acetic acid (7.0 mL) and NBS (187.0 mg, 1.0501 mmol, 5.0 equiv.) was added to the solution, and the reaction mixture was stirred at room temperature for 12 h. After TLC showed that the reaction went to completion, the mixture was poured into water (50.0 mL) and the pH was adjusted using saturated Na_2CO_3 to pH 7. The product was extracted with ethyl acetate, washed with brine, dried over anhyd. $MgSO_4$, and filtered. The solvents were removed under reduced pressure. The reaction mixture was adsorbed on SiO_2-gel and the solvent was evaporated on a rotary evaporator under vacuum. After flash-chromatography (SiO_2-gel, EtOAc/hexanes gradient) purification, 8-methylthio-2,3,5,6-tetra-bromoBODIPY was obtained as a red solid (58.0 mg, 50%); ^{1}H NMR (500 MHz, $CDCl_3$): δ 7.45 (s, 2H), 2.83 (s, 3H).

Synthesis of 8-aryl-2,3,5,6-tetrabromoBODIPYs 3a-3e.

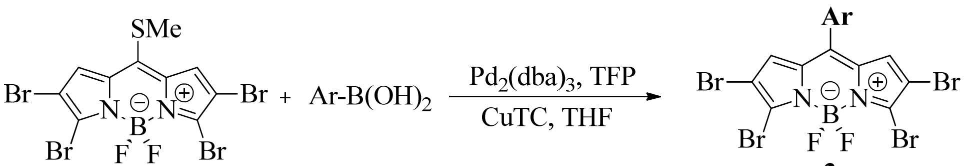

2 **3**

Synthesis of BODIPY 3a [S2].

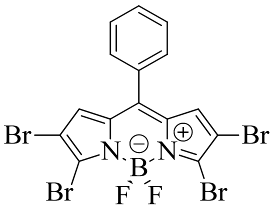

3a

According to TP1. **2** (55.4 mg, 0.1 mmol, 1.0 equiv), phenylboronic acid (36.6 mg, 0.3 mmol, 3.0 equiv), CuTC (57.2 mg, 0.3 mmol, 3.0 equiv), $Pd_2(dba)_3$ (2.3 mg, 2.5 x 10^{-3} mmol, 2.5 mol%), and tri-2-furylphosphine (1.8 mg, 7.5 x 10^{-3} mmol, 7.5 mol%) for 2 h were reacted. Flash chromatography on silica gel afforded the desired product **3a** as a red solid (10.5 mg, 18% yield); ^{1}H NMR (500 MHz,

$CDCl_3$): δ 7.65-7.47 (m, 5H), 6.90 (s, 2H); ^{13}C NMR (126 MHz, $CDCl_3$): δ 142.9, 135.4, 135.0, 131.9, 131.7, 131.6, 130.5, 129.1, 112.2.

Synthesis of BODIPY 3b [S3].

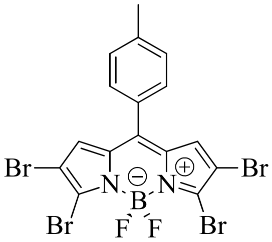

3b

According to TP1. **2** (55.4 mg, 0.1 mmol, 1.0 equiv), *p*-tolylboronic acid (40.8 mg, 0.3 mmol, 3.0 equiv), CuTC (57.2 mg, 0.3 mmol, 3.0 equiv), $Pd_2(dba)_3$ (2.3 mg, 2.5 x 10^{-3} mmol, 2.5 mol%), and tri-2-furylphosphine (1.8 mg, 7.5 x 10^{-3} mmol, 7.5 mol%) for 2 h were reacted. Flash chromatography on silica gel afforded the desired product **3b** as a purple solid (28.7 mg, 48% yield); ^{1}H NMR (500 MHz, $CDCl_3$): δ 7.38-7.32 (m, 4H), 6.82 (s, 2H), 2.46 (s, 3H); ^{13}C NMR (126 MHz, $CDCl_3$): δ 142.6, 134.9, 134.8, 131.7, 130.7, 130.6, 129.8, 129.1, 112.0, 21.7.

Synthesis of BODIPY 3c [1].

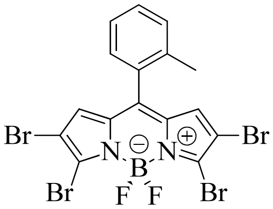

3c

According to TP1. **2** (55.4 mg, 0.1 mmol, 1.0 equiv), *o*-tolylboronic acid (40.8 mg, 0.3 mmol, 3.0 equiv), CuTC (57.2 mg, 0.3 mmol, 3.0 equiv), $Pd_2(dba)_3$ (2.3 mg, 2.5 x 10^{-3} mmol, 2.5 mol%), and tri-2-furylphosphine (1.8 mg, 7.5 x 10^{-3} mmol, 7.5 mol%) for 1 h were reacted. Flash chromatography on silica gel afforded the desired product **3c** as a purple solid (45.4 mg, 76% yield); ^{1}H NMR (500 MHz, $CDCl_3$): δ 7.45 (td, J = 7.6, J =1.3 Hz, 1H), 7.34-7.29 (m, 2H), 7.21 (dd, J = 7.6, J = 1.0 Hz, 1H), 6.68 (s, 2H), 2.23 (s, 3H); ^{13}C NMR (126 MHz, $CDCl_3$): δ 142.5, 136.6, 135.7, 135.4, 131.1, 131.0, 130.9, 130.6, 129.9, 125.9, 112.3, 20.2.

Synthesis of BODIPY 3d [S2].

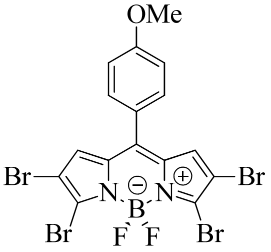

3d

According to TP1. **2** (55.4 mg, 0.1 mmol, 1.0 equiv), *p*-methoxyphenylboronic acid (45.6 mg, 0.3 mmol, 3.0 equiv), CuTC (57.2 mg, 0.3 mmol, 3.0 equiv), $Pd_2(dba)_3$ (2.3 mg, 2.5 x 10^{-3} mmol, 2.5 mol%), and tri-2-furylphosphine (1.8 mg, 7.5 x 10^{-3} mmol, 7.5 mol%) for 40 min were reacted. Flash chromatography on silica gel afforded the desired product **3d** as a purple solid (38.7 mg, 63% yield); ^1H NMR (500 MHz, $CDCl_3$): δ 7.46 (d, *J* = 8.8 Hz, 2H), 7.06 (d, *J* = 8.8 Hz, 2H), 6.95 (s, 2H), 3.92 (s, 3H); ^{13}C NMR (126 MHz, $CDCl_3$): δ 161.7, 142.0, 133.7, 133.1, 131.3, 130.4, 123.1, 113.6, 110.7, 54.6.

Synthesis of BODIPY 3e [S1].

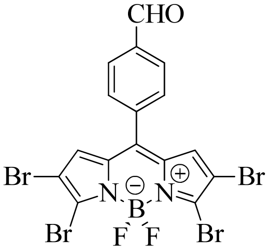

3e

According to TP1. **2** (55.4 mg, 0.1 mmol, 1.0 equiv), *p*-formylphenylboronic acid (45.0 mg, 0.3 mmol, 3.0 equiv), CuTC (57.2 mg, 0.3 mmol, 3.0 equiv), $Pd_2(dba)_3$ (2.3 mg, 2.5 x 10^{-3} mmol, 2.5 mol%), and tri-2-furylphosphine (1.8 mg, 7.5 x 10^{-3} mmol, 7.5 mol%) for 1 h were reacted. Flash chromatography on silica gel afforded the desired product **3e** as a purple solid (23.1 mg, 38% yield); ^1H NMR (500 MHz, $CDCl_3$): δ 10.15 (s, 1H), 8.06 (d, *J* = 8.0 Hz, 2H), 7.66 (d, *J* = 8.0 Hz, 2H), 6.85 (s, 2H); ^{13}C NMR (126 MHz, $CDCl_3$): δ 191.0, 140.5, 138.2, 137.3, 136.6, 134.7, 131.3, 131.0, 130.0, 112.8.

Synthesis of the 8-aryl-2,3,5,6-tetra-(het)arylBODIPYs 4a-4l.

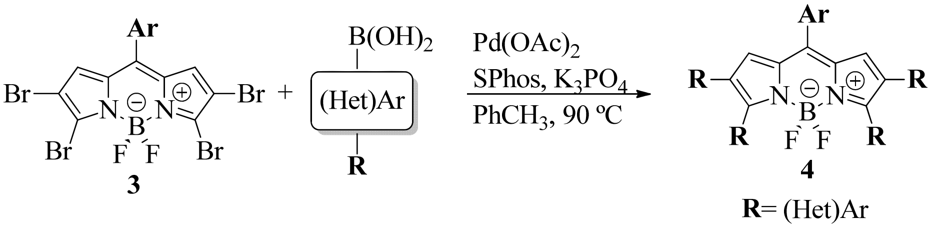

Synthesis of BODIPY 4a [S4].

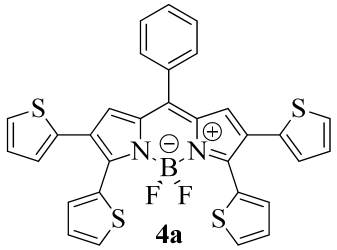

4a

According to TP2. **3a** (58.4 mg, 0.1 mmol, 1.0 equiv.), 2-thienylboronic acid (102.4 mg, 0.8 mmol, 8.0 equiv.), $Pd(OAc)_2$ (2.2 mg, 10 x 10^{-3} mmol, 10.0 mol%), SPhos (9.0 mg, 22.0 x 10^{-3} mmol, 22.0 mol%), and K_3PO_4 (339.6 mg, 1.6 mmol, 16.0 equiv.) for 20 min were reacted. Flash chromatography on silica gel afforded the desired product **4a** as a dark green solid (47.7 mg, 80% yield); ^{1}H NMR (500 MHz, $CDCl_3$): δ 7.64-7.55 (m, 7H), 7.50 (d, *J* = 5.0 Hz, 2H), 7.20 (d, *J* = 4.6 Hz, 2H), 7.10 (dd, *J* = 5.0 Hz, *J* = 3.7 Hz, 2H), 6.95 (s, 2H), 6.92 (dd, *J* = 5.1 Hz, *J* = 3.6 Hz, 2H), 6.76 (d, *J* = 3.1 Hz, 2H); ^{13}C NMR (126 MHz, $CDCl_3$): δ 149.1, 143.4, 135.2, 135.1, 133.9, 132.3 (t, *J* = 3.7 Hz), 130.9, 130.6, 130.5, 129.7,129.0 (dd, *J* = 2.3 Hz, *J* = 4.6 Hz), 128.6, 128.1, 127.2 (d, *J* = 2.5 Hz), 126.3, 125.6.

Synthesis of BODIPY 4b [S5].

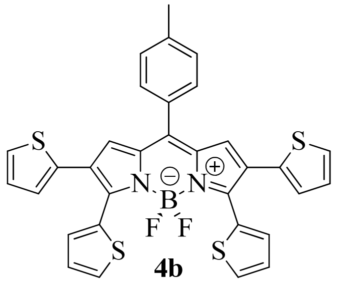

4b

According to TP2. **3b** (59.7 mg, 0.1 mmol, 1.0 equiv.), 2-thienylboronic acid (102.4 mg, 0.8 mmol, 8.0 equiv), $Pd(OAc)_2$ (2.2 mg, 10 x 10^{-3} mmol, 10.0 mol%), SPhos (9.0 mg, 22.0 x 10^{-3} mmol, 22.0 mol%), and K_3PO_4 (339.6 mg, 1.6 mmol, 16.0 equiv.) for 20 min were reacted. Flash chromatography on silica gel afforded the desired product **4b** as a dark green solid (50.7 mg, 83% yield); ^{1}H NMR (500 MHz, $CDCl_3$): δ 7.55-7.53 (m, 4H), 7.49 (dd, *J* = 5.1 Hz, *J* = 1.1 Hz, 2H), 7.38 (d, *J* = 7.8 Hz, 2H), 7.20 (dd, *J* = 5.1 Hz, *J* = 1.1 Hz, 2H), 7.10 (dd, *J* = 5.0 Hz, *J* = 3.7 Hz, 2H), 6.98 (s, 2H), 6.91 (dd, *J* = 5.1 Hz, 3.7 Hz, 2H), 6.76 (dd, *J* = 3.6 Hz, *J* = 1.1 Hz, 2H), 2.51 (s, 3H); ^{13}C NMR (126 MHz, $CDCl_3$): δ 148.9, 144.0, 141.2, 135.4, 135.2, 132.4 (t, *J* = 3.4 Hz), 131.2, 131.1, 130.8, 129.7, 129.5, 129.1 (t, *J* = 4.3 Hz), 128.2, 127.4, 126.4, 125.6, 21.7.

Synthesis of BODIPY 4c.

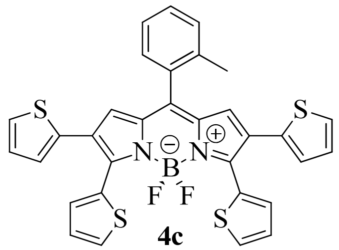

4c

According to TP2. **3c** (59.7 mg, 0.1 mmol, 1.0 equiv.), 2-thienylboronic acid (102.4 mg, 0.8 mmol, 8.0 equiv.), $Pd(OAc)_2$ (2.2 mg, 10 x 10^{-3} mmol, 10.0 mol%), SPhos (9.0 mg, 22.0 x 10^{-3} mmol, 22.0 mol%), and K_3PO_4 (339.6 mg, 1.6 mmol, 16.0 equiv.) for 20 min were reacted. Flash chromatography on silica gel afforded the desired product **4c** as a dark green solid (39.6 mg, 65% yield); TLC (15% EtOAc/hexanes, R_f = 0.4); mp > 260 °C; IR (KBr, cm^{-1}): 3103 (w), 2918 (w), 1546 (s), 1429 (m), 1351 (w), 1273 (w), 1235 (s), 1226 (s), 1210 (s), 1177 (s), 1119 (m), 987 (m), 888 (w), 836 (m), 820 (m); ^1H NMR (500 MHz, $CDCl_3$): δ 7.56 (dd, *J* = 3.6 Hz, *J* = 0.8 Hz, 2H), 7.51 (dd, *J* = 5.0 Hz, *J* = 1.1 Hz, 2H), 7.47 (td, *J* = 7.5 Hz, *J* = 1.7 Hz, 2H), 7.40-7.34 (m, 3H), 7.19 (dd, *J* = 5.1 Hz, *J* = 1.1 Hz, 2H), 7.11 (dd, *J* = 5.0 Hz, *J* = 3.7 Hz, 1H), 6.90 (dd, *J* = 5.1 Hz, *J* = 3.6 Hz, 2H), 6.74 (dd, *J* = 3.6 Hz, *J* = 1.1 Hz, 2H), 6.72 (s, 2H), 2.38 (s, 3H); ^{13}C NMR (126 MHz, $CDCl_3$): δ 149.4, 143.0, 136.9, 135.6, 135.3, 133.2, 132.5 (t, *J* = 3.6 Hz), 131.1, 130.7, 130.3, 129.9, 129.8, 129.2 (dd, *J* = 4.5 Hz, *J* = 2.2 Hz), 127.5, 127.4, 127.3, 126.5, 125.7, 125.6, 20.5; HRMS (ESI^+) m/z calcd for $C_{32}H_{22}BF_2N_2S_4$ $[M + H]^+$ 611.0682, found 611.0690.

Synthesis of BODIPY 4d.

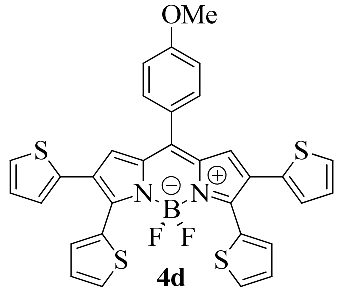

4d

According to TP2. **3d** (61.4 mg, 0.1 mmol, 1.0 equiv.), 2-thienylboronic acid (102.4 mg, 0.8 mmol, 8.0 equiv.), $Pd(OAc)_2$ (2.2 mg, 10 x 10^{-3} mmol, 10.0 mol%), SPhos (9.0 mg, 22.0 x 10^{-3} mmol, 22.0 mol%), and K_3PO_4 (339.6 mg, 1.6 mmol, 16.0 equiv.) for 25 min were reacted. Flash chromatography on silica gel afforded the desired product **4d** as a dark green solid (47.6 mg, 76% yield); TLC (30% EtOAc/hexanes, R_f = 0.6); mp > 260 °C; IR (KBr, cm^{-1}): 2920 (w), 1712 (w), 1603 (m), 1573 (w), 1541 (s), 1453 (m), 1395 (m), 1298 (w), 1238 (s), 1176 (s) 1089 (s), 1075 (s), 987 (m), 852 (w), 769 (w), 697 (m); ^1H NMR (500 MHz, $CDCl_3$): δ 7.62-7.59 (m, 2H), 7.52 (dd, *J* = 3.6 Hz, *J* = 0.8 Hz, 2H), 7.49 (dd, *J* = 5.1 Hz,

J = 1.1 Hz, 2H), 7.20 (dd, J = 5.1 Hz, J = 1.1 Hz, 2H), 7.11-7.09 (m, 2H), 7.00 (s, 2H), 6.92 (dd, J = 5.1 Hz, J = 3.6 Hz, 2H), 6.76 (dd, J = 3.6 Hz, J = 1.1 Hz, 2H), 3.94 (s, 3H); ^{13}C NMR (126 MHz, $CDCl_3$): δ 162.0, 148.6, 143.8, 135.5, 135.1, 132.5, 132.3 (t, J = 3.6 Hz), 131.2, 129.7, 129.0 (t, J = 2.6 Hz), 128.1, 127.4 (d, J = 1.8 Hz), 126.5, 126.3, 125.6, 114.3, 55.7; HRMS (ESI$^+$) m/z calcd for $C_{32}H_{21}BF_2N_2OS_4K$ [M + K]$^+$ 665.0235, found 665.0250.

Synthesis of BODIPY 4e.

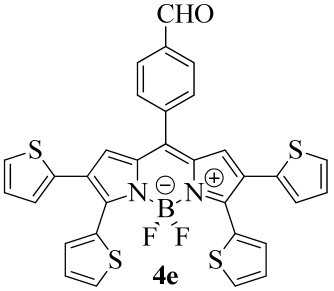

4e

According to TP2. **3e** (61.7 mg, 0.1 mmol, 1.0 equiv.), 2-thienylboronic acid (102.4 mg, 0.8 mmol, 8.0 equiv.), $Pd(OAc)_2$ (2.2 mg, 10 x 10^{-3} mmol, 10.0 mol%), SPhos (9.0 mg, 22.0 x 10^{-3} mmol, 22.0 mol%), and K_3PO_4 (339.6 mg, 1.6 mmol, 16.0 equiv.) for 2 h were reacted. Flash chromatography on silica gel afforded the desired product **4e** as a dark green solid (34.4 mg, 55% yield); TLC (40% EtOAc/hexanes, R_f = 0.6); mp > 260 °C; IR (KBr, cm^{-1}): 3103 (w), 2918 (w), 1546 (s), 1429 (m), 1351 (w), 1273 (w), 1235 (s), 1226 (s), 1210 (s), 1177 (s), 1119 (m), 987 (m), 888 (w), 836 (m), 820 (m); ^{1}H NMR (500 MHz, $CDCl_3$): δ 10.17 (s, 1H), 8.10 (d, J = 8.3 Hz, 2H), 7.81 (d, J = 8.1 Hz, 2H), 7.57 (dd, J = 3.6 Hz, J = 0.9 Hz, 2H), 7.52 (dd, J = 5.0 Hz, J = 1.2 Hz, 2H), 7.22 (dd, J = 5.1 Hz, J = 1.1 Hz, 2H), 7.11 (dd, J = 5.0 Hz, J = 3.7 Hz, 2H), 6.92 (dd, J = 5.1 Hz, J = 3.6 Hz, 2H), 6.87 (s, 2H), 6.76 (dd, J = 3.6 Hz, J = 1.1 Hz, 2H); ^{13}C NMR (126 MHz, $CDCl_3$): δ 191.5, 150.1, 141.0, 139.8, 137.6, 135.0, 134.9, 132.7 (t, 3.7 Hz), 131.3, 130.9, 130.2, 129.8, 129.7, 127.7, 127.5 (d, J = 3.9 Hz), 126.7, 126.0; HRMS (ESI$^+$) m/z calcd for $C_{32}H_{19}BF_2N_2OS_4K$ [M + K]$^+$ 663.0079, found 663.0095.

Synthesis of BODIPY 4f [S2].

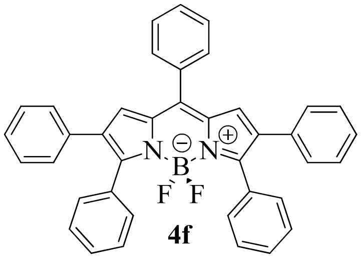

4f

According to TP2. **3a** (58.4 mg, 0.1 mmol, 1.0 equiv.), phenylboronic acid (97.5 mg, 0.8 mmol, 8.0 equiv.), $Pd(OAc)_2$ (2.2 mg, 10 x 10^{-3} mmol, 10.0 mol%), SPhos (9.0 mg, 22.0 x 10^{-3} mmol, 22.0 mol%),

and K_3PO_4 (339.6 mg, 1.6 mmol, 16.0 equiv.) for 4 h were reacted. Flash chromatography on silica gel afforded the desired product **4f** as a purple solid (29.8 mg, 52% yield); ^{1}H NMR (500 MHz, $CDCl_3$): δ 7.72-7.68 (m, 2H), 7.64-7.55 (m, 3H), 7.48 (d, J = 6.9 Hz, 4H), 7.38-7.29 (m, 6H), 7.17-7.13 (m, 6H), 7.03-6.98 (m, 6H); ^{13}C NMR (126 MHz, $CDCl_3$): δ 156.4, 143.8, 134.6, 134.3, 133.7, 131.6, 130.6, 130.3, 129.0, 128.5, 128.3, 128.2, 128.0, 127.7, 126.8, 125.4, 116.8.

Synthesis of BODIPY 4g [S3].

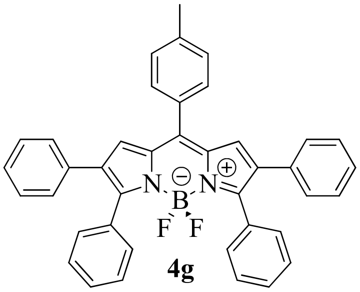

4g

According to TP2. **3b** (59.8 mg, 0.1 mmol, 1.0 equiv.), phenylboronic acid (97.5 mg, 0.8 mmol, 8.0 equiv.), $Pd(OAc)_2$ (2.2 mg, 10 x 10^{-3} mmol, 10.0 mol%), SPhos (9.0 mg, 22.0 x 10^{-3} mmol, 22.0 mol%), and K_3PO_4 (339.6 mg, 1.6 mmol, 16.0 equiv.) for 4 h were reacted. Flash chromatography on silica gel afforded the desired product **4g** as a purple solid (33.4 mg, 57% yield); ^{1}H RMN (400 MHz, $CDCl_3$): δ 7.59 (d, J = 7.93 Hz, 2 H), 7.45-7.49 (m, 4 H), 7.38 (d, J = 7.93 Hz, 2 H), 7.29-7.35 (m, 4 H), 7.13-7.17 (m, 6 H), 7.04 (s, 2 H), 6.98-7.02 (m, 4 H), 2.51 (s, 3 H); ^{13}C RMN (101 MHz, $CDCl_3$): δ 156.4, 144.5, 141.0, 134.8, 134.6, 134.0, 131.9, 131.7, 130.9, 130.5, 129.3, 129.2, 128.8, 128.4, 128.3, 128.0, 127.0, 21.6.

Synthesis of BODIPY 4h.

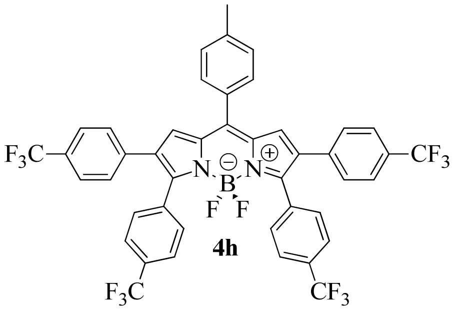

4h

According to TP2. **3b** (59.8 mg, 0.1 mmol, 1.0 equiv.), *p*-(trifluoromethyl)phenyl boronic acid (151.9 mg, 0.8 mmol, 8.0 equiv.), $Pd(OAc)_2$ (2.2 mg, 10 x 10^{-3} mmol, 10.0 mol%), SPhos (9.0 mg, 22.0 x 10^{-3} mmol, 22.0 mol%), and K_3PO_4 (339.6 mg, 1.6 mmol, 16.0 equiv.) for 5 h were reacted. Flash chromatography on silica gel afforded the desired product **4h** as a purple solid (35.2 mg, 41% yield); TLC (5% EtOAc/hexanes, R_f = 0.5); mp > 260 °C; IR (KBr, cm^{-1}): 2927 (w), 2852 (w), 2640 (w), 1618 (m), 1573 (s), 1563 (s), 1444 (w), 1411 (w), 1327 (s), 1275 (m), 1232 (s), 1214 (s), 1159 (s), 1117 (s), 1058 (s),

1014 (s), 999 (m), 843 (m), 834 (m), 697 (w); ^{1}H NMR (500 MHz, $CDCl_3$): δ 7.63-7.57 (m, 10H), 7.45-7.42 (m, 6H), 7.14 (s, 2H), 7.10 (d, *J* = 8.1 Hz, 4 H); ^{13}C NMR (126 MHz, $CDCl_3$): δ 154.9, 146.8, 142.0, 135.2, 134.8, 133.6, 131.6, 131.4, 131.1, 131.0, 130.9 (d, *J* = 4.8 Hz), 130.0, 129.7, 129.6, 129.4, 128.6, 125.6 (d, *J* = 3.4 Hz), 125.3 (d, *J* = 3.4 Hz), 125.2, 125.1, 123.1, 122.9, 21.7; HRMS (ESI$^+$) m/z calcd for $C_{44}H_{26}BF_{14}N_2$ $[M + H]^+$ 859.1921, found 859.1910.

Synthesis of BODIPY 4i

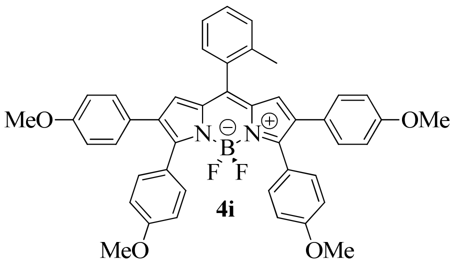

4i

According to TP2. **3c** (59.8 mg, 0.1 mmol, 1.0 equiv.), *p*-methoxyphenylboronic acid (121.6 mg, 0.8 mmol, 8.0 equiv.), $Pd(OAc)_2$ (2.2 mg, 10 x 10^{-3} mmol, 10.0 mol%), SPhos (9.0 mg, 22.0 x 10^{-3} mmol, 22.0 mol%), and K_3PO_4 (339.6 mg, 1.6 mmol, 16.0 equiv.) for 2 h were reacted. Flash chromatography on silica gel afforded the desired product **4i** as a purple solid (46.0 mg, 65% yield); TLC (60% EtOAc/hexanes, R_f = 0.6); mp > 260 °C; IR (KBr, cm^{-1}): 3015 (m), 1602 (s), 1542 (s), 1411 (s), 1388 (s), 1259 (s), 1120 (s), 1077 (s); ^{1}H NMR (500 MHz, $CDCl_3$): δ 7.46-7.41 (m, 6H), 7.37-7.32 (m, 2H), 6.92 (d, *J* = 8.8 Hz, 4H), 6.85 (d, *J* = 8.8 Hz, 4H), 6.69 (d, *J* = 8.8 Hz, 4H), 6.66 (s, 2H), 3.81 (s, 6H), 3.74 (s, 6H), 2.40 (s, 3H); ^{13}C NMR (126 MHz, $CDCl_3$): δ 160.3, 158.7, 156.2, 141.7, 137.1, 135.0, 134.3, 134.0, 132.2, 130.5, 129.7, 129.4, 127.1, 126.8, 125.5, 124.4, 113.7, 113.6, 55.3, 55.3, 20.5; HRMS (ESI$^+$) m/z calcd for $C_{44}H_{37}BF_2N_2O_4K$ $[M + K]^+$ 745.2453, found 745.2460.

Synthesis of BODIPY 4j.

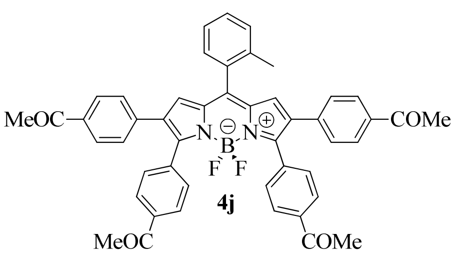

4j

According to TP2. **3c** (59.8 mg, 0.1 mmol, 1.0 equiv.), *p*-acetylphenylboronic acid (131.2 mg, 0.8 mmol, 8.0 equiv.), $Pd(OAc)_2$ (2.2 mg, 10 x 10^{-3} mmol, 10.0 mol%), SPhos (9.0 mg, 22.0 x 10^{-3} mmol, 22.0 mol%), and K_3PO_4 (339.6 mg, 1.6 mmol, 16.0 equiv.) for 14 h were reacted. Flash chromatography on silica gel afforded the desired product **4j** as a purple solid (34.0 mg, 45% yield); TLC (60% EtOAc/hexanes, R_f = 0.4); mp > 260 °C; IR (KBr, cm^{-1}): 2921 (w), 1552 (s), 1498 (m), 1402 (w), 1324 (s), 1251 (m), 1331 (s),

1145 (s), 1019 (m), 932 (m), 880 (m); ^{1}H NMR (500 MHz, $CDCl_3$): δ 7.92 (d, *J* = 8.5 Hz, 4H), 7.74 (d, *J* = 8.5 Hz, 4H), 7.59 (d, *J* = 8.3 Hz, 4H), 7.52 (td, *J* = 7.5 Hz, *J* = 1.5 Hz, 1H), 7.46-7.39 (m, 3H), 7.06 (d, *J* = 8.5 Hz, 4H), 6.90 (s, 2H), 2.60 (s, 6H), 2.52 (s, 6H), 2.43 (s, 3H); ^{13}C NMR (126 MHz, $CDCl_3$): δ 197.8, 197.6, 155.9, 145.6, 138.0, 137.6, 136.8, 136.0, 135.9, 135.8, 134.1, 133.0, 130.9, 130.8, 130.3, 130.2, 128.9, 128.9, 128.6, 128.5, 128.2, 125.8, 26.8, 26.7, 20.6; HRMS (ESI$^+$) m/z calcd for $C_{48}H_{38}BF_2N_2O_4$ [M + H]$^+$ 755.2848, found 755.2838.

Synthesis of BODIPY 4k.

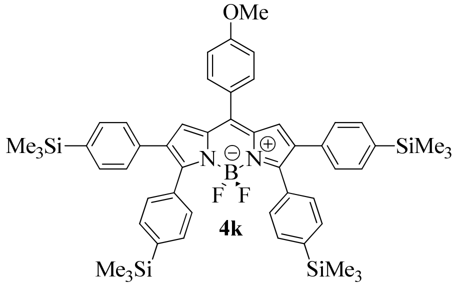

4k

According to TP2. **3d** (61.4 mg, 0.1 mmol, 1.0 equiv.), *p*-(trimethylsilyl)phenylboronic acid (155.3 mg, 0.8 mmol, 8.0 equiv.), $Pd(OAc)_2$ (2.2 mg, 10 x 10^{-3} mmol, 10.0 mol%), SPhos (9.0 mg, 22.0 x 10^{-3} mmol, 22.0 mol%), and K_3PO_4 (339.6 mg, 1.6 mmol, 16.0 equiv.) for 4 h were reacted. Flash chromatography on silica gel afforded the desired product **4k** as a purple solid (56.0 mg, 63% yield); TLC (5% EtOAc/hexanes, R_f = 0.5); mp > 260 °C; IR (KBr, cm^{-1}): 2966 (w), 1562 (s), 1488 (m), 1402 (w), 1324 (s), 1261 (m), 1131 (s), 1045 (s), 1009 (m), 982 (m), 850 (m); ^{1}H NMR (500 MHz, $CDCl_3$): δ 7.63 (d, *J* = 8.7 Hz, 2H), 7.46 (s, 8H), 7.30 (d, *J* = 8.1 Hz, 4H), 7.07 (d, *J* = 8.7 Hz, 2H), 7.06 (s, 2H), 6.98 (d, *J* = 8.1 Hz, 4H), 3.93 (s, 3H), 0.26 (s, 18H), 0.21 (s, 18H); ^{13}C NMR (126 MHz, $CDCl_3$): δ 161.8, 156.3, 143.9, 141.6, 139.0, 134.9, 134.6, 133.3, 132.9, 132.7, 132.3, 129.5, 128.9, 127.6, 127.0, 114.1, 55.7, -0.1, -1.00; HRMS (ESI$^+$) m/z calcd for $C_{52}H_{61}BF_2N_2OSi_4K$ [M + K]$^+$ 929.3563, found 929.3577.

Synthesis of BODIPY 4l.

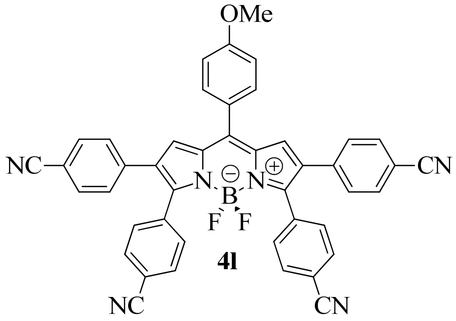

4l

According to TP2. **3d** (61.4 mg, 0.1 mmol, 1.0 equiv.), *p*-cyanophenylboronic acid (117.6 mg, 0.8 mmol, 8.0 equiv.), $Pd(OAc)_2$ (2.2 mg, 10 x 10^{-3} mmol, 10.0 mol%), SPhos (9.0 mg, 22.0 x 10^{-3} mmol, 22.0 mol%),

and K_3PO_4 (339.6 mg, 1.6 mmol, 16.0 equiv.) for 16 h were reacted. Flash chromatography on silica gel afforded the desired product **4l** as a purple solid (40.0 mg, 57% yield); TLC (5% EtOAc/hexanes, R_f = 0.5); mp > 260 °C; ^{1}H NMR (500 MHz, $CDCl_3$): δ 7.67-7.64 (m, 6H), 7.54 (d, *J* = 8.0 Hz, 4H), 7.50 (d, *J* = 8.1 Hz, 4H), 7.19 (s, 2H), 7.15 (d, *J* = 8.4 Hz, 2H), 7.09 (d, *J* = 8.1 Hz, 4H), 3.96 (s, 3H); ^{13}C NMR (126 MHz, $CDCl_3$): δ 162.9, 153.9, 147.4, 137.7, 135.5, 135.4, 133.1, 132.8, 132.5, 132.2, 131.1, 130.2, 128.9, 125.9, 118.6, 118.4, 114.8, 113.7, 111.4, 55.8; HRMS (ESI$^+$) m/z calcd for $C_{44}H_{25}BF_2N_2OK$ [M + K]$^+$ 741.1790, found 741.1805.

Table S1. Optimization of the multiple Suzuki-Miyaura cross-coupling reaction on **3a**.[1]

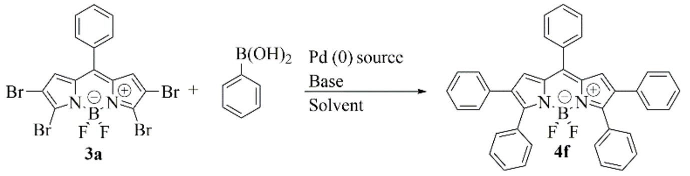

Entry	Boronic acid (equiv.)	Pd (0) source	Base	Solvent	Temp (ºC)	Yield [2]
1	4.7	$Pd(PPh_3)_4$	Na_2CO_3 [3] (1M)	PhMe	110	- [4]
2	8	$Pd(PPh_3)_4$	Na_2CO_3 (16 equiv.)	PhMe/THF/ H_2O (1:1:1)	80	40%
3	8	$Pd(OAc)_2$ SPhos [5]	K_3PO_4 (16 equiv.)	CH_3Ph	90	52%

[1] Conditions: **3a** (1 equiv), Pd (0) source (10 mol%), 4 h. [2] Isolated yield. [3] Aq. Na_2CO_3 (1 mL / 1 M). [4] Incomplete reaction and multiple products. [5] SPhos (22 mol%).

2. References

[S1] Belmonte-Vázquez, J.L.; Avellanal-Zaballa, E.; Enríquez-Palacios, E.; Cerdán, L.; Esnal, I.; Bañuelos, J.; Villegas-Gómez, C.; López Arbeloa, I.; Peña-Cabrera, E. Synthetic Approach to Readily Accessible Benzofuran-Fused Borondipyrromethenes as Red-Emitting Laser Dyes. *J. Org. Chem.* **2019**, *84*, 2523–2541. DOI: 10.1021/acs.joc.8b02933

[S2] Jiao, J.; Pang, W.; Zhou, J.; Wei, Y.; Mu, X.; Bai, G.; Hao, E. Regioselective Stepwise Bromination of Boron Dipyrromethene (BODIPY) Dyes. *J. Org. Chem.* **2011**, *76*, 9988– 9996. DOI: 10.1021/jo201754m

[S3] Lakshmi, V.; Ravikanth, M. Synthesis of Hexasubstituted Boron-Dipyrromethenes Having a Different Combination of Substituents. *Eur. J. Org. Chem.* **2014**, *2014*, 5757– 5766. DOI: 10.1002/ejoc.201402599

[S4] Feng, Z.; Jiao, L.; Feng, Y.; Yu, C.; Chen, N.; Wei, Y.; Mu, X.; Hao, E. Regioselective and Stepwise Syntheses of Functionalized BODIPY Dyes through Palladium-Catalyzed Cross-Coupling Reactions and Direct C–H Arylations. *J. Org. Chem*. **2016**, *81*, 6281-6291. DOI: 10.1021/acs.joc.6b00858

[S5] Liu, P.; Gao, F.; Zhou, L.; Chen, Y.; Chen, Z. Tetrathienyl-functionalized red- and NIR-absorbing BODIPY dyes appending various peripheral substituents**.** *Org. Biomol. Chem*. **2017**, *15*, 1393-1399. DOI: 10.1039/C6OB02612E

3. Photophysical data

Table S2. Photophysical properties of the polyphenylBODIPYs in diluted solutions (2 μM).

	λ_{ab} (nm)	$\varepsilon_{max}\cdot10^{-4}$ ($M^{-1}\cdot cm^{-1}$)	λ_{fl} (nm)	$\Delta\nu_{St}$ (cm^{-1})	ϕ	τ (ns)	$k_{fl}\cdot10^{-8}$ (s^{-1})	$k_{nr}\cdot10^{-8}$ (s^{-1})
**4f**								
c-hex	590.0	6.9	621.0	845	0.30	2.66	1.10	2.66
EtOAc	582.0	5.8	618.0	1000	0.33	2.69	1.23	2.48
ACN	577.0	5.0	623.0	1280	0.26	2.28	1.39	3.00
**4g**								
c-hex	586.0	5.6	618.0	885	0.35	3.25	1.07	1.20
EtOAc	579.0	5.1	618.0	1090	0.32	2.92	1.08	2.34
ACN	574.0	4.8	617.0	1215	0.26	2.29	1.11	3.24
**4h**								
c-hex	575.0	8.0	600.5	740	0.52	4.15	1.26	1.15
EtOAc	566.5	7.0	597.5	915	0.47	3.50	1.35	1.50
ACN	562.0	5.5	598.5	1085	0.40	2.91	1.36	2.07
**4i**								
c-hex	621.5	4.8	649.5	695	0.96	5.20	1.86	0.07
EtOAc	615.0	3.9	653.0	945	0.78	4.12	1.89	0.53
ACN	608.0	3.6	659.5	1285	0.39	2.22	1.77	2.73
**4j**								
c-hex	593.0	6.7	623.0	815	0.43	3.54	1.22	1.60
EtOAc	585.0	6.1	622.0	1015	0.52	3.72	1.40	1.28
ACN	578.5	5.6	624.5	1275	0.40	3.00	1.33	1.99
**4k**								
Et_2O*	586.0	5.5	618.0	885	0.88	5.47	1.60	0.22
EtOAc	584.5	4.0	617.0	900	0.83	5.37	1.55	0.31
ACN	579.5	2.7	614.5	985	0.87	5.28	1.65	0.23
**4l**								
Et_2O*	571.0	7.1	602.5	915	0.74	4.78	1.56	0.53
EtOAc	569.0	6.8	604.0	1020	0.53	4.22	1.25	1.11
ACN	565.0	5.1	603.0	1115	0.48	3.70	1.29	1.40

c-hex: cyclohexane; EtOAc: ethyl acetate; ACN: acetonitrile

*not soluble in cyclohexane, Et_2O: diethylether

absorption (λ_{ab}) and fluorescence (λ_{fl}) wavelength; Stokes shift ($\Delta\nu_{St}$); molar absorption (ε_{max}); fluorescence quantum yield (ϕ) and lifetime (τ); radiative (k_{fl}) and non-radiative (k_{nr}) rate constants

Table S3. Laser properties of the polyphenylBODIPYs in concentrated solutions of ethyl acetate.

C (mM)	λ_{la} (nm)	%Eff	E_{dose} (GJ/mol)	C (mM)	λ_{la} (nm)	%Eff	E_{dose} (GJ/mol)
**4g**				**4j**			
0.10	-	-		0.10	629	4.7	
0.25	625	5.1		0.25	631	5.9	
0.50	628	9.8	9.4	0.50	635	7.5	
0.75	630	6.5		0.75	636	8.3	13.5
1.00	633	3.3		1.00	638	5.1	
**4h**				**4k**			
0.10	-	-		0.10	624	10.0	
0.25	609	11.0		0.25	627	10.9	
0.50	611	13.5	14.2	0.50	625	17.8	2.3
0.75	613	12.8		0.75	623	12.6	
1.00	617	8.8		1.00	621	10.9	
**4i**				**4l**			
0.10	-	-		0.10	607	9.2	
0.25	-	-		0.25	612	10.0	
0.50	670	6.4	5.9	0.50	609	11.2	5.2
0.75	670	4.3		0.75	610	10.7	
1.00	672	4.3		1.00	608	8.4	

Lasing wavelength (λ_{la}) and efficiency (%Eff). Photostability (E_{dose}) was calculated as the amount of pumping energy absorbed by the dye to retain 90% of the laser induced emission at the concentration that optimizes the laser efficiency in each dye.

Table S4. Photophysical properties of the polythiopheneBODIPYs in diluted solutions (2 μM).

	λ_{ab} (nm)	$\varepsilon_{max} \cdot 10^{-4}$ ($M^{-1} \cdot cm^{-1}$)	λ_{fl} (nm)	$\Delta\nu_{St}$ (cm^{-1})	ϕ	τ (ns)	$k_{fl} \cdot 10^{-8}$ (s^{-1})	$k_{nr} \cdot 10^{-8}$ (s^{-1})
**4a**								
c-hex	638.5	6.0	688.0	1125	0.16	3.47	0.46	2.41
EtOAc	630.0	5.0	700.5	1600	0.08	1.07	0.72	8.62
ACN	624.5	4.1	711.5	1960	0.03	0.42	0.59	22.9
**4b**								
c-hex	636.0	4.0	682.0	1060	0.20	3.83	0.53	2.07
EtOAc	628.0	3.4	695.0	1535	0.09	1.23	0.77	7.34
ACN	621.5	3.0	707.0	1945	0.03	0.50	0.61	19.3
**4c**								
c-hex	639.0	5.3	685.5	1060	0.21	3.85	0.54	2.05
EtOAc	632.5	4.6	696.0	1445	0.11	1.32	0.81	6.73
ACN	627.0	4.1	705.5	1775	0.04	0.55	0.65	17.5
**4d**								
c-hex	629.0	6.5	678.5	1160	0.39	4.10	0.97	1.47
EtOAc	625.0	5.1	690.0	1510	0.15	1.41	1.07	5.97
ACN	619.0	4.2	703.5	1940	0.05	0.62	0.83	15.2
**4e**								
c-hex	650.0	4.8	708.0	1260	0.17	1.65	1.03	5.02
EtOAc	639.0	4.1	721.0	1780	0.04	0.41	0.87	23.5
ACN	634.5	3.3	736.0	2175	0.02	0.41	0.36	23.9

c-hex: cyclohexane; EtOAc: ethyl acetate; ACN: acetonitrile

absorption (λ_{ab}) and fluorescence (λ_{fl}) wavelength; Stokes shift ($\Delta\nu_{St}$); molar absorption (ε_{max}); fluorescence quantum yield (ϕ) and lifetime (τ); radiative (k_{fl}) and non-radiative (k_{nr}) rate constants

Table S5. Laser properties of the polythiopheneBODIPYs in concentrated solutions of ethyl acetate

C (mM)	λ_{la} (nm)	%Eff	E_{dose} (GJ/mol)	C (mM)	λ_{la} (nm)	%Eff	E_{dose} (GJ/mol)
**4a**				**4d**			
0.25	710	4.6		0.25	704	4.2	
0.50	715	10.1		0.50	704	17.7	
0.75	717	11.8	1.3	0.75	705	20.5	10.0
1.00	721	9.5		1.00	710	16.0	
**4b**				**4e**			
0.25	707	6.9		0.25	-	-	
0.50	709	11.4	5.9	0.50	744	4.8	
0.75	712	11.4		0.75	744	8.7	7.6
1.00	714	11.1		1.00	747	8.2	
**4c**							
0.25	705	7.0					
0.50	710	9.3	7.6				
0.75	713	8.4					
1.00	721	6.5					

lasing wavelength (λ_{la}) and efficiency (%Eff). Photostability (E_{dose}) was calculated as the amount of pumping energy absorbed by the dye to retain 90% of the laser induced emission at the concentration that optimizes the laser efficiency in each dye.

4. Absorption and fluorescence spectra

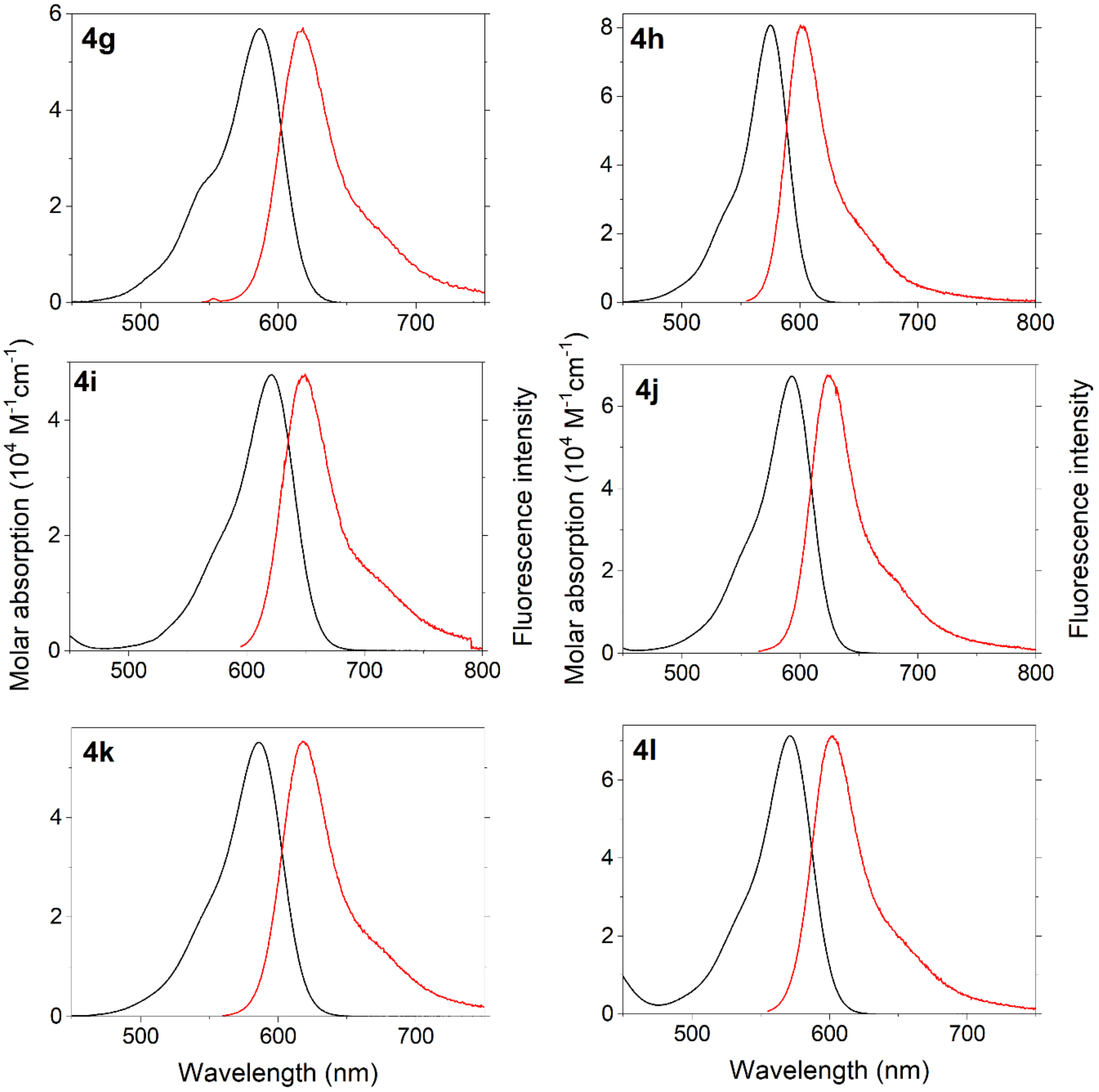

Figure S1. Absorption and normalized fluorescence (red) spectra of polyarylated BODIPYs in diluted solutions of cyclohexane (except for **4l** and **4k**, which are in diethyleter owing to solubility reasons).

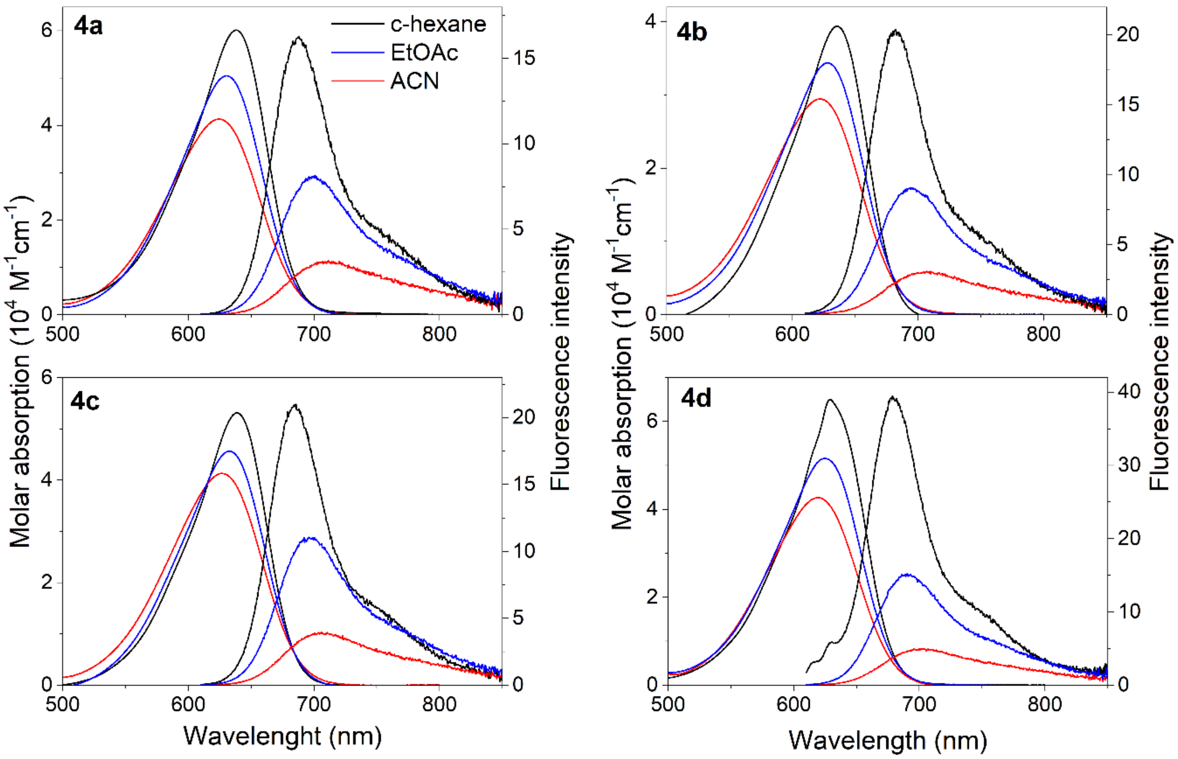

Figure S2. Absorption and fluorescence spectra of polythiopheneBODIPYs in diluted solutions of solvents of different polarity.

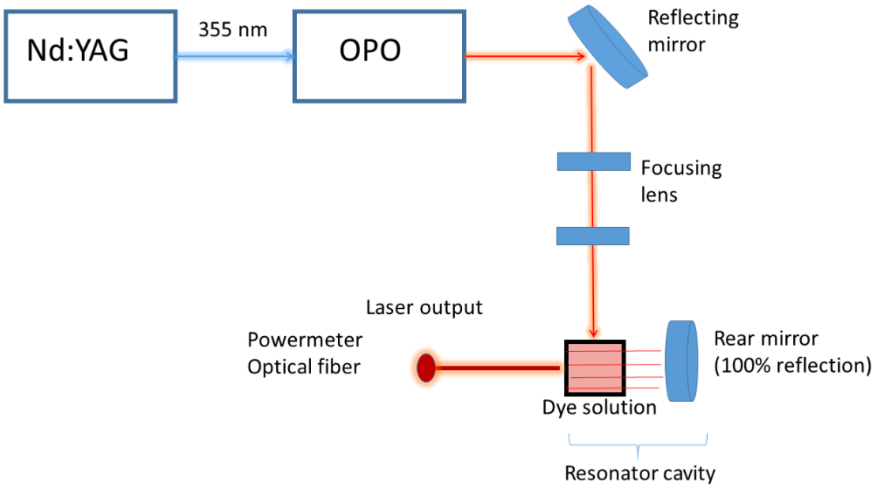

Scheme S1. Experimental set up for the laser measurements

6. ^{1}H NMR and ^{13}C NMR spectra

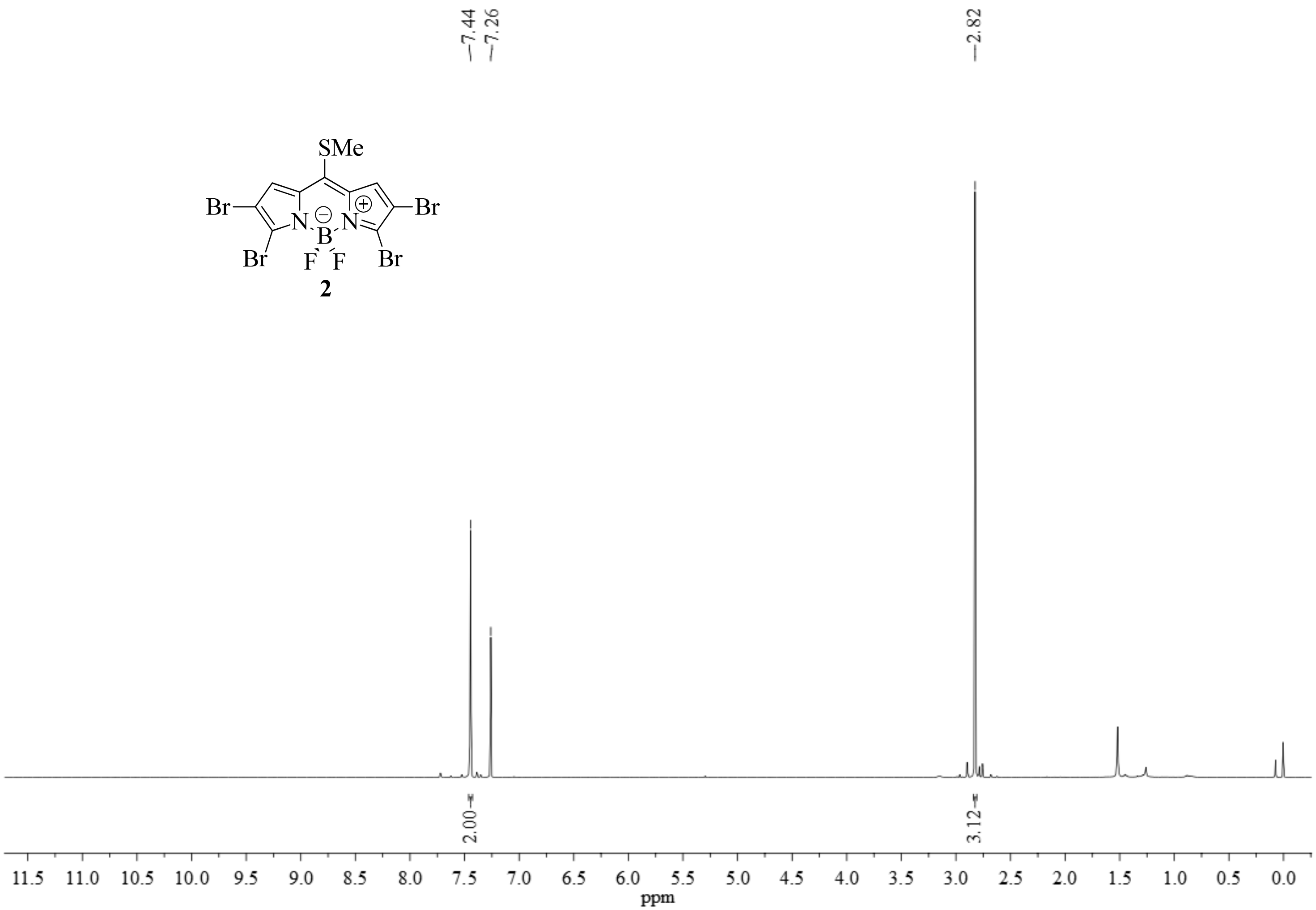

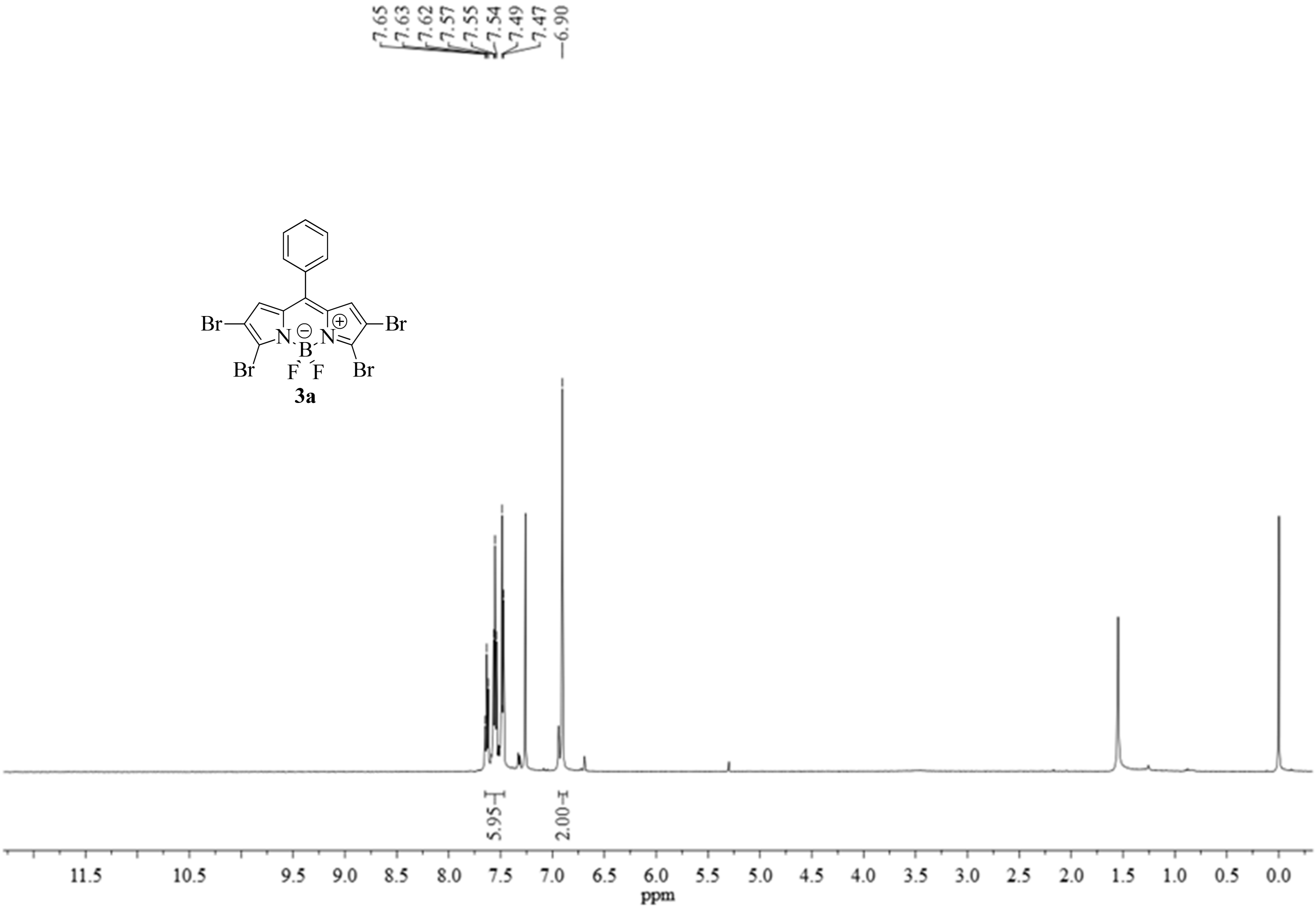
7.65
7.63
7.62
7.57
7.55
7.54
7.49
7.47
6.90
Br
Br
Br
Br
N
B
N
F
F
3a
5.95
2.00
11.5
10.5
9.5
9.0
8.5
8.0
7.5
7.0
6.5
6.0
5.5
5.0
4.5
4.0
3.5
3.0
2.5
2.0
1.5
1.0
0.5
0.0
ppm

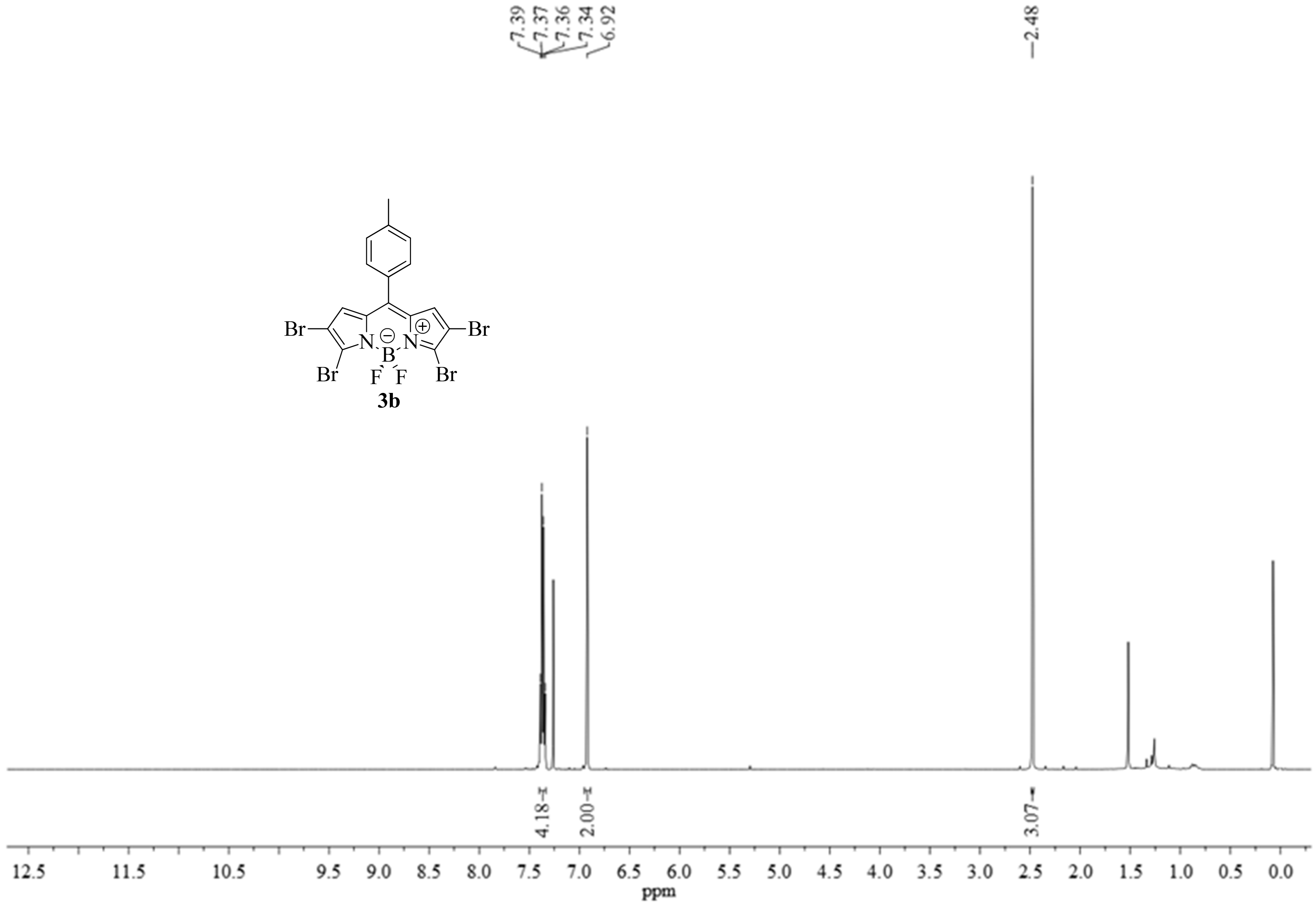
7.39
7.37
7.36
7.34
6.92
2.48
Br
Br
N
B
N
Br
Br
F
F
3b
4.18
2.00
3.07
12.5
11.5
10.5
9.5
9.0
8.5
8.0
7.5
7.0
6.5
6.0
5.5
5.0
4.5
4.0
3.5
3.0
2.5
2.0
1.5
1.0
0.5
0.0
ppm

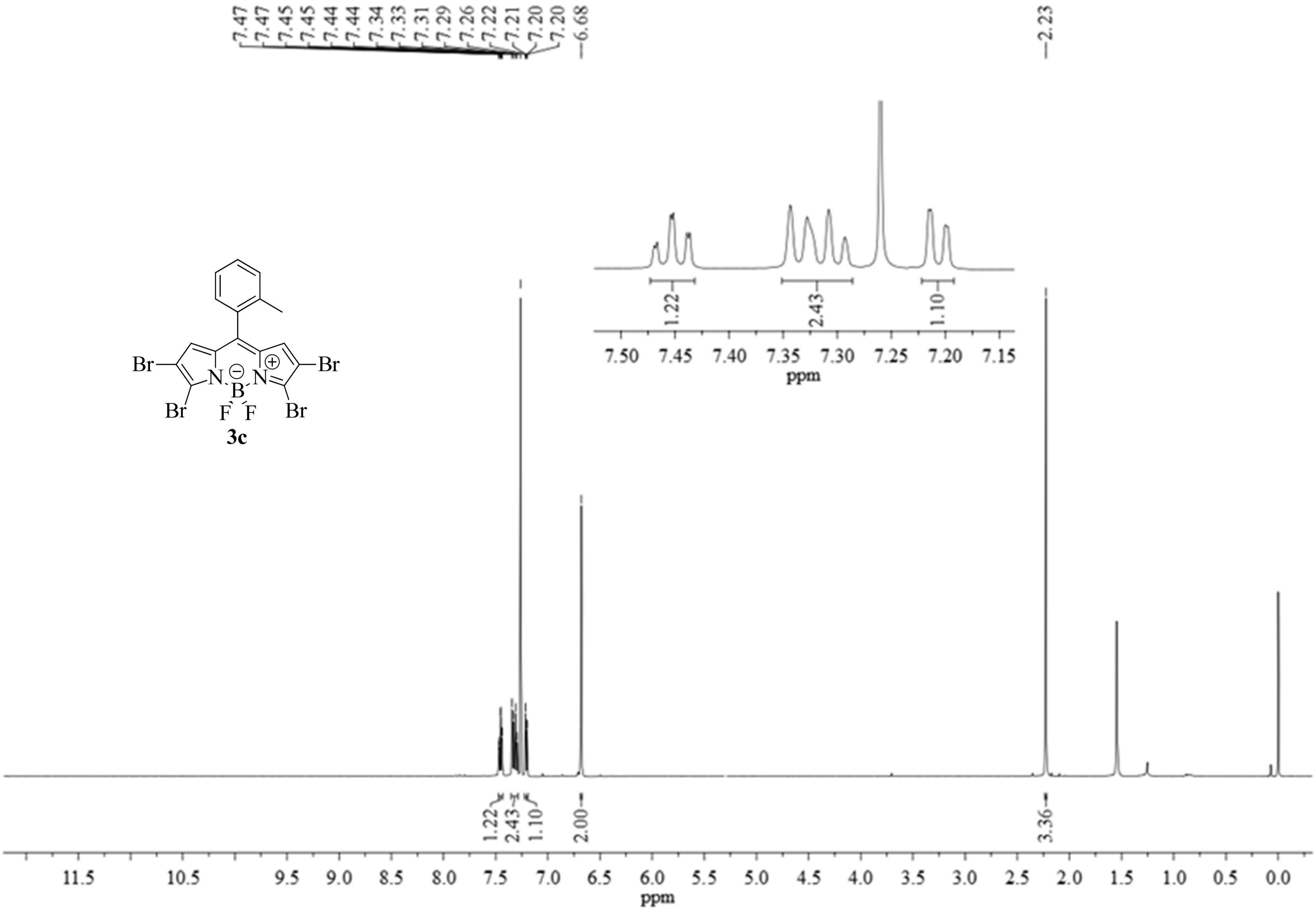
7.47
7.47
7.45
7.45
7.44
7.44
7.34
7.33
7.31
7.29
7.26
7.22
7.21
7.20
7.20
6.68
2.23
Br
Br
Br
Br
N
B
N
F
F
3c
1.22
2.43
1.10
7.50 7.45 7.40 7.35 7.30 7.25 7.20 7.15
ppm
1.22
2.43
1.10
2.00
3.36
11.5 10.5 9.5 9.0 8.5 8.0 7.5 7.0 6.5 6.0 5.5 5.0 4.5 4.0 3.5 3.0 2.5 2.0 1.5 1.0 0.5 0.0
ppm

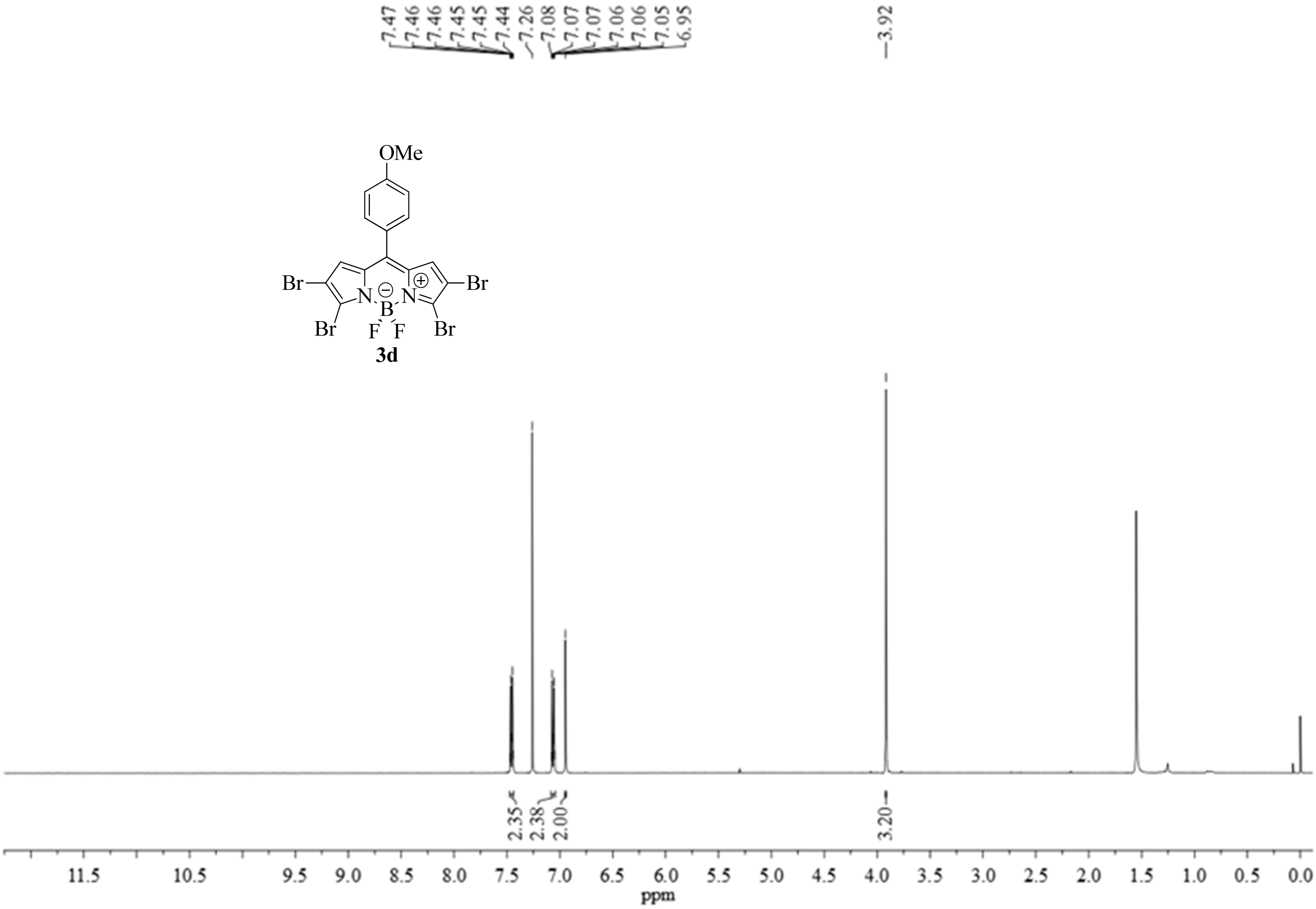

7.47
7.46
7.46
7.45
7.45
7.44
7.26
7.08
7.07
7.07
7.06
7.06
7.05
6.95
3.92
OMe
Br
Br
N
B
N
Br
Br
F
F
3d
2.35
2.38
2.00
3.20
11.5
10.5
9.5
9.0
8.5
8.0
7.5
7.0
6.5
6.0
5.5
5.0
4.5
4.0
3.5
3.0
2.5
2.0
1.5
1.0
0.5
0.0
ppm

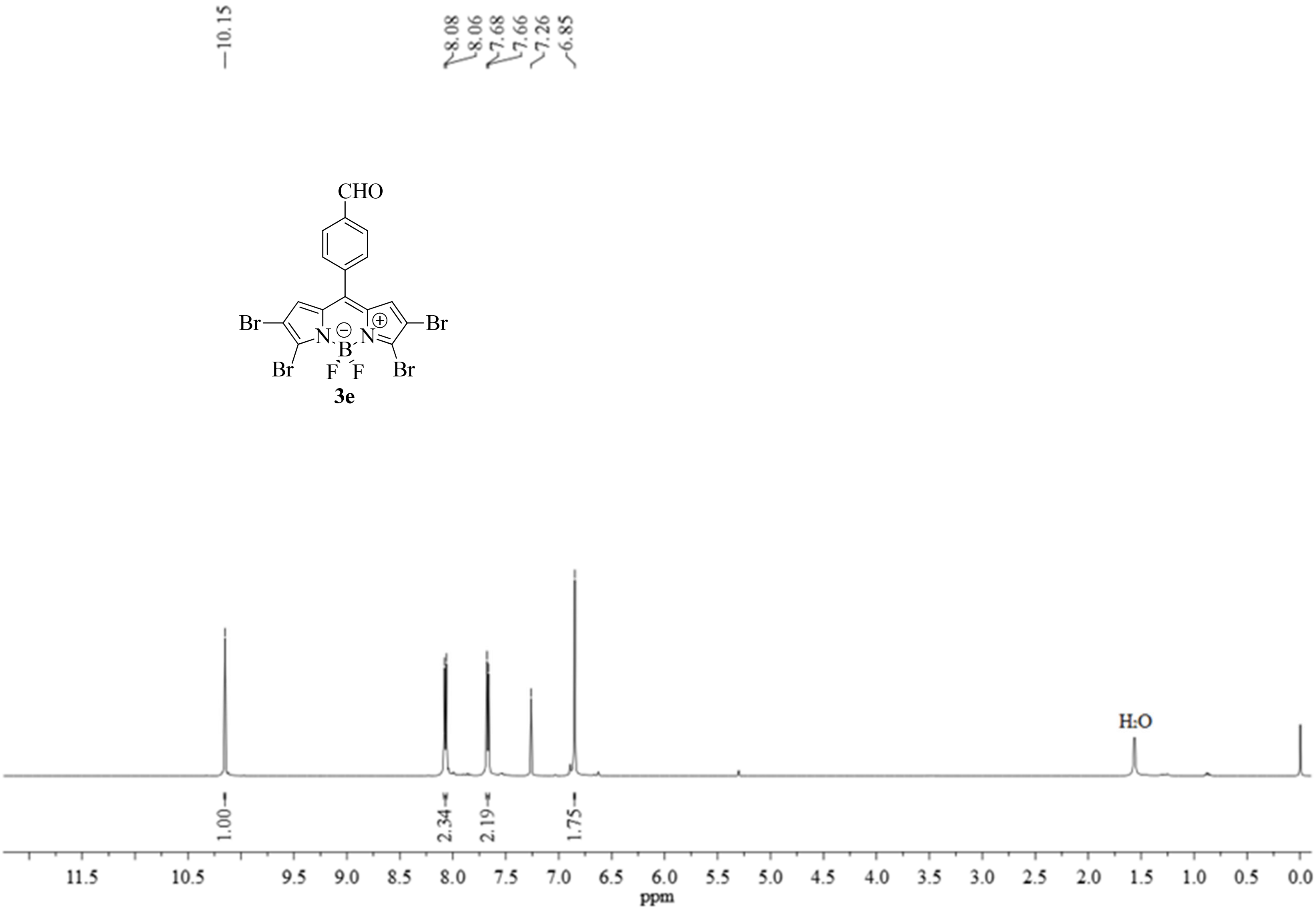
10.15
8.08
8.06
7.68
7.66
7.26
6.85
CHO
Br
N
B
F
H:O
1.00
2.34
2.19
1.75
11.5
10.5
9.5
9.0
8.5
8.0
7.5
7.0
6.5
6.0
5.5
5.0
4.5
4.0
3.5
3.0
2.5
2.0
1.5
1.0
0.5
0.0
ppm

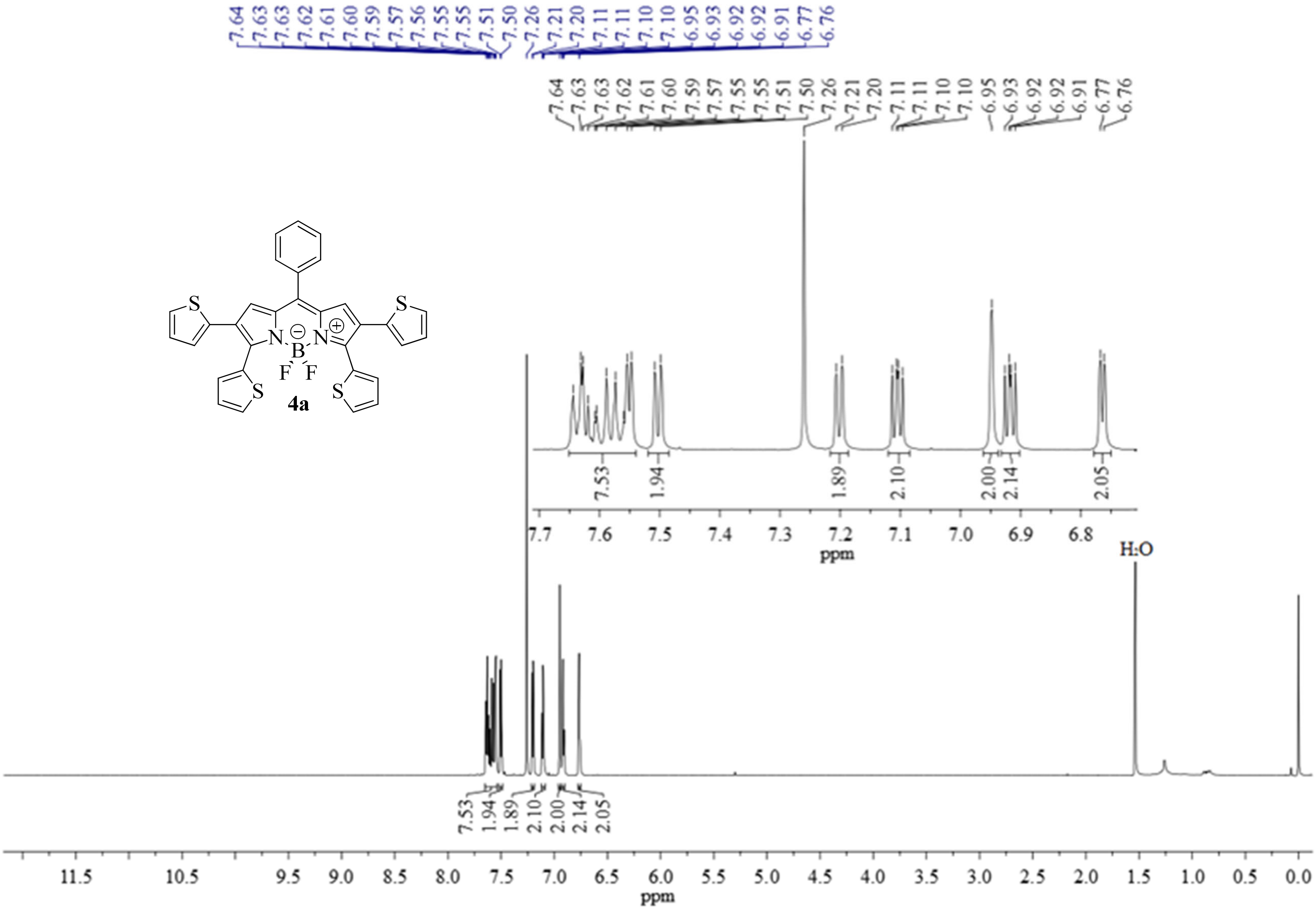

7.64 7.63 7.63 7.62 7.61 7.60 7.59 7.57 7.56 7.55 7.55 7.51 7.50 7.26 7.21 7.20 7.11 7.11 7.10 7.10 6.95 6.93 6.92 6.92 6.91 6.77 6.76
4a
7.53 1.94 1.89 2.10 2.00 2.14 2.05
ppm
H2O

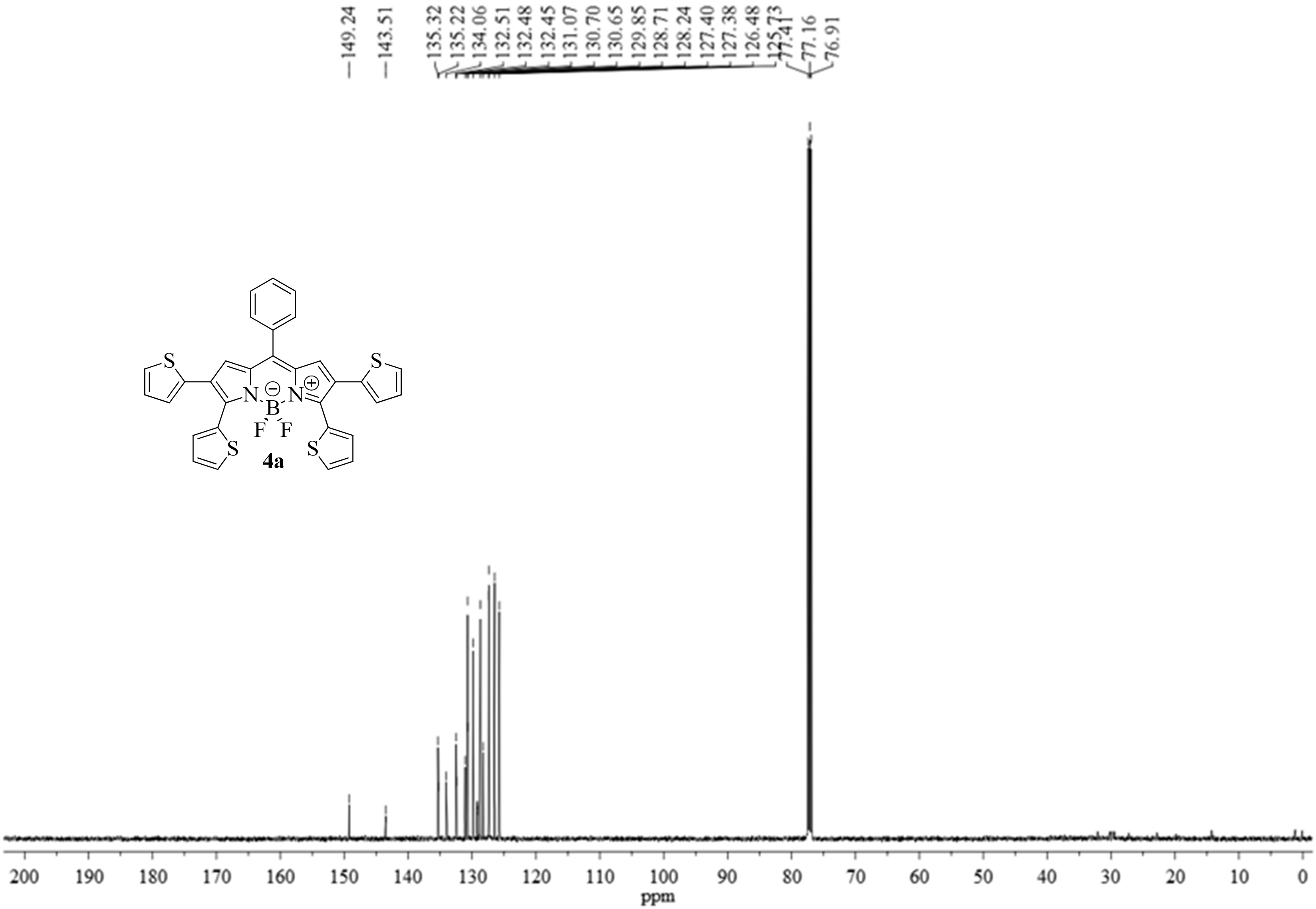
149.24
143.51
135.32
135.22
134.06
132.51
132.48
132.45
131.07
130.70
130.65
129.85
128.71
128.24
127.40
127.38
126.48
125.73
77.41
77.16
76.91
4a
ppm

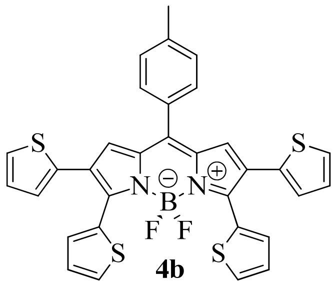

4b

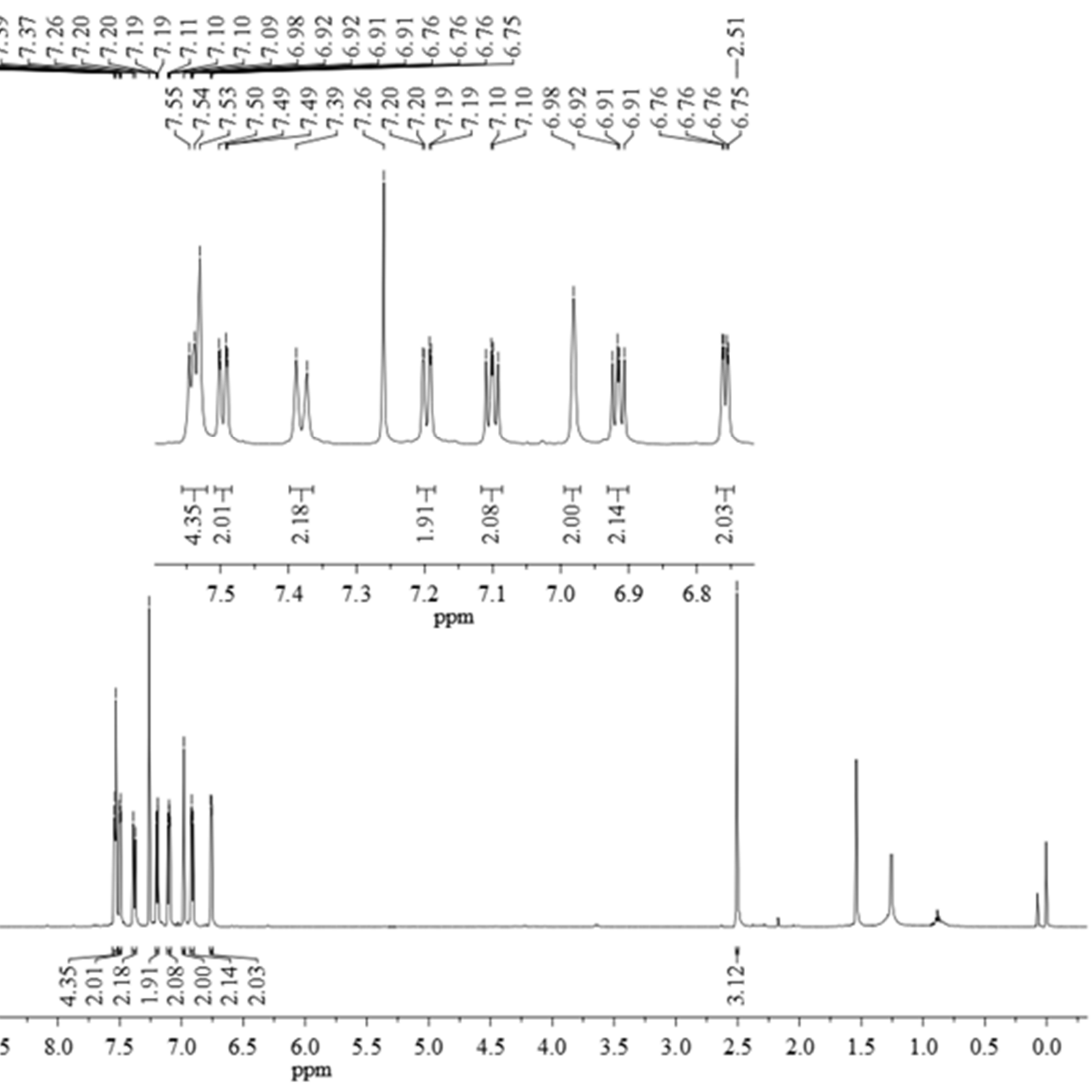
7.55 7.54 7.53 7.50 7.50 7.49 7.49 7.39 7.37 7.26 7.20 7.20 7.19 7.19 7.11 7.10 7.10 7.09 6.98 6.92 6.92 6.91 6.91 6.76 6.76 6.76 6.75
2.51
7.55 7.54 7.53 7.50 7.49 7.49 7.39 7.26 7.20 7.20 7.19 7.19 7.10 7.10 6.98 6.92 6.91 6.91 6.76 6.76 6.76 6.75
4.35 2.01 2.18 1.91 2.08 2.00 2.14 2.03
7.5 7.4 7.3 7.2 7.1 7.0 6.9 6.8
ppm
4.35 2.01 2.18 1.91 2.08 2.00 2.14 2.03
3.12
11.5 10.5 9.5 9.0 8.5 8.0 7.5 7.0 6.5 6.0 5.5 5.0 4.5 4.0 3.5 3.0 2.5 2.0 1.5 1.0 0.5 0.0
ppm

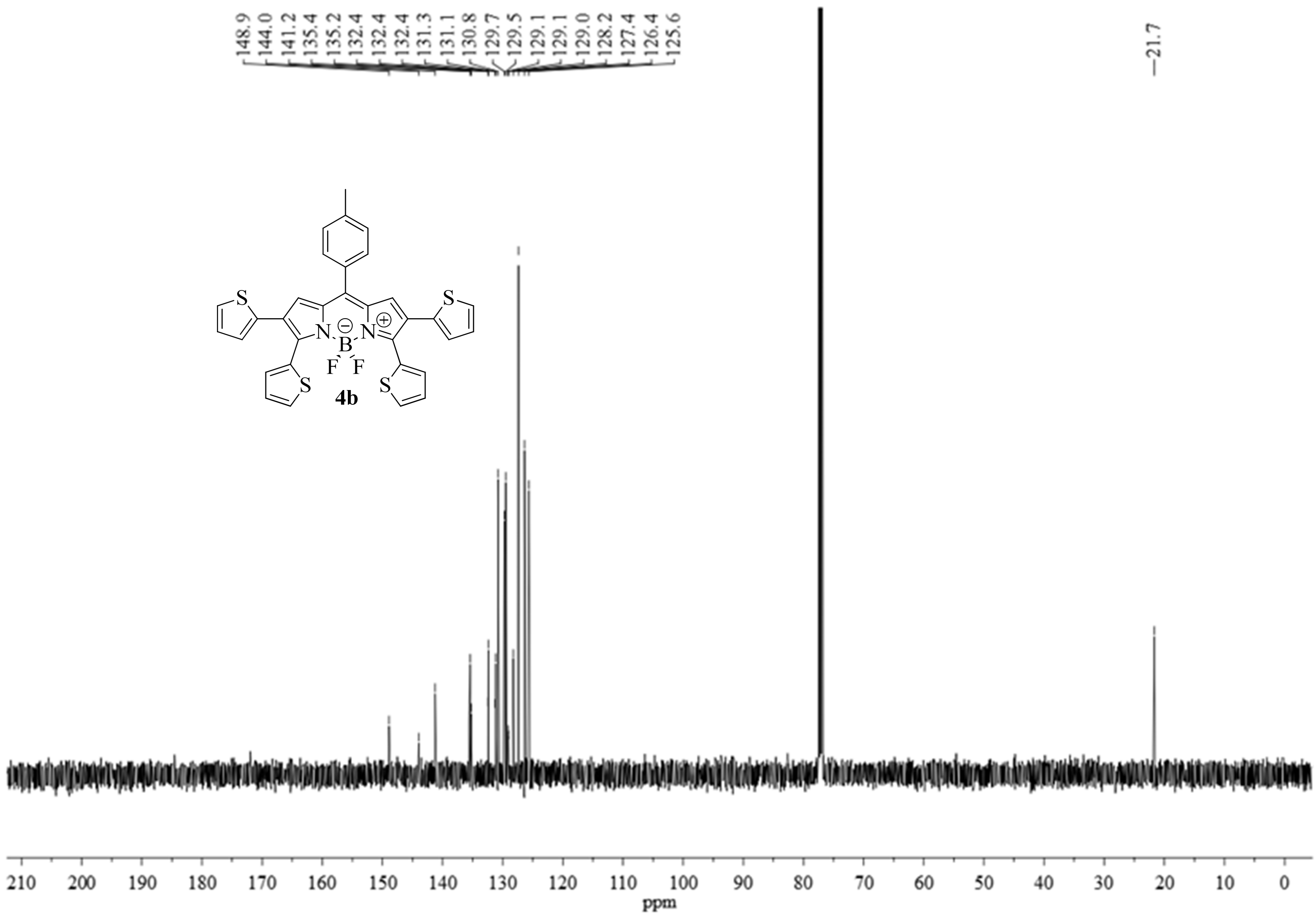
148.9
144.0
141.2
135.4
135.2
132.4
132.4
132.4
131.3
131.1
130.8
129.7
129.5
129.1
129.1
129.0
128.2
127.4
126.4
125.6
21.7
4b
ppm

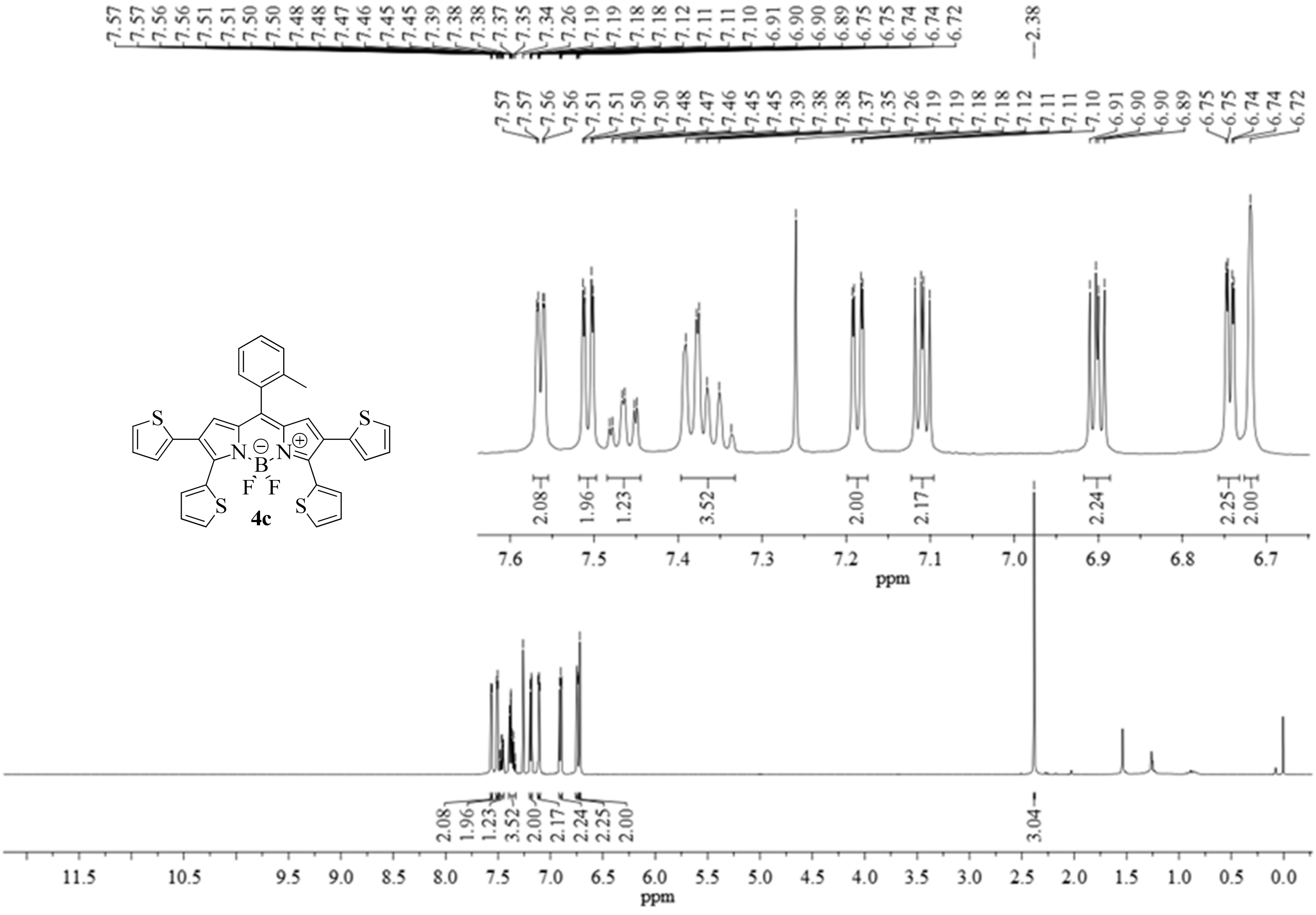
4c

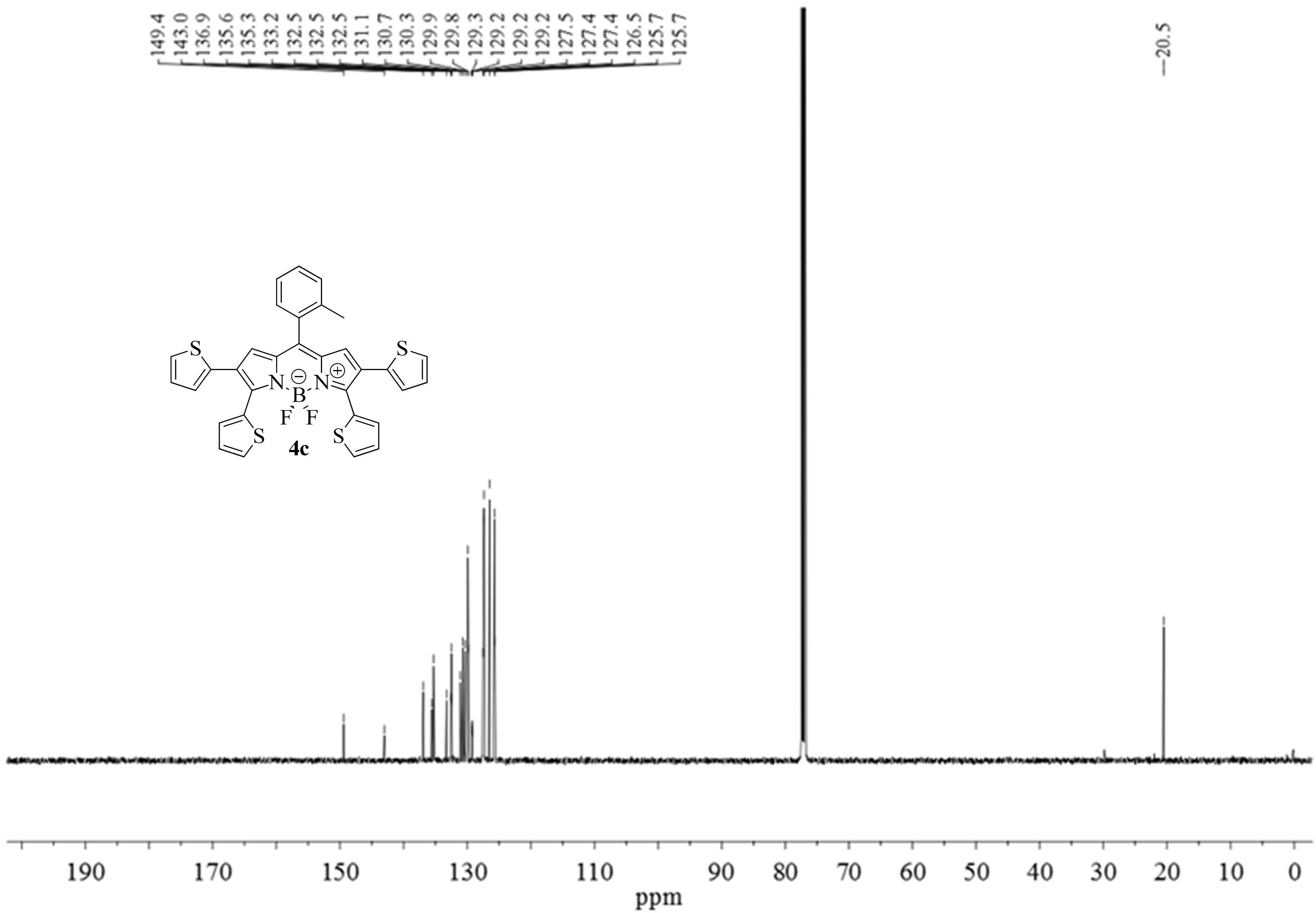
149.4
143.0
136.9
135.6
135.3
133.2
132.5
132.5
132.5
131.1
130.7
130.3
129.9
129.8
129.3
129.2
129.2
129.2
127.5
127.4
127.4
126.5
125.7
125.7
20.5
4c
190 170 150 130 110 90 80 70 60 50 40 30 20 10 0
ppm

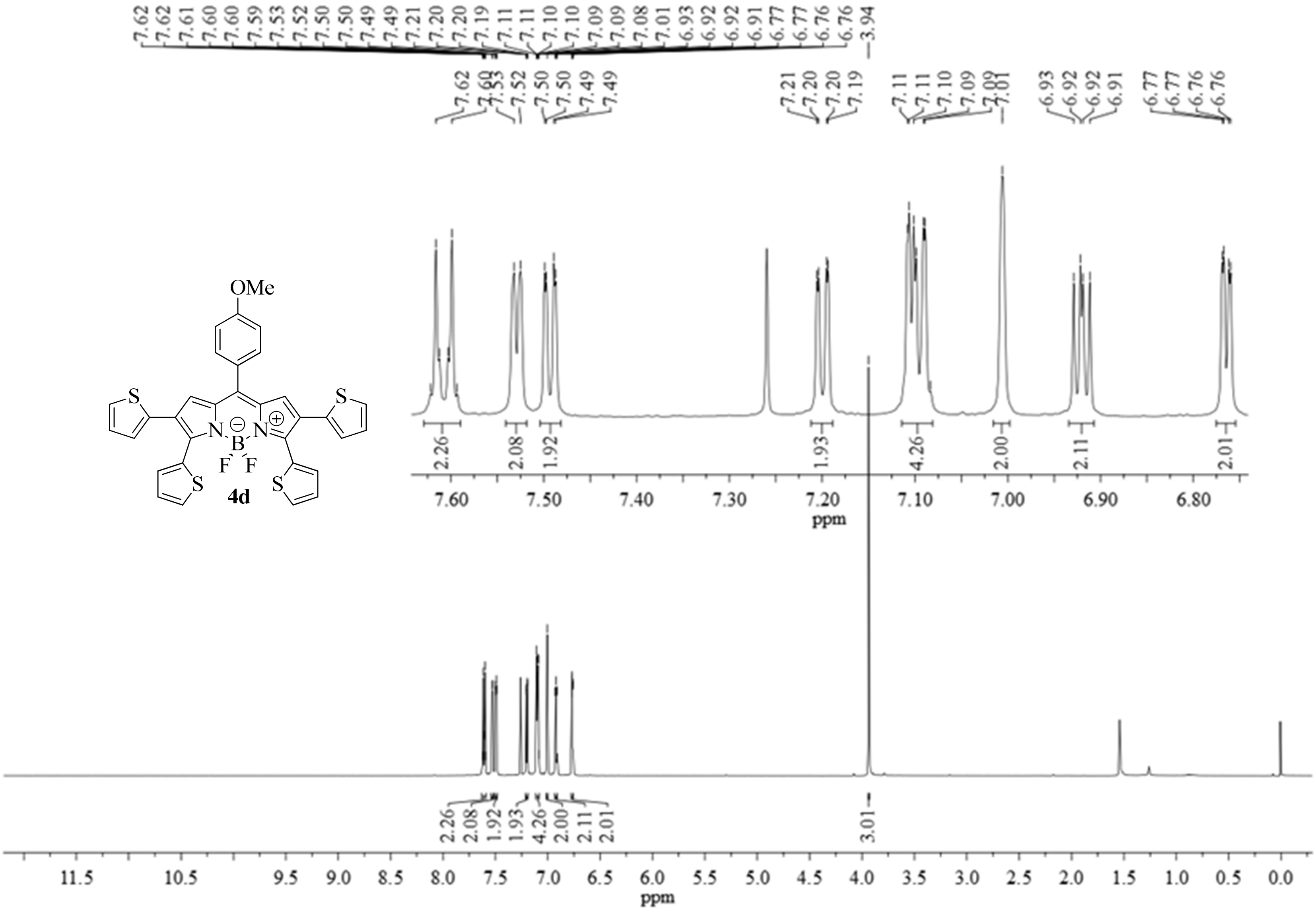
OMe
S
N
B
N
F
F
4d
2.26
2.08
1.92
1.93
4.26
2.00
2.11
2.01
3.01
ppm

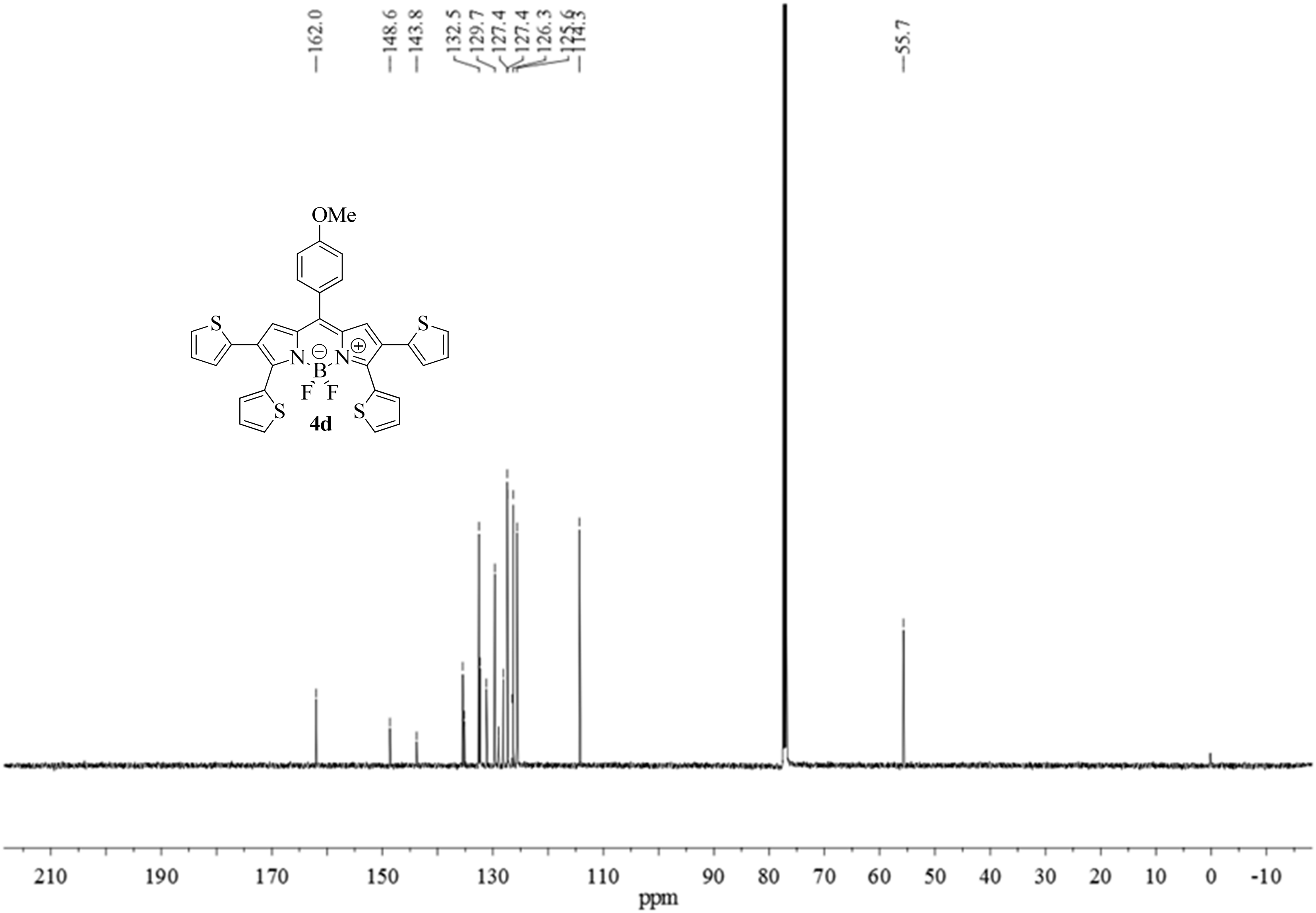
162.0
148.6
143.8
132.5
129.7
127.4
127.4
126.3
125.6
114.3
55.7
OMe
S
S
S
S
N
N
B
F
F
4d
210
190
170
150
130
110
90
80
70
60
50
40
30
20
10
0
-10
ppm

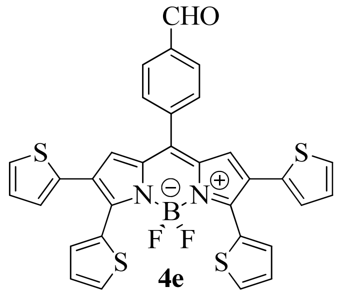

4e

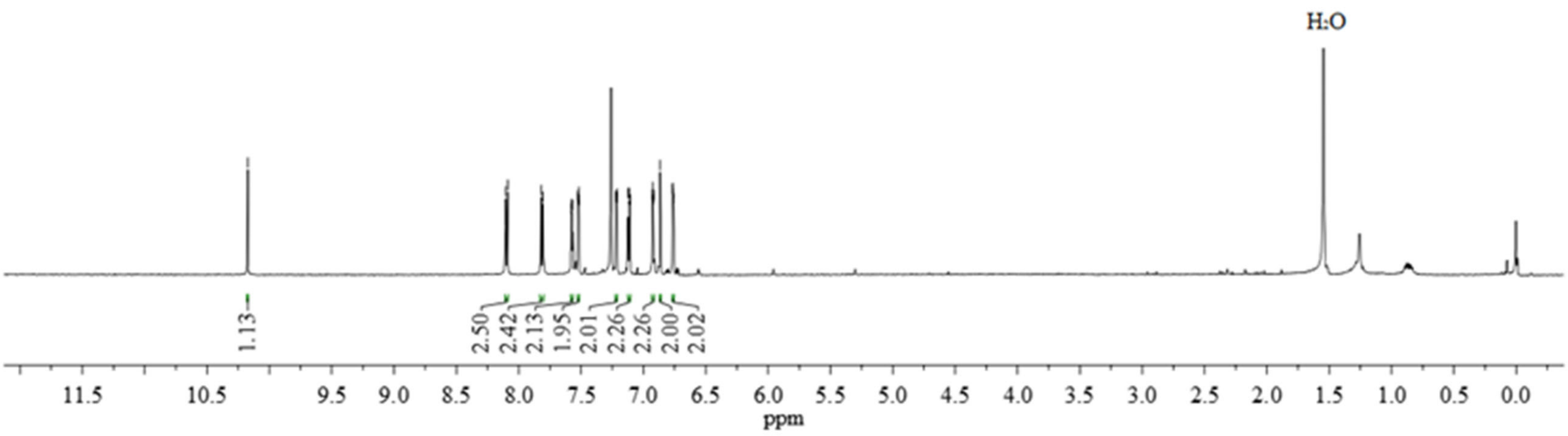
10.17
8.11
8.09
7.82
7.80
7.58
7.58
7.57
7.57
7.53
7.53
7.52
7.52
7.22
7.22
7.22
7.21
7.21
7.12
7.12
7.11
7.11
6.93
6.92
6.92
6.91
6.87
6.77
6.77
6.76
6.76
H2O
1.13
2.50
2.42
2.13
1.95
2.01
2.26
2.26
2.00
2.02
11.5
10.5
9.5
9.0
8.5
8.0
7.5
7.0
6.5
6.0
5.5
5.0
4.5
4.0
3.5
3.0
2.5
2.0
1.5
1.0
0.5
0.0
ppm

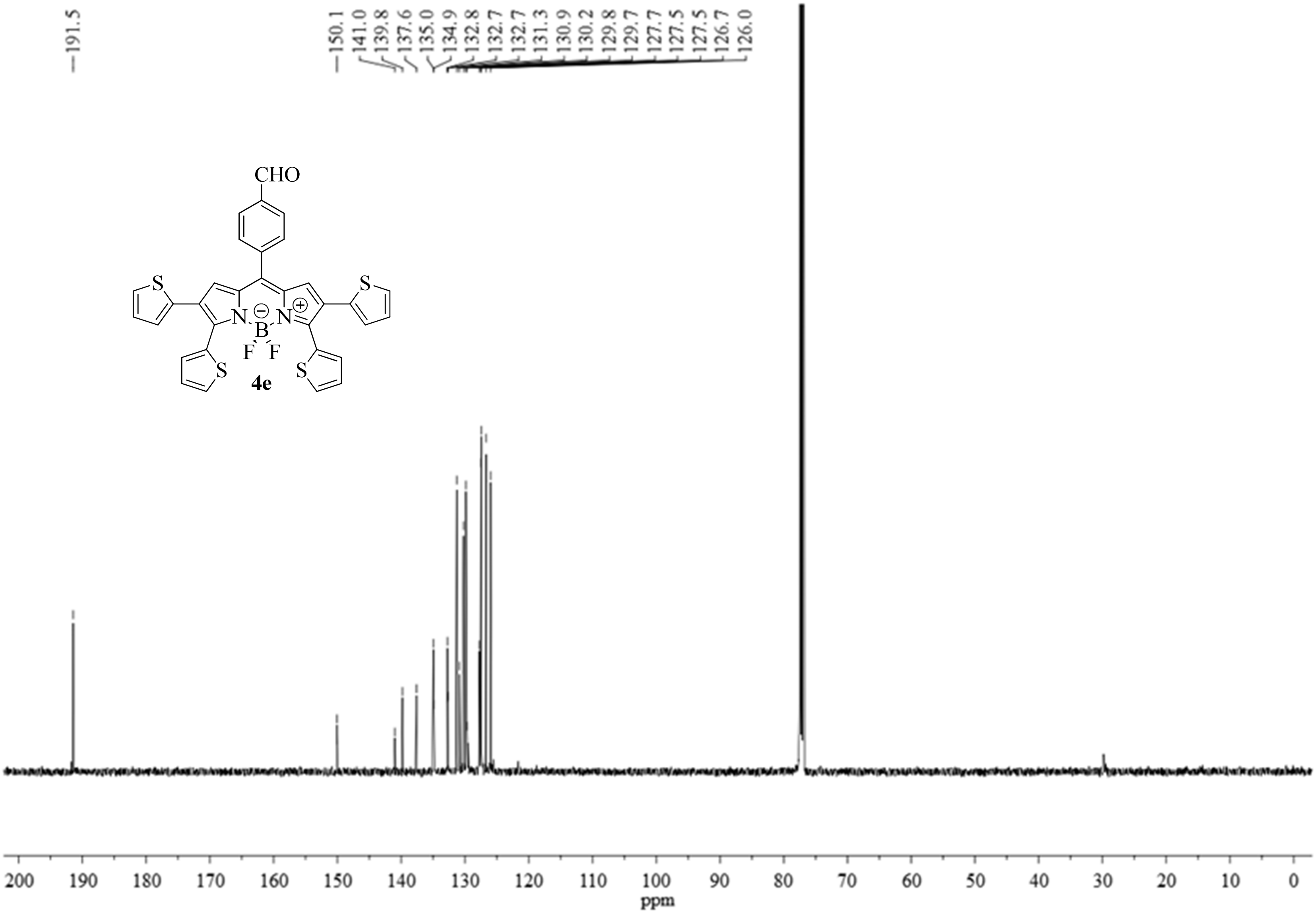

191.5
150.1
141.0
139.8
137.6
135.0
134.9
132.8
132.7
132.7
131.3
130.9
130.2
129.8
129.7
127.7
127.5
127.5
126.7
126.0
CHO
S
N
B
F
4e
200 190 180 170 160 150 140 130 120 110 100 90 80 70 60 50 40 30 20 10 0
ppm

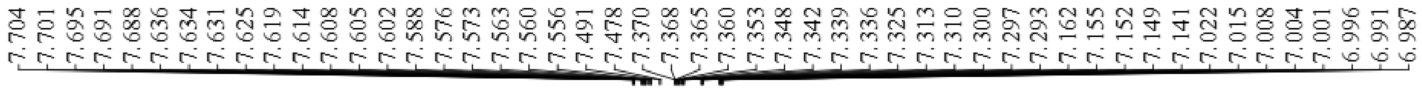
7.704
7.701
7.695
7.691
7.688
7.636
7.634
7.631
7.625
7.619
7.614
7.608
7.605
7.602
7.588
7.576
7.573
7.563
7.560
7.556
7.491
7.478
7.370
7.368
7.365
7.360
7.353
7.348
7.342
7.339
7.336
7.325
7.313
7.310
7.300
7.297
7.293
7.162
7.155
7.152
7.149
7.141
7.022
7.015
7.008
7.004
7.001
6.996
6.991
6.987

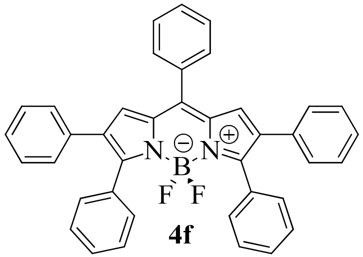
N
B
N
F
F

4f

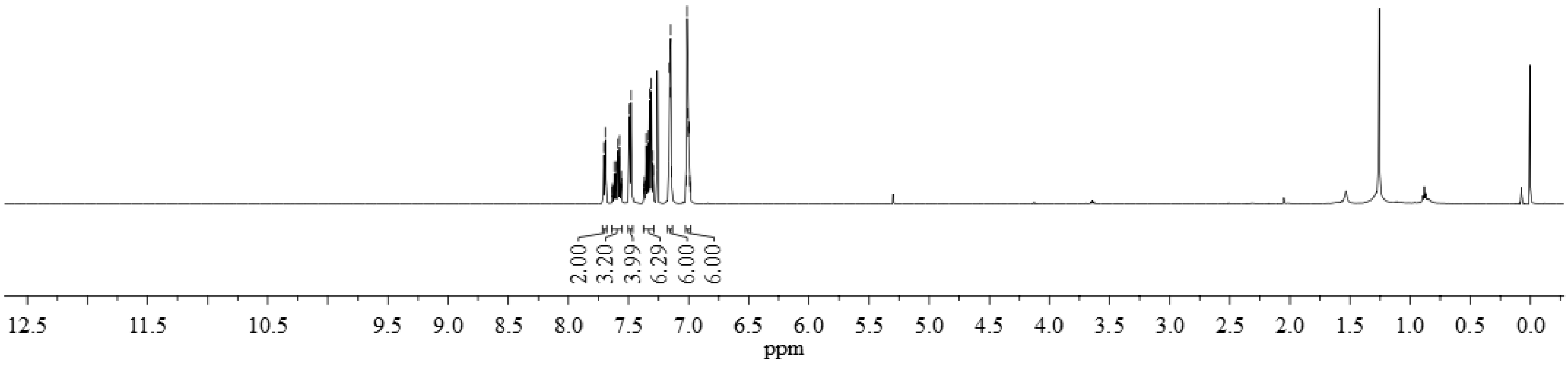
2.00
3.20
3.99
6.29
6.00
6.00
12.5
11.5
10.5
9.5
9.0
8.5
8.0
7.5
7.0
6.5
6.0
5.5
5.0
4.5
4.0
3.5
3.0
2.5
2.0
1.5
1.0
0.5
0.0
ppm

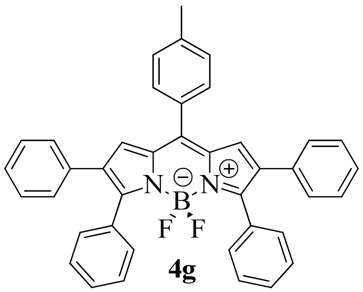

4g

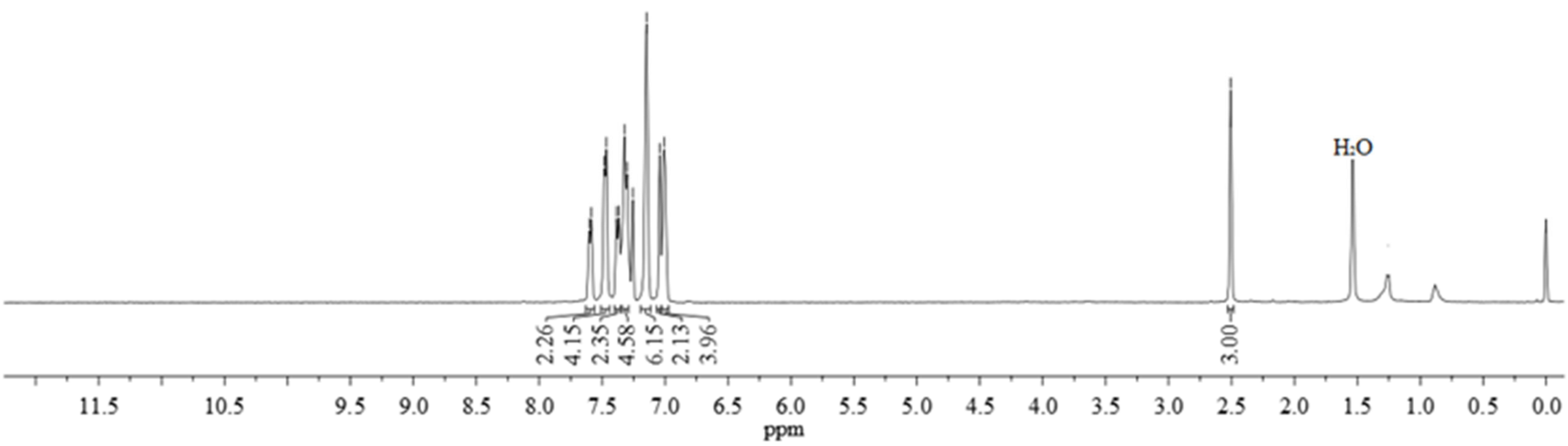
7.603
7.585
7.482
7.467
7.385
7.366
7.323
7.302
7.256
7.148
7.041
7.007
2.506
H2O
2.26
4.15
2.35
4.58
6.15
2.13
3.96
3.00
11.5
10.5
9.5
9.0
8.5
8.0
7.5
7.0
6.5
6.0
5.5
5.0
4.5
4.0
3.5
3.0
2.5
2.0
1.5
1.0
0.5
0.0
ppm

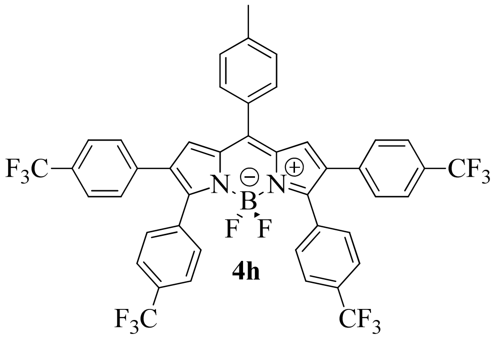
F3C
CF3
N
B
N
F
F
F3C
CF3

4h

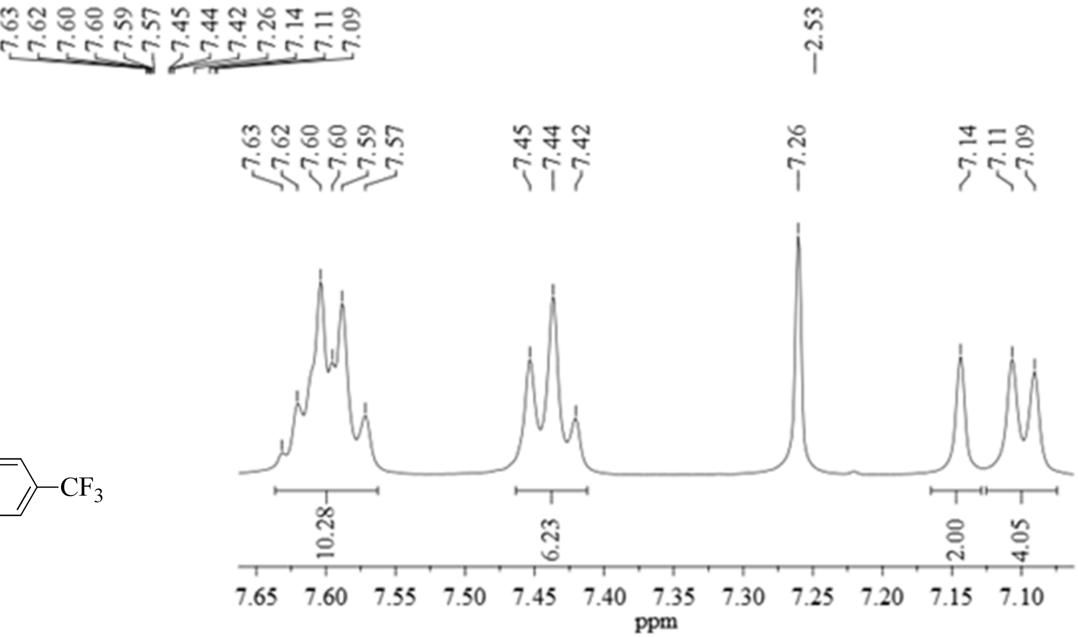
7.63
7.62
7.60
7.60
7.59
7.57
7.45
7.44
7.42
7.26
7.14
7.11
7.09
2.53
10.28
6.23
2.00
4.05
ppm

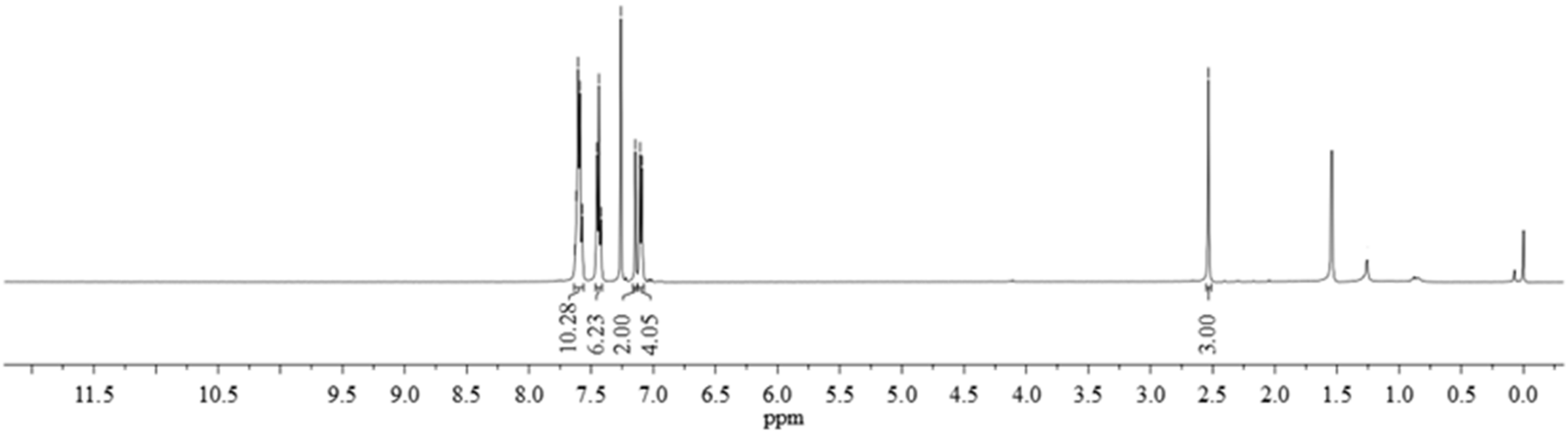
10.28
6.23
2.00
4.05
3.00
ppm

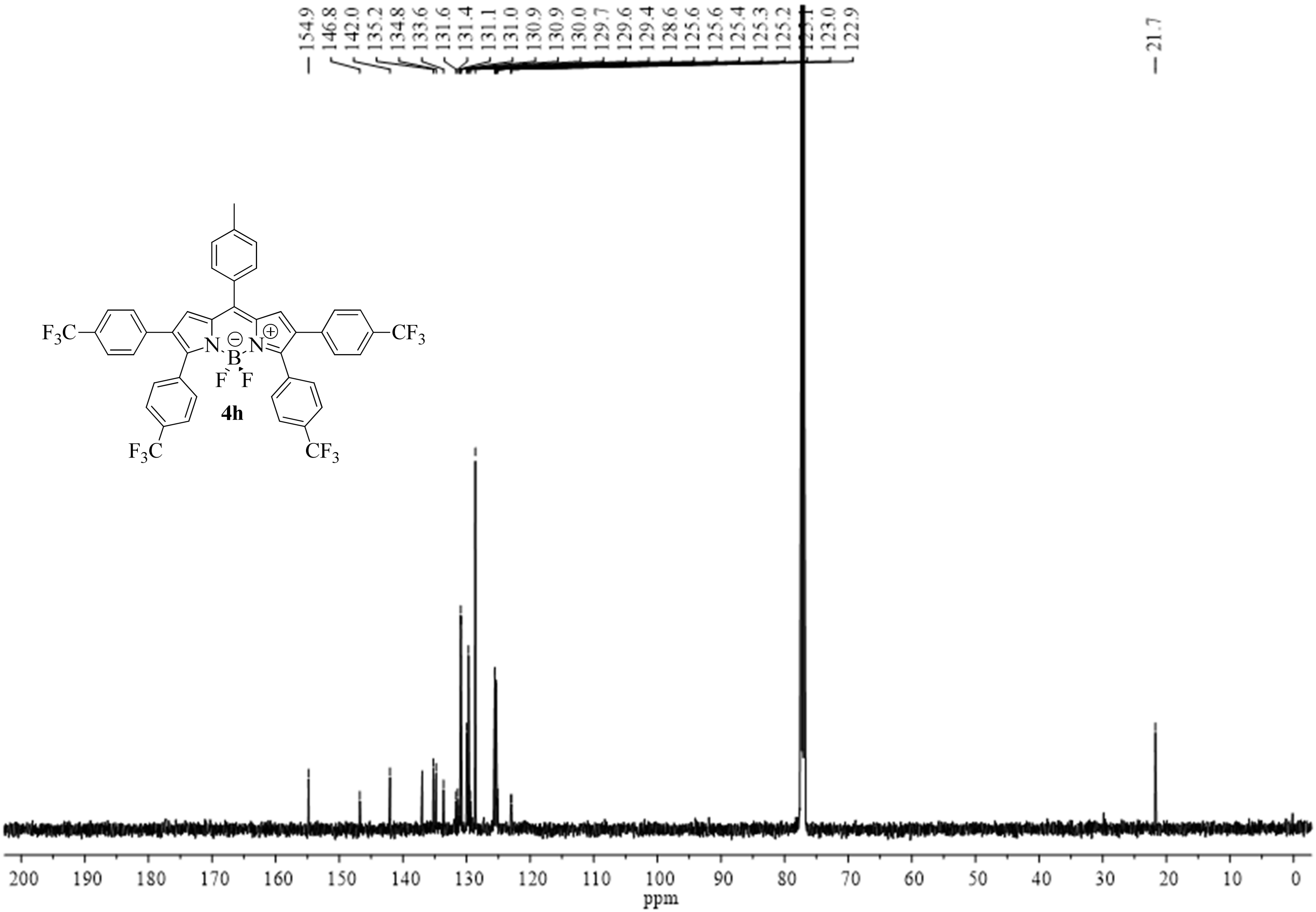

154.9
146.8
142.0
135.2
134.8
133.6
131.6
131.4
131.1
131.0
130.9
130.9
130.0
129.7
129.6
129.4
128.6
125.6
125.6
125.4
125.3
125.2
123.0
122.9
21.7
F3C
CF3
N
B
N
F
F
4h
200
190
180
170
160
150
140
130
120
110
100
90
80
70
60
50
40
30
20
10
0
ppm

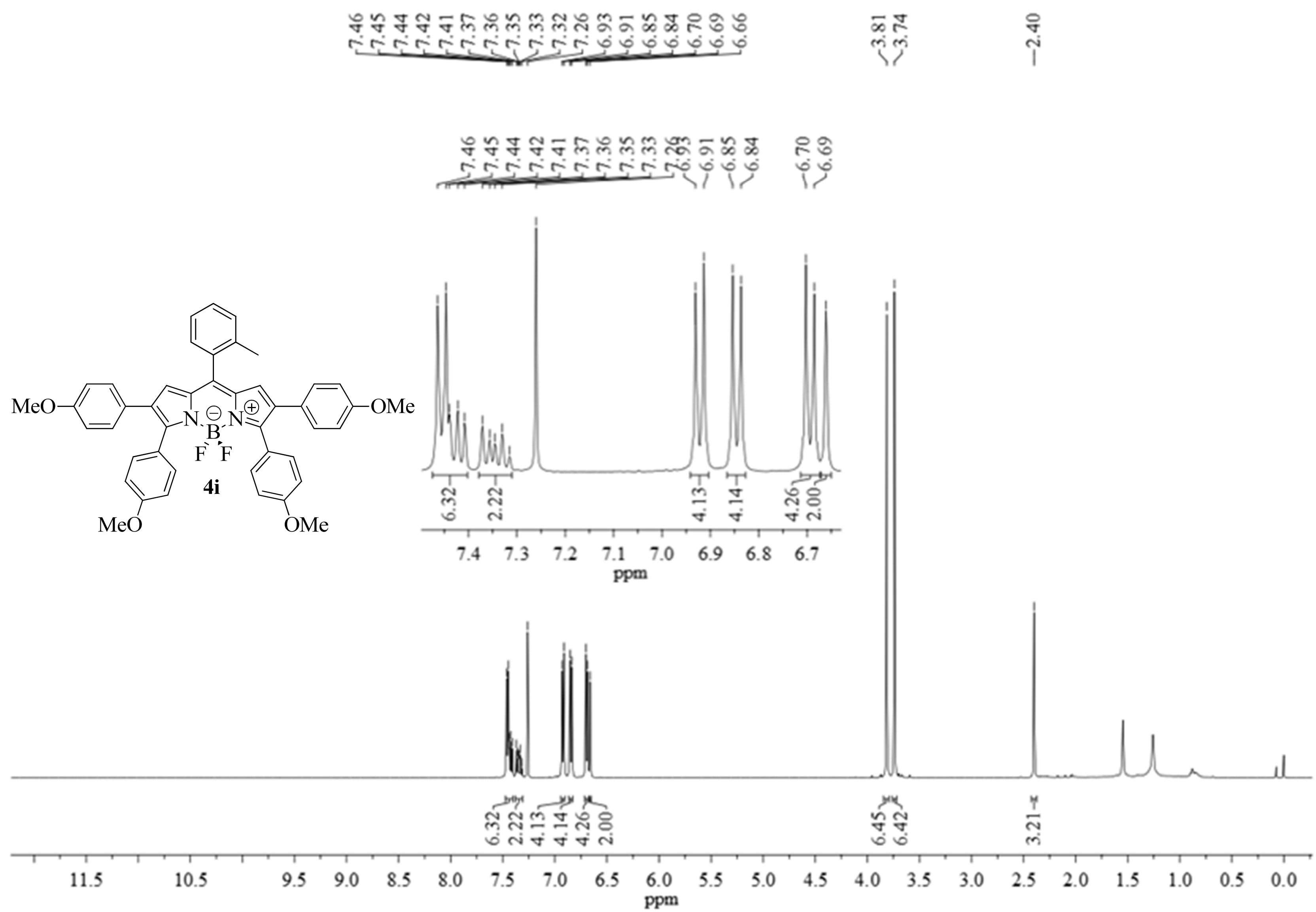
7.46
7.45
7.44
7.42
7.41
7.37
7.36
7.35
7.33
7.32
7.26
6.93
6.91
6.85
6.84
6.70
6.69
6.66
3.81
3.74
2.40
MeO
OMe
N
B
F
F
4i
6.32
2.22
4.13
4.14
4.26
2.00
6.45
6.42
3.21
ppm

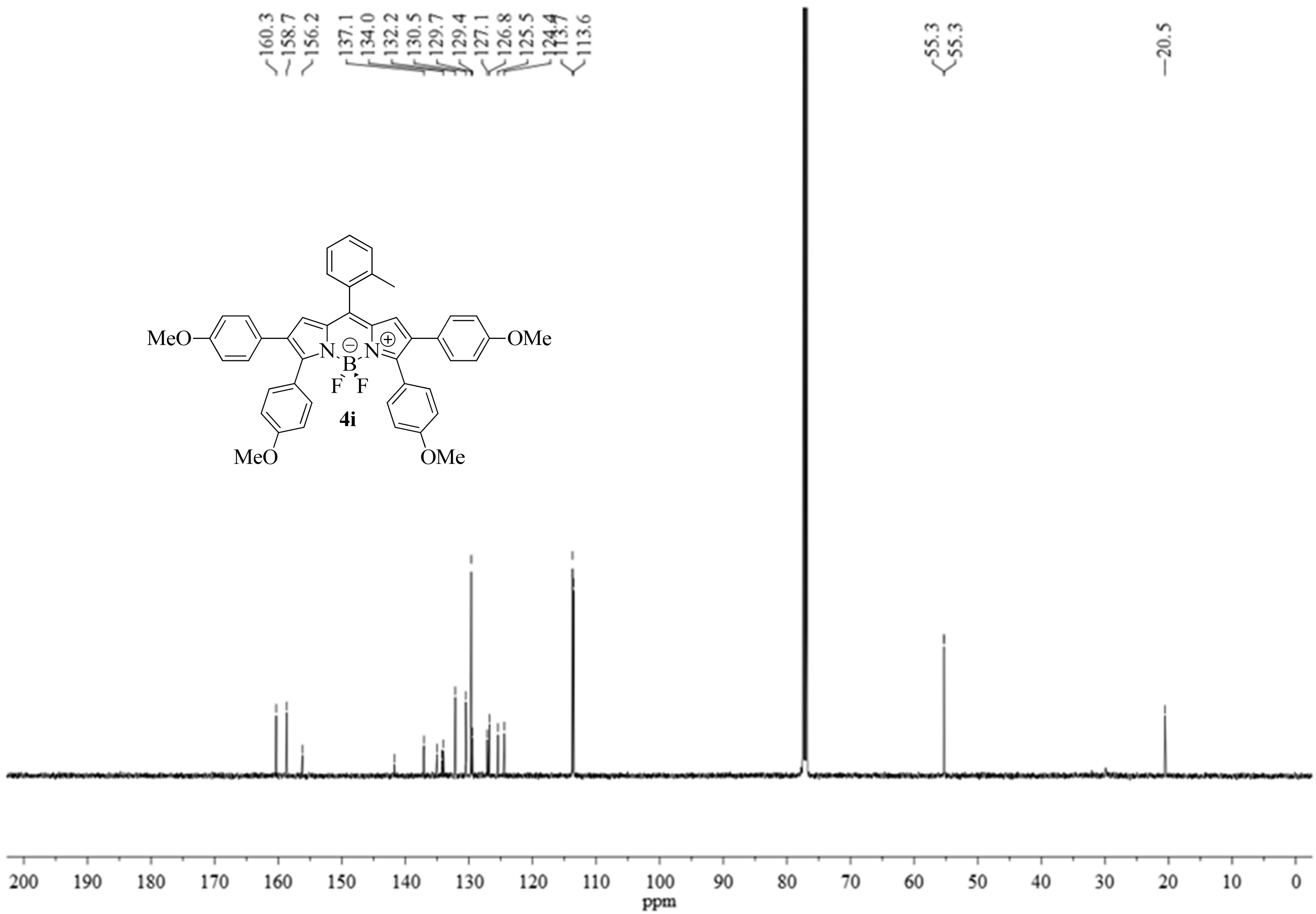
160.3
158.7
156.2
137.1
134.0
132.2
130.5
129.7
129.4
127.1
126.8
125.5
124.4
113.7
113.6
55.3
55.3
20.5
MeO
OMe
MeO
OMe
N
B
N
F
F
4i
ppm

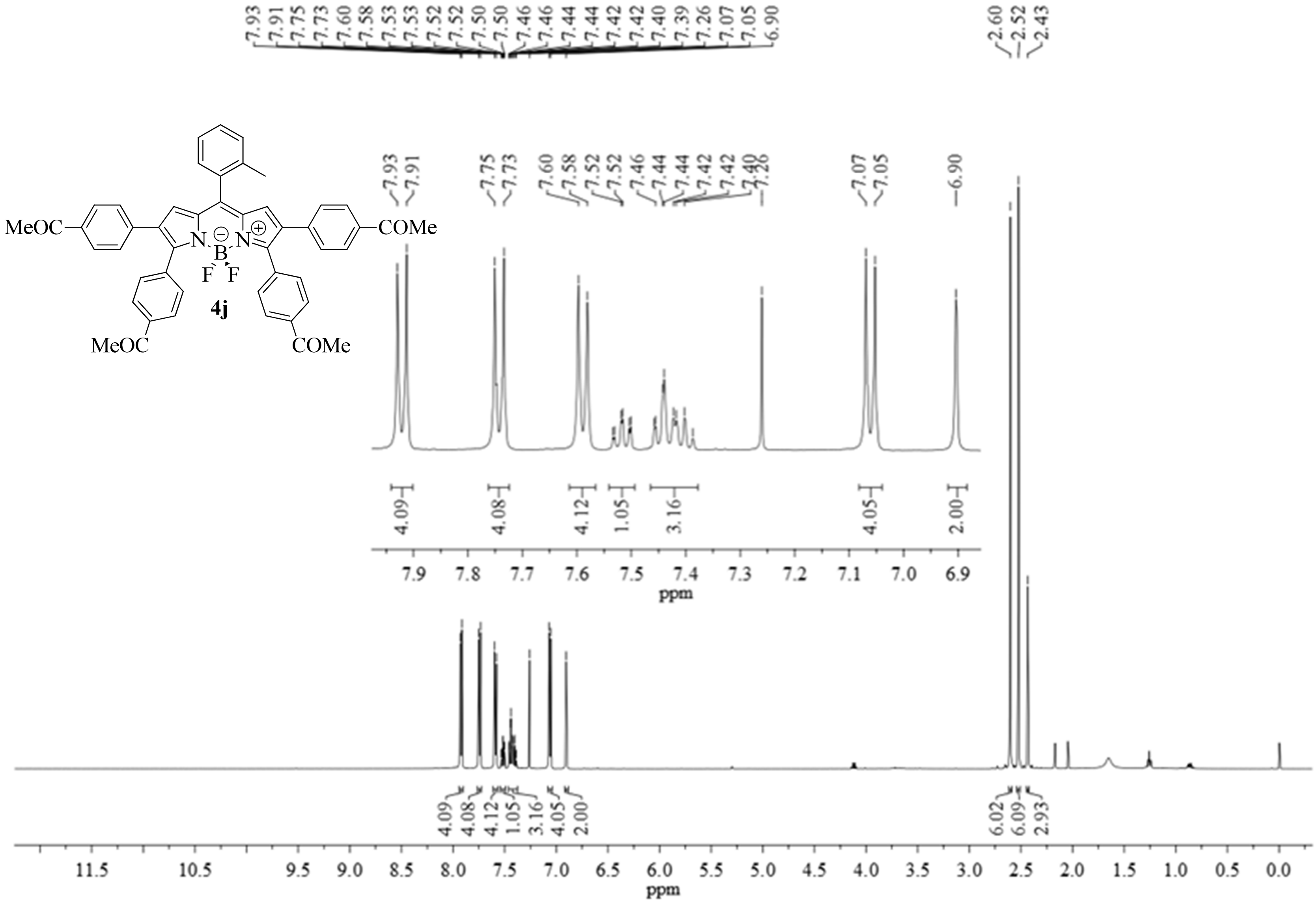
MeOC
COMe
MeOC
COMe
N
B
F
F
N
4j
ppm
ppm

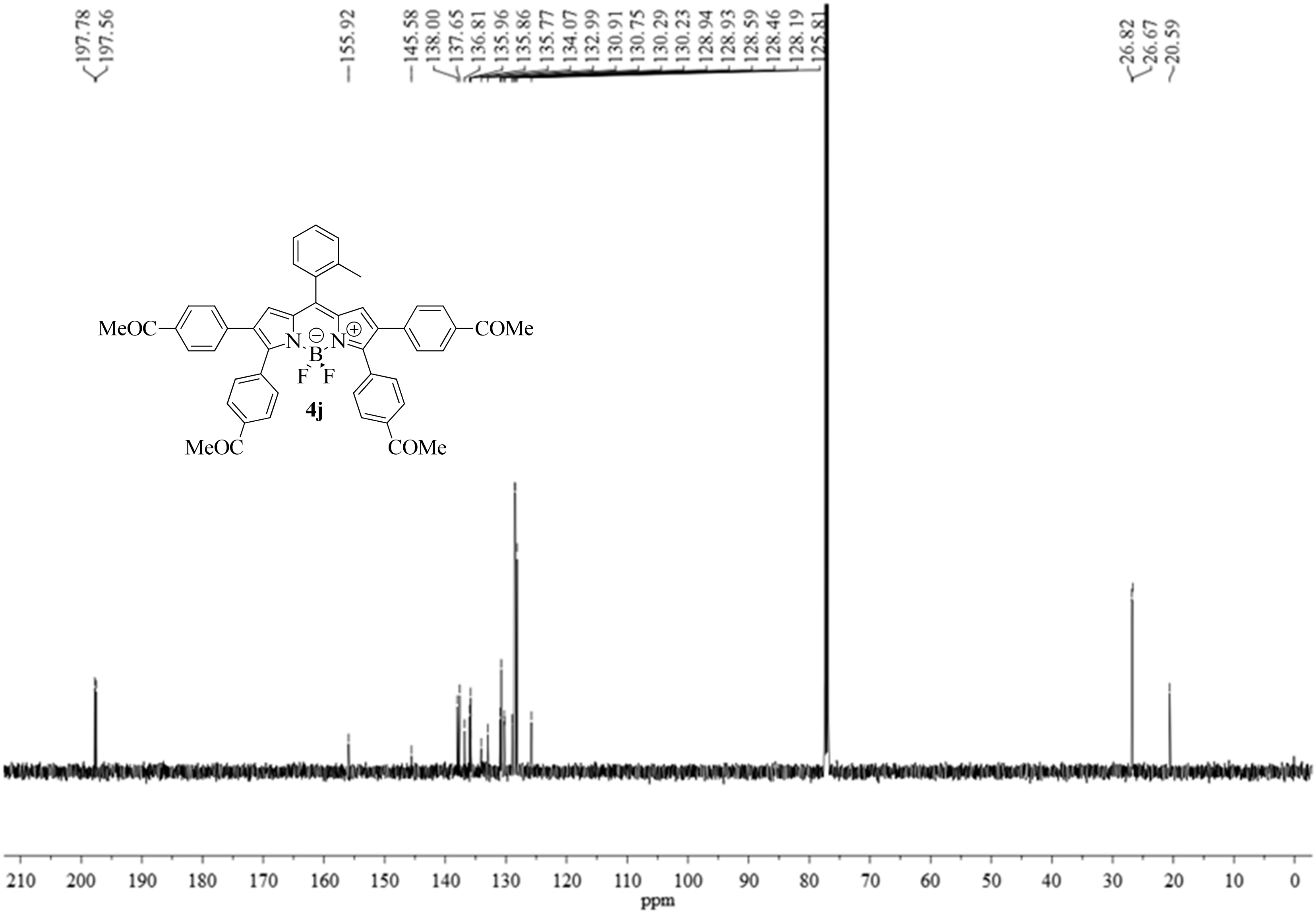
197.78
197.56
155.92
145.58
138.00
137.65
136.81
135.96
135.86
135.77
134.07
132.99
130.91
130.75
130.29
130.23
128.94
128.93
128.59
128.46
128.19
125.81
26.82
26.67
20.59
MeOC
COMe
N
B
F
F
4j
MeOC
COMe
210 200 190 180 170 160 150 140 130 120 110 100 90 80 70 60 50 40 30 20 10 0
ppm

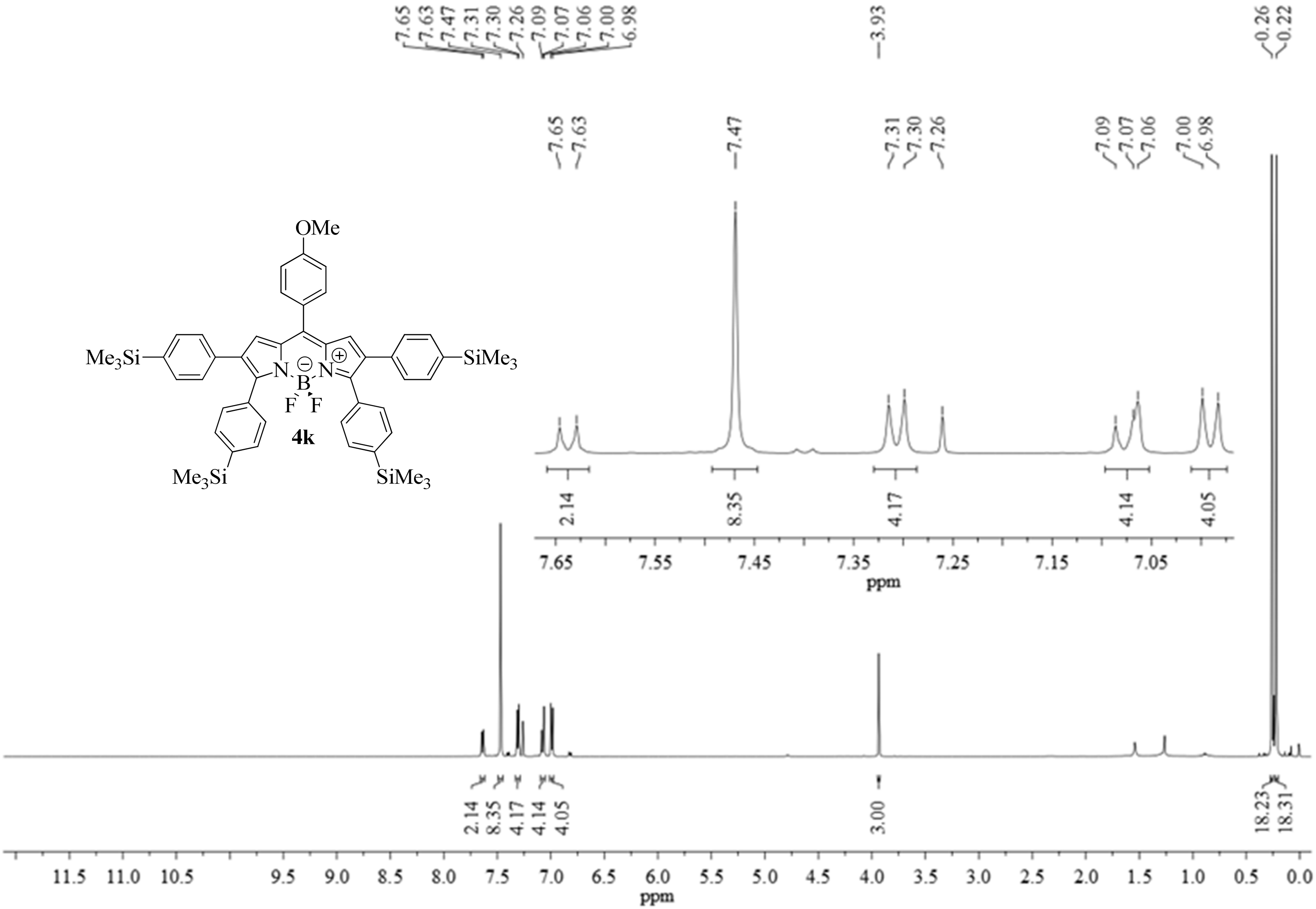

7.65
7.63
7.47
7.31
7.30
7.26
7.09
7.07
7.06
7.00
6.98
3.93
0.26
0.22
OMe
Me3Si
SiMe3
N
B
F
F
N
4k
2.14
8.35
4.17
4.14
4.05
3.00
18.23
18.31
ppm

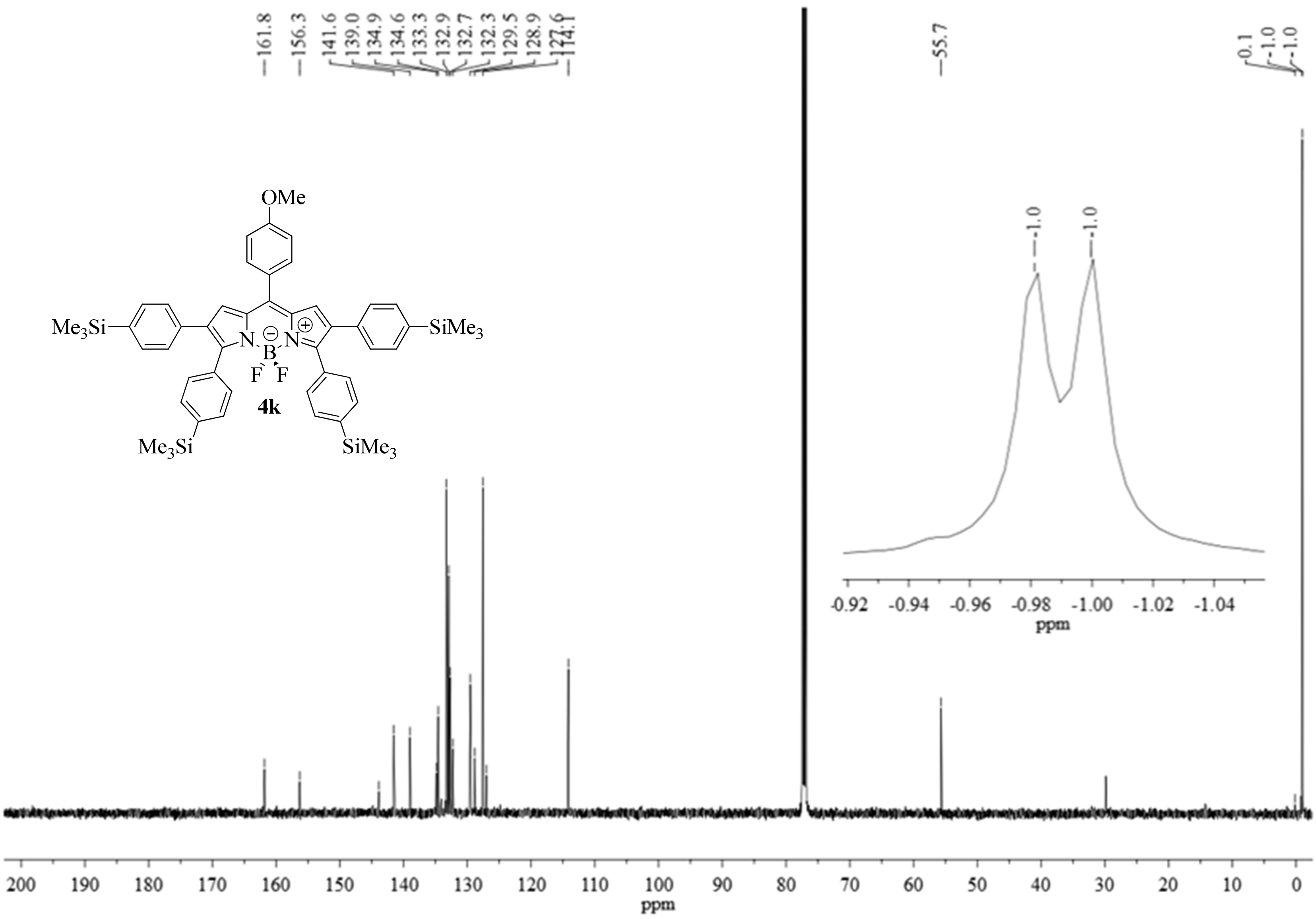

161.8
156.3
141.6
139.0
134.9
134.6
133.3
132.9
132.7
132.3
129.5
128.9
127.6
114.1
55.7
0.1
-1.0
-1.0
OMe
Me_3Si
$SiMe_3$
N
B
N
F
F
4k
Me_3Si
$SiMe_3$
-1.0
-1.0
-0.92
-0.94
-0.96
-0.98
-1.00
-1.02
-1.04
ppm
200
190
180
170
160
150
140
130
120
110
100
90
80
70
60
50
40
30
20
10
0
ppm

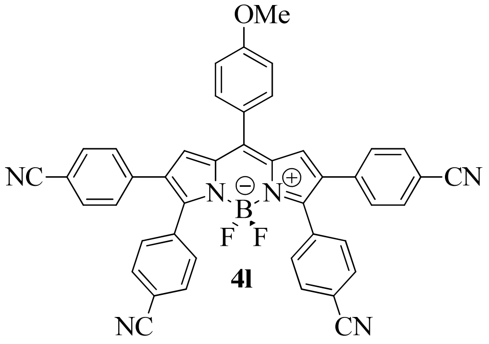
OMe
NC
CN
N
B
N
F
F
NC
CN

4l

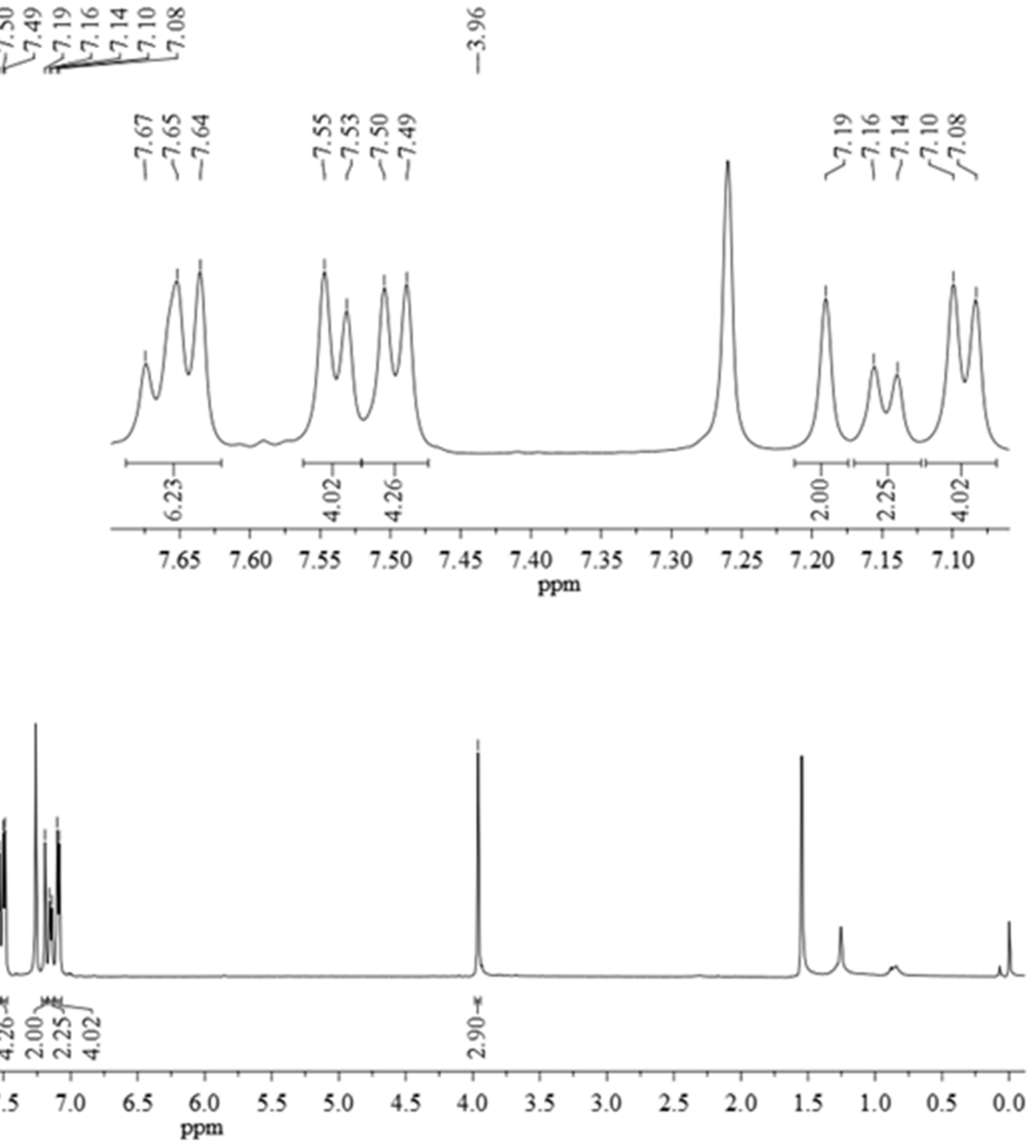
7.67
7.65
7.64
7.55
7.53
7.50
7.49
7.19
7.16
7.14
7.10
7.08
3.96
6.23
4.02
4.26
2.00
2.25
4.02
2.90
ppm
